# Nanotechnology for Dentistry: Prospects and Applications

**DOI:** 10.3390/nano13142130

**Published:** 2023-07-22

**Authors:** Arleta Glowacka-Sobotta, Daniel Ziental, Beata Czarczynska-Goslinska, Maciej Michalak, Marcin Wysocki, Emre Güzel, Lukasz Sobotta

**Affiliations:** 1 Chair and Department of Orthodontics and Temporomandibular Disorders, Poznan University of Medical Sciences, Bukowska 70, 60-812 Poznan, Poland; aglow@ump.edu.pl; 2 Chair and Department of Inorganic and Analytical Chemistry, Poznan University of Medical Sciences, Rokietnicka 3, 60-806 Poznan, Poland; dziental@ump.edu.pl (D.Z.); michalakm500@gmail.com (M.M.); marcin.wysocki@student.ump.edu.pl (M.W.); 3 Chair and Department of Pharmaceutical Technology, Poznan University of Medical Sciences, Grunwaldzka 6, 60-780 Poznan, Poland; bgoslinska@ump.edu.pl; 4 Department of Engineering Fundamental Sciences, Sakarya University of Applied Sciences, 54050 Sakarya, Türkiye; eguzel@subu.edu.tr; 5 Biomedical Technologies Application and Research Center (BIYOTAM), Sakarya University of Applied Sciences, 54050 Sakarya, Türkiye

**Keywords:** composites, nanoparticles, photodynamic therapy, photosensitizer, antibacterial nanoagents, nanotechnology, re-mineralization, caries, oral cancer, oral bacteria

## Abstract

In the XXI century, application of nanostructures in oral medicine has become common. In oral medicine, using nanostructures for the treatment of dental caries constitutes a great challenge. There are extensive studies on the implementation of nanomaterials to dental composites in order to improve their properties, e.g., their adhesive strength. Moreover, nanostructures are helpful in dental implant applications as well as in maxillofacial surgery for accelerated healing, promoting osseointegration, and others. Dental personal care products are an important part of oral medicine where nanomaterials are increasingly used, e.g., toothpaste for hypersensitivity. Nowadays, nanoparticles such as macrocycles are used in different formulations for early cancer diagnosis in the oral area. Cancer of the oral cavity—human squamous carcinoma—is the sixth leading cause of death. Detection in the early stage offers the best chance at total cure. Along with diagnosis, macrocycles are used for photodynamic mechanism-based treatments, which possess many advantages, such as protecting healthy tissues and producing good cosmetic results. Application of nanostructures in medicine carries potential risks, like long-term influence of toxicity on body, which need to be studied further. The introduction and development of nanotechnologies and nanomaterials are no longer part of a hypothetical future, but an increasingly important element of today’s medicine.

## 1. Introduction

Nanostructures refer to objects that have at least one of their dimension in a three-dimensional space in the range of (1–100 nm), so called the nanoscale [1,2]. Due to their unique properties, i.e., high surface-to-volume ratios, and high compressive and flexural strength, scientists have researched different application methods and industries which would benefit from the use of these materials [2,3]. Currently, a dynamic development in the use of nanotechnology is observed in all areas of medicine. It is used, among others, in medical diagnostics (as markers for detection and identification of various diseases), cancer therapy (for targeted delivery of anti-cancer drugs), gene therapy (e.g., DNA or RNA nanovectors), treatment of infections (modifying the release of antibiotics or the antibacterial effect of molecules per se, e.g., nanosilver), tissue and organ regeneration (nanomaterials can be used to stimulate tissue growth and regeneration and to create biomaterials), medical imaging (as contrast agents in imaging techniques), photodynamic techniques (carriers of photosensitizing substances), in neuroscience and many others [4,5,6,7,8,9]. The use of nanotechnology is associated with many positive effects for a patient. In many cases, more effective treatment is possible because of its use. Thanks to the targeted drug delivery system, it is possible to achieve higher concentrations of the drug at the site of action, while reducing the systemic side effects. The large share of nanotechnology in regenerative medicine also allows to achieve a significant reduction in convalescence time. Tissue reconstruction processes are accelerated due to the high biocompatibility of many new materials. All these aspects also lead to the development of individualized therapy based on precisely diagnosed needs of a patient [4,5,6].

A natural consequence of the development of nanomaterials and nanotechnologies in medicine is their entry into the field of dentistry and therapy of head and neck diseases. It is applied in the most basic areas related to oral personal care and also in advanced technologies related to bone reconstruction or cancer therapy. In the presented review, we discuss a wide spectrum of research in those areas. The industries of oral personal care products and medicine are the ones that have already benefited from the advantages of using nanomaterials. For instance, nanostructures found their role in improving dental prosthetics such as coating of the implants’ surface or the implants themselves, leading to better biocompatibility and the process of osseointegration [10,11]. Nanoparticles can also be found in much more trivial products such as toothpastes for hypersensitivity, mouth washes, bleaching gels or even sports drinks [5,6]. Nanostructures such as nano-hydroxyapatite (*n*-HA), nano-carbonate apatite (*n*-CAP), nano-carbonate substituted hydroxyapatite (nano-CHA), nano-fluorohydroxyapatite (*n*-FA) and nano-sodium trimetaphosphate (TMP) are also used as innovative and functional dental care products. Moreover, when used in toothpastes, they can perform re-mineralizing and anti-caries functions, combat dentin hypersensitivity and prevent enamel erosion [12,13,14,15,16,17,18].

Other important areas of nanotechnology development in the field of dentistry include nanofillers in dental composites. Nanofillers are being studied to improve the mechanical properties and promote re-mineralization of tooth structures. Among other materials, glass ionomers modified using resin with nanofillers and agents releasing fluorine or calcium ions are used. Antibacterial nanofillers such as zinc nanooxide (*n*-ZnO) and silver nanoparticles (*n*-Ag) are used [19,20]. Clay, silica and carbon nanotubes are other nanofillers that are being studied for their impact on the mechanical properties and aesthetics of dental composites. This review indicates that extensive research is still needed to optimize the mechanical properties and cytotoxicity of dental composite materials. Another significant topic is the development of endodontic treatment, which focuses on the treatment of diseases of the dental pulp. One of the more dynamically developing areas of nanotechnology dealing with this topic is photodynamic antimicrobial chemotherapy (PACT). Already, PACT-based antimicrobial systems such as PAD™ and Helbo^®^ have been introduced into dental practice. Ultrasonic irrigation with NaOCl is also being developed [21].

The effectiveness of PACT against periodontitis has been analyzed. This is a biofilm-related inflammatory periodontal disease and a major cause of tooth loss. It is primarily caused by a shift in the oral microflora towards Gram-negative pathogenic anaerobes. However, photodynamic therapy is not only limited to antibacterial but it is also used in anticancer applications. Porfimer sodium (Photofrin) and a hematoporphyrin derivative (HpD/Photofrin) have a particularly important place in the PDT of cancer, as well as the second-generation synthetic drug mTHPC (Foscan), which are used in the treatment of head and neck cancers. Photofrin has relatively poor tumor selectivity and limited absorption of red light while still being a well-penetrating drug. A new generation of chlorin photosensitizers (PSs) has been developed to improve selectivity, reduce exposure time and photosensitization. Derivatization of chlorin e6 (Photolon^®^) is becoming more and more popular in the treatment of superficial and deep lesions in the head and neck area, shortening the period of photosensitization after treatment [22,23].

Despite the enormous benefits of nanotechnology, knowledge about its use, even among surgeons, is relatively low. Some of the doctors use nanomaterials without even being aware of this fact [24]. This highlights the importance of disseminating knowledge about new treatment methods. The latest research presented in the review can expand knowledge in this area and it constitutes a handy compendium.

## 2. Nanostructure of Tooth

**Figure 1:**
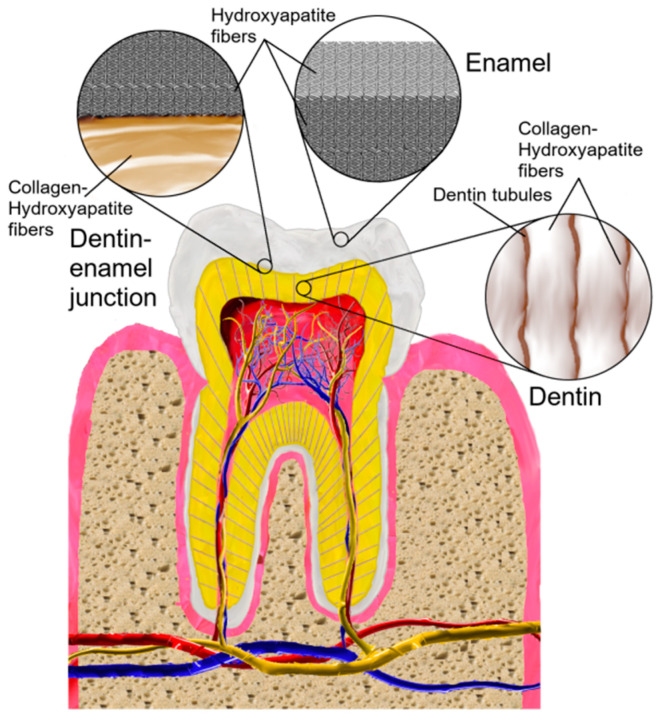
Nanostructure of a tooth.

Human teeth are built of tissues that constitute a hierarchical structure. The outer tissue of a tooth, the enamel, is a biocomposite that consists mainly of hydroxyapatite. High mineralization and its specific structure make enamel the hardest tissue of the human body (Figure 1) [25,26,27]. In enamel, hydroxyapatite forms crystallites with nanorod-like shape, having a vertical array. Such an alignment also results in anisotropic properties [25,27,28]. The enamel acts as an insulating barrier and protects the rest of the tooth from injuries due to physical, chemical or thermal forces [29].

The boundary between the enamel and dentine is called a dentin–enamel junction (DEJ). It is a place in which the orientations of the enamel and dentin nanostructures change [25]. The surface of the DEJ is formed in ridges, which probably increase the adhesion of enamel and dentine, and therefore, reduce shearing of the enamel while the tooth works [30]. Due to its unique mechanical properties, the junction can prevent traversing of cracks from enamel into dentin [26].

Dentin consists of hydroxyapatite and about 20–30% of collagen, which mainly contains the collagen-I fibrils. Therefore, the structure of dentin is mainly poly-crystalline and consists of plate-like crystallites. Thanks to that and the fixed orientation of collagen fibers (aligned perpendicularly to crystallites present in enamel), the dentin also possesses anisotropic properties [25,26,28,31,32]. Due to less mineralization and less brittle construction, dentine acts as a support for the enamel [26]. Plate-like crystallites of dentin tend to appear more likely near the junction [25]. Their orientation differs, depending on the region of dentin: near the junction, they are rather horizontally oriented, whereas near the pulp, their orientation tends to be vertical [25]. There are also suggestions that those crystallites are covered with some hydrated layer [32]. It appears that the hardness of the dentin is somewhere between the hardness of an enamel and a bone, although it still has an elastic property, preventing fracture of the tooth [26]. Dentin is considered to be the most abundant tissue in the human tooth, thus, its structure alterations may affect tooth fragility [32].

## 3. Restorative Dentistry (Dental Materials)

**Figure 2:**
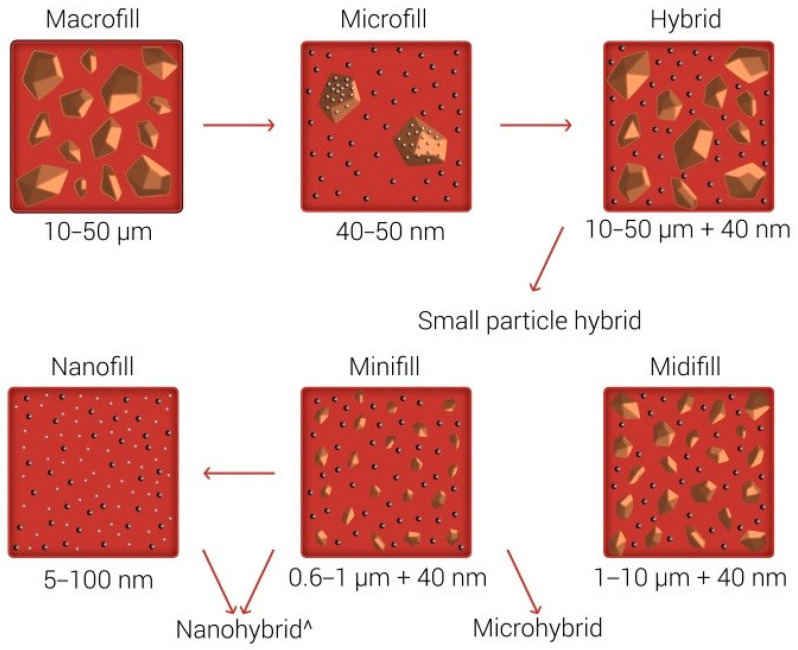
Division of dental composite fillers [35,36]. Adapted with permission from Ferracane, Dental Materials; published by Elsevier, 2011.

One of the common disorders in the oral cavity is dental caries. Caries is caused by the acid demineralization process of the tooth’s hard tissues—dentin and enamel. It leads to dental matrix destruction via acidic dissolving of hydroxyapatite. Bacterial flora of the oral cavity (*Streptococcus mutans*, *Streptococcus sanquis*, *Actinomyces* and *Lactobacillae*) is responsible for the decrease in pH. The microorganisms metabolize their primary energy source—simple sugars (i.e., glucose, which is delivered with food)—in the biochemistry pathways via lactic acid, which is the main agent of dental hard tissue disintegration. The other main factors for caries formation are diet and personal predisposition. Authors have mentioned over fifty factors responsible for caries development [33]. The symptom at the beginning of the process is a white spot on the surface of the tooth, which then turns into a darker color and finally becomes brown or black. The tooth tissue gradually softens and dissolves, turning into cavity [33]. After removing the destroyed surfaces from the soft material and bacteria as much as possible, the cavity is refilled with restorative materials. The new dental restorative materials should fulfill a few requirements: (i) lack of toxicity, (ii) inhibit bacterial growth, (iii) provide good separation of the oral environment and be placed beneath dental structures, (iv) repair destroyed tooth structures, (v) similar mechanical properties to dental structures, (vi) properties enabling tooth shape restoration, and (vii) aesthetic appearance [34]. Dental restorative materials can be divided according to chemical structures: (i) resin composite, (ii) polyacid-modified resin composite, (iii) glass ionomer types of cements and (iv) resin-modified glass ionomer cement. It can be also divided according to filler particles used: (i) macrofill, (ii) microfill, (iii) hybrid, (iv) midifill, (v) minifill and (vi) nanofill (Figure 2) [35,36].

Using nanofillers facilitates the incorporation of new intelligent tools into dental treatment protocols. Extensive research is performed to develop fillers, which can enhance the mechanical properties of a composite material such as shrinkage profile, flexural, tensile strength, microhardness, toughness, and resistance to wear. If all the aforementioned properties are not adequate, clinical use of composites become problematic. New intelligent composites possess the ability to re-mineralize dental structures and inhibit bacterial plaque growth, and they even exhibit some mechanical properties that are better than those of dentine or enamel [20].

**Figure 3:**
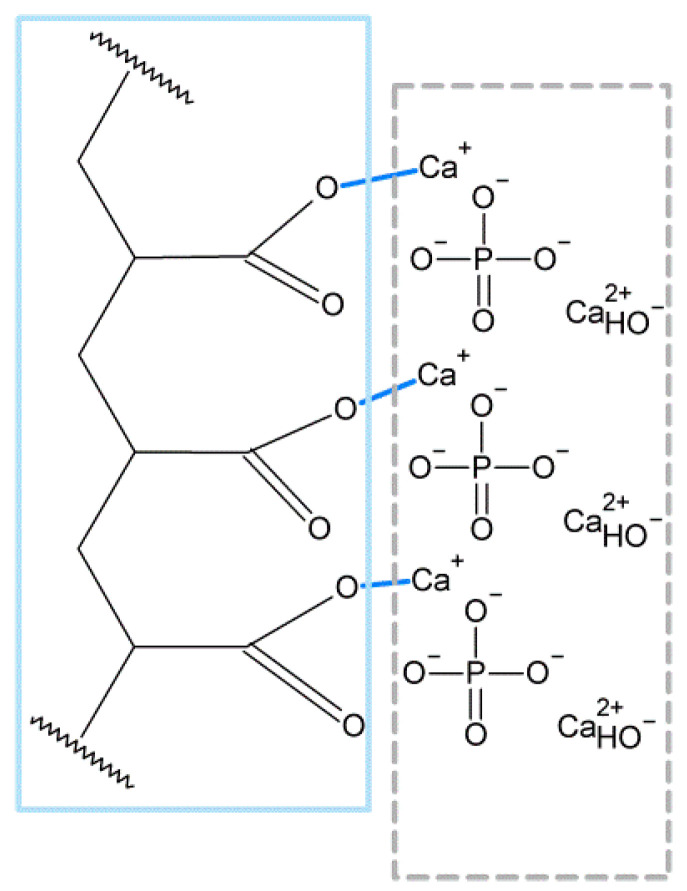
Binding of the polycarboxylate and hydroxyapatite.

The usage of restoration depends on its adhesion to tooth structures. Therefore, in clinical practice, there are two strategies: direct connection of restoration and tooth, or restoration through adhesive substances (bond) to achieve better adhesives’ toughness. Researchers have studied the adhesion strength of nanofilled seals from resin-modified glass ionomer groups in comparison to non-nanofilled ones. Authors have concluded that the bonding mechanism of nano-modified seal to dentin/enamel is similar to non-nanofilled resin-modified glass ionomer. The mechanism is based on the formation of a polycarboxylate bond between the polycarboxylic acid of nano-modified seal and the calcium ions from hydroxyapatite (Figure 3) in a micro-mechanical interlocking way. It was expected that nanofilled glass ionomer would reveal higher bonding strength due to the reaction of the nanofilled particle with the dentin/enamel structures. Surprisingly, the strength of bonding was at the same magnitude for both types of glass ionomers [37,38].

Demineralization and re-mineralization processes of the tooth are physiological, but if the re-mineralization process is disrupted, the dental caries starts developing. To reduce and control the development of dental caries, fluoride or calcium ions-releasing agent was incorporated into dental practice for enhancing the re-mineralization process. In particular, re-mineralization functions are very important in the newly sealed tooth, especially on the surface between the filling and dentine [20]. Studies have been performed on the usability of nanoagents in re-mineralization of the aforementioned contact surface. It used nanosized (diameter about 100 nm) amorphous calcium phosphate fillers (ACPs) and silanized glass filler as co-fillers (diameter ca. 1.4 µm), and bisphenol glycidyl dimethacrylate (Bis-GMA) and triethylene glycol dimethacrylate (TEGMA) as the composite matrix. Authors have tested the different amounts of fillers and their impact on the mechanical properties and the release profiles of Ca^2+^ and PO_4_^3−^ ions. The results indicate that a high ion release level was observed at a relatively low nanofill concentration in the composite. For ACP concentration in the range of 10 to 15%, increase in ion release was linear, but at 20% of ACP, an intense increase in ion relief was noticed. The release profile at different pH values was also checked; the results indicate that the highest release was observed at pH = 4 (very cariogenic environment for tooth). Mechanical properties such as flexural strength and elastic modulus before and after immersion in solutions of different pH have been studied. The authors concluded that after immersion, the ACP nanocomposite (10% ACP, 65% glass and 15% ACP, 60% glass) revealed a period flexural strength of 80–120 MPa, whereas the commercially available nanocomposite (Heliomolar, Ivoclar, Mississauga, ON, Canada) achieved a ca. value of 70 MPa. The elastic modulus test showed similar dependence to flexural strength. For the ACP nanocomposite, 10–13 GPa values were obtained in comparison to the reference of 7 GPa. Unfortunately, at the ACP concentration of 20%, dramatically decreased values of flexural strength and elastic modulus were noted. Obtained data suggest high potential usability of newly designed restorative material in prevention of secondary caries formation at the seal–tooth contact surface [39].

Secondary caries can be combated not only by improving the re-mineralization process of tooth, but also through introducing antibacterial nanoagents into a composite. Nowadays, nano zinc oxide (*n*-ZnO) and nano silver particles (*n*-Ag) have garnered widespread interest as nano antibacterial agents. The mechanisms of action of these materials have not been fully understood yet. It is supposed that silver ions interact with the peptidoglycan cell wall and plasma membrane of bacteria. These interactions disturb the physiological role of the structures and finally lead to cell death. Moreover, it was noted that silver ions interfere with sulfhydryl groups of bacterial DNA preventing its replication. *n*-ZnO reacts with reactive oxygen species formation like H_2_O_2_, which modifies cell membrane functions. It also seems that zinc ions displace magnesium ions in bacterial enzymes, interrupting their function. The mentioned phenomenon provides a significant antibacterial effect [20]. Although the antibacterial activity of *n*-ZnO and *n*-Ag is well known, introduction of these materials into composite remains an unsolved scientific problem [40]. Recently, researchers have performed studies on the influence of new nanocomposites on bacteria viability and seal mechanical properties. Composites consisting of anodic porous alumina (APA), *n*-Ag, and *bis*-GMA and TEGDMA as matrix were prepared. Authors confirmed high potential of the APA composite as a platform for controlled *n*-Ag release [19]. Another research group has studied a similar composite against bacteria. The filler was replaced with nano silver particles coated with oleic acid. Results indicated a high antibacterial effect with improved mechanical properties (flexural strength of 140 MPa; flexural modulus of 13 GPa; and compressive strength of 347 MPa), especially for nanoparticles with a concentration of 50 ppm (wt.). Authors noted color changes with increasing Ag particles’ concentration [41]. Nevertheless, the main disadvantage of *n*-Ag composite is its low esthetic effect because it absorbs the color of the seal. Therefore, researchers have tried to achieve an antibacterial effect through *n*-ZnO. A composite consisting of different concentrations of *n*-ZnO was prepared and placed in a Heliomolar flow (Ivoclar Vivodent AG, FL-9494 Shaan/Liechtenstein). There are differences between *n*-ZnO and *n*-Ag composites which affect their activities. *n*-Ag materials release ions that work in a surrounding environment, but water-insoluble *n*-ZnO exerts its effect only on the composite’s surfaces. *n*-ZnO, as a surface bacterial inhibitor, prevents plaque growth, which is essential in caries treatment. The newly designed *n*-ZnO composite demonstrated strong antibacterial effect against *S. mutants*. It was noted that increasing the content of *n*-ZnO from 1 up to 5 wt.% did not significantly affect its activity. Percentage of conversion degree also did not differ significantly within the group studied and reached a ca. value of 50%. Flexural strength and modulus revealed no differences within the group, with values of ca. 80 MPa and ca. 3.5 GPa, respectively. Comprehensive strength increased with up to 1 wt.% of *n*-ZnO (ca. 170 MPa), but it dramatically dropped with increasing amount of nanoparticles, however, modulus remained at the same level (ca. 250 GPa). Obtained results suggest that *n*-ZnO as a nanofill in commercially available dental composite retains its mechanical properties at the same level as unfilled reference and enables significant antibacterial activity [42]. It is well known that fluorides inhibit bacteria growth and improve re-mineralization of tooth structures [34]. A new composite with nanofillers comprising a combination of fluoride and hydroxyapatite has been developed. In the beginning, fluoridated hydroxyapatite (FHA) with nanorod morphology was synthesized via a hydrothermal process. Secondly, the obtained nanofiller was incorporated into a composite matrix containing *bis*-GMA and TEGDMA. Mechanical profile of the newly designed dental restorative increased in comparison with the control and revealed the highest values of flexural strength and modulus of 100 MPa and 2.5 GPa, respectively, for the composite with 0.2 wt.% FHA content. However, the immersion test has indicated that the release of fluoride is possible and after 21 days of immersion it reaches a concentration of ca. 1 ppm, which is enough to inhibit enamel demineralization [43,44,45,46].

New dentistry restorative materials in particular composites need extensive studies to improve their mechanical properties such as flexural strength, modulus and compressive strength (Table 1). The greatest problem in using composite is polymerization shrinkage, which causes tensions in healthy tooth tissues, resulting in pain symptoms. To improve the mechanical properties, fillers or most recently nanofillers have been introduced into the composite matrix. Tests with clay as a nanofiller have been performed. *bis*-GMA and TEGDMA were used as the main ingredients of the matrix. The filler—clay substance—is an inorganic material with a nanofiber shape—Ca_x_(Al_2−x_Mg_x_(OH)_2_Si_4_O_10_—called Fuller’s Earth (FE). During polymerization, the clay was put into the weak regions of the composite. Mechanical investigations showed that for 0.72 wt.% of nanofiller, the properties were the best, with a flexural strength of 105 MPa, flexural modulus of 2.79 GPa and a work-of-fracture (WOF) 8.19 kJ/m^2^, which is very important for cervical restorations. In comparison with the composite without filler, these values slightly increased [47]. However, clay substances can insignificantly affect some mechanical properties of the dental composite, as described by Mucci et al. The authors introduced a polymer montmorillonite (MMT), which is composed of sheets of approximately 1 nm thickness and a diameter in the range of 50 nm to a few µm. In this study, MMT was mixed with methacrylate monomer (TEGDMA) and then polymerization reaction with 2,2-*bis* [4-(2-methacryloxyethoxy)phenyl]propane (*bis*-EMA) was initialized. Mechanical characteristics of the obtained seal were prepared. The results did not show any improvement in the measured factors. Additionally, the nano-clay composite revealed higher water sorption than the unfilled one of about 10–15% [48]. Water absorption softened the dental material and predisposed it to abrasive wear and staining [34]. Other substances such as nanosized silica were taken into consideration to improve the composite characteristics. Wang et al. have prepared a hybrid composite, the porous diatomite and Aerosil OX-50 particles with ca. 40 nm diameter, by introducing it into a composite matrix consisting of *bis*-GMA and TEGDMA. The best mechanical profile was obtained for 70% content filler in the composite with a mass diatomite to nano-silica ratio of 21:49. The flexure strength, elastic modulus, Vicker’s microhardness and compressive strength reached values of ca. 130 MPa, 7 GPa, 120 HV and 190 MPa, respectively. Interestingly, significantly lower values were obtained for fillers consisting only of nano-silica. Authors have explained this phenomenon by filling the spaces between cylinders and plates of diatomite in composite with nanosized silica [49]. Even though introducing nanofiller into a composite enhances its mechanical profile, there are still problems to address, such as lack of proper dispersion because of a high surface area and a tendency to form agglomerates. To tackle this, Atai et al. have proposed the formation of secondary particles using nano-silica through thermal sintering. The hypothesis was that secondary particles will preserve their properties and will be better dispersed in the composite. The new composite achieved a higher value of flexural strength than the microfilled one and it is similar to Filtek Supreme^®^ (ca. 120 MPa), whereas the flexural modulus was noted as the best for the sintered nano-silica composite. Interestingly, the best surface roughness profile was achieved by the newly designed composite, which is directly linked with particle dispersion. Better dispersion and lower surface roughness enable to achieve the appropriate esthetic effect [50]. Extensive studies on the usability of nano-silica in the dental composite also include assessment of cytotoxic properties of the newly designed composites. Researchers have prepared a nano-silica composite based on an alkoxy-ethyl-CA matrix. They concluded that the high mass percentage of nano-silica particles in the composite improves the viability of L929 mouse fibroblasts. It turned out that with increasing amount of nanoparticles, the release of formaldehyde decreased [51]. To obtain an ideal dental seal based on composites, new nanoparticles are being studied. An interesting approach was presented by Zhang et al. They have implemented silane-modified single-walled carbon nanotubes (SWCNTs) into a composite matrix of urethane dimethacrylate (UEDMA, Durafill, Heraeus-Kulzer, Germany). It is well known that the mechanical properties of SWCNTs are much better than steel. Firstly, SWCNTs were coated with nano-SiO_2_, then modified with allytriethoxysilane (ATES). In the second step, the prepared nanoparticles were introduced into the matrix. The obtained nanofilled composite revealed high mechanical performance with a flexural strength (ca. 142 MPa) significantly higher than the reference [52]. Silva et al. tried to use a similar shape of filler in the dental seal material. They used cellulose microfibers and cellulose nanocrystals as fillers, whereas glass ionomer cement was used as a matrix. Cellulose microfibers, with a similar shape to SWCNTs, slightly improved the mechanical properties in comparison to GIC without a filler. Considerable improvement in all the studied mechanical properties was observed for cellulose nanocrystals. The conclusion from these studies is that fiber-shaped fillers improve the mechanical profile of dental material, but the size of filler particles plays a significant role [53]. Introducing nanoparticles into dental composite enables to improve the mechanical properties, but on the other hand, it can change other parameters such as color. The color of the dental seal is the most important aspect from an esthetic point of view. The seal should not visually differ from the natural tooth, especially when contact surfaces are placed on a visible site. Yu et al. have performed studies on the impact of fillers on composite color. Matrix containing BisGMA and TEGDMA and different ratios of nano-silica filler and micro-sized glass filler were prepared. The composites were immersed in water for 72 h and then subjected to thermocycling (5000 cycles) at 5–55 °C. The color was measured before and after immersion. The results indicate the stabilization effect of nanofillers on color, especially for the composite filled only with nanoparticles. Interestingly, microfiller was the weakest color stabilizer within the studied group [54]. On the other hand, nanofillers can modify not only the mechanical properties but also the color. An example of nanoparticles that can affect color is titanium dioxide particles (TiO_2_). It was proven that TiO_2_ particles of size in the range 300–400 nm impact seal color [55].

The biocompatibility and toxicity of dental restorative materials play a role in the amount of eluted substances. The eluted mixture consists of some unpolymerized starting material, such as monomers, initiators of the polymerization reaction, filler and others. Toxicity tests with methacrylate monomers, such as *bis*-GMA, TEGDMA, 2-hydroxyethyl methacrylate (HEMA) and methyl methacrylate (MMA), were performed. The in vitro evaluation was prepared using human gingival fibroblasts as a cell model. The results showed no toxicity of TEGDMA, HEMA and MMA at concentrations up to 10 mM. *bis*-GMA turned out to be toxic at the concentration of 0.25 mM [56]. Thus, Durner et al. have prepared a composite containing silver nanoparticles using Tetric flow^®^ (Ivoclar Vivadent, Ellwangen, Germany) as a matrix. The authors have studied the dependency between composite release profiles and the increasing amount of silver nanoparticles. It was observed that release of monomers increased with a higher amount of nanofillers. The authors have explained this phenomenon by increasing unpolymerized monomers and associated the decrease in polymerization yield with increasing nanoparticles concentration caused by the reflection, scattering, and absorption of photons by silver particles. Additionally, they have indicated the possibility of nanosilver’s reaction with the photoinitiator, resulting in complexes formation [57]. The same research team has published studies on the correlation between the degree of conversion (polymerization reaction yield) and eluting profile for nano-hybrid materials Venus^®^ Diamond (Heraeus Kulzer, Hanau, Germany), Tetric Evo Ceram^®^ (Ivoclar Vivadent, Ellwangen, Germany) and Filtek™ Supreme XTE (3M ESPE, Seefeld, Germany). The degree of conversion was measured using the FT-IR method, which enables to detect and quantify the C=C bond. Authors have indicated a strong inverse dependency between the degree of conversion (DC) and the quantity of eluted substances for all dental restorative materials studied. The impact of time on the release of composite ingredients was also measured. Authors have proven that in the irradiation time of 20–40 s, there are no significant concentration or composition differences of eluting substances [58].

Thanks to nanofillers, dental restorative materials turn out to be more wear resistant. Wear is the greatest problem in composite restorative materials. Mayworm et al. have confirmed high resistance of nanocomposites to abrasive wear. Scientists have studied two nano-hybrid commercial materials’ susceptibility to wear (FiltekSupreme^®^—3M ESPE, Seefeld, Germany and Esthet X^®^—Dentsply, Caulk, Ann Arbor, MI, USA). The results have demonstrated that the surface hardness after immersion in artificial saliva decreased. Authors have associated this phenomenon with filler size. It can be noted that removing large filler particles from a composite is easier than removing the smaller ones and this is connected with decreasing mechanical properties. The large fillers possess low surface area to volume ratio, which is connected with a weaker interaction between fillers and polymer net. Mayworm et al. have noted the above-mentioned dependency within the studied nano-hybrid composites group. According to the manufacturers, the studied materials exhibit similar filler content—ca. 60%. In the SEM images, Filltek Supreme^®^ showed larger fillers than Esthet X^®^, which the authors have linked with a greater decrease in wear and a lower decrease in microhardness for Esthet X^®^ [59]. In order to check the wear resistance of nanofilled materials upon brushing, in vivo studies using simulated conditions were performed. The toothbrush abrasion device simulated teeth cleaning and dark acidic specimens simulated food. The study included four groups consisting of 20 teeth. The first was an untreated control group; the second was treated with Pro Seal™ (Reliance, Reliance Orthodontic Products, Itasca, IL, USA), which is a fluoride-releasing sealant; the third was treated with Light Bond™ Filled Sealant (Reliance, Reliance Orthodontic Products, Itasca, IL, USA), which is a microfilled composite; and the fourth was treated with Seal & Protect (Dentsply, De Trey GmbH, Konstanz, Germany), which is nanofilled dental material. The results showed that Seal & Protect revealed the highest resistance to abrasion, which was linked to the size of the composite filler [60]. Interestingly, different shapes of filler exert a significant effect on wear resistance. Turssi et al. have studied the wear resistance of twelve experimental dental composites, the matrix of which consists of *bis*-GMA, UDMA and TEGDMA. Authors have used different combinations of materials with various shapes (spherical and irregular) and sizes (from 100 nm up to 1500 nm) as fillers. Each formulation had 56.7 vol. % of filler. Authors have concluded that both size and shape play a crucial role in the wear resistance of a dental composite. Irregular-shaped particles seem to be more resistant to wear than the spherical ones. They have suggested that irregular shapes determine greater surface for adhesion. The highest durability was observed in composites with filler of smaller particle size; the best composite contained a filler of ca. 100 nm [61].

It should be remembered that dental personnel working with composite materials can come into contact with the nanoparticles. It is possible that during shaping (polishing or grinding) of the seal, nanodust can be inhaled through the respiratory tract. Many research teams have noted toxicity of nanoparticles, which depends on the character of particles, shape, size and many other factors [62]. In light of this knowledge, Van Landuyt et al. have performed studies exploring the answer to the question: do polishing or grinding generate nanodust? The authors have built, what is called a Plexiglas box, where the polished composite was studied. Additionally, the box was equipped with IOM-inhalable dust sampler, which supplied dust for further investigations using the TEM technique. Commercially available composites—one hybrid, one micro-hybrid, one nanofilled and four nano-hybrid—were subjected to the studies. The obtained results indicate that the released dust particles were of sizes smaller than 500 nm and particles of size below 100 nm were also determined. In order to avoid the influence of nanodust on dental personnel’s health, the authors suggest using water cooling upon polishing of composite material and wearing masks with filter adjusted for small particles [63].

## 4. Adhesives

The dental adhesive system has been introduced into dental practice for better seal adhesion to the tooth structure, especially dentine. Seal adhesion is considered as an important parameter in restorative dentistry, which determines sealing durability. The adhesive system usually consists of three main ingredients: etchants, primer and adhesive. The etchant dissolves the mineral surface simultaneously forming the demineralized collagen network of dentine, allowing the penetration of primer into the tooth structure. Strong acids of pH = 1–2 are used as etchants. The role of the primer is the formation of a bifunctional layer, which connects the hydrophilic tooth structures and the hydrophobic adhesive. The structure of the primer could be considered as follows: methacrylate group, spacer group, and reactive group. The methacrylate group is connected to the composites, the spacer group provides flexibility to the linker, and finally, the reactive group forms a strong bond with dentine. The adhesive is a monomer, which, under curing light, connects to primer, filling the interfibrillar space of the collagen network. The adhesive forms a hybrid layer as a basement for the composite seal. Etch-and-rinse systems requiring 2–3 steps or self-etch (in 1 step) can be distinguished [34,64].

**Figure 4:**
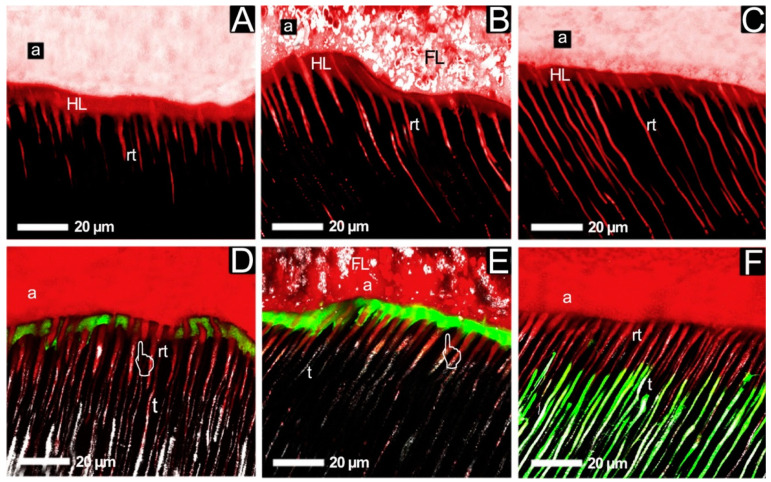
Single confocal images of the resin–dentin interfaces created using the SB control and SB doped with ZnO or ZnCl_2_ after 24 h of artificial body fluid storage; (**A**) reference SB, (**B**) SB modified using nano-ZnO particles equipped with reflective filler, (**C**) SB modified with ZnCl_2_, (**D**) SB reference with fluorescein nanoleakage (pointed by finger), (**E**) SB modified using nano-ZnO with fluorescein nanoleakage (pointed by finger), and (**F**) SB modified using ZnCl_2_ with no fluorescein nanoleakage. The abbreviations present on image: a—adhesive, rt—resin tags, t—dentinal tubules, FL—reflective filler, HL—hybrid layer [68]. Reprinted with permission from Toledano et al., Dental Materials; published by Elsevier, 2013.

Recently, Calvo et al. have studied the bond stability of three commercial glass ionomer cements. Glass ionomer cements are known to acts as self-adhesives to the tooth structures including caries-affected tissues. The following materials were applied: Ketac™ Molar Easymix (3M ESPE, Seefeld, Germany), Vitremer (3M ESPE, Seefeld, Germany) and Katec™ Nano Light-Curing (3M ESPE, Seefeld, Germany) for tests. Authors have chosen the most common aging method—immersion in water. Microtensile bond strength (µTBS) was measured immediately and after 2 years of storage in the water. The highest bond strength was revealed for Ketac™ Nano just after curing and Vitremer after 2 years of storage. Authors have associated these obtained results with the bonding mechanism. Attachment of glass ionomer cements results from the formation of a chemical bond between polycarboxylic acids and hydroxyapatite. The resin modifies the glass ionomer cement bonding to the tooth structures via chemical and micromechanical adhesion, which causes a high bond strength just after curing. Otherwise, fillers are released during the storage of cement in water for 2 years. This phenomenon leads to diminished micromechanical interactions. It should be remembered that introduction of a filler into composite lowers the polycarboxylate content (lower amount of chemical bonds). This situation is linked with weaker adhesion. Removing fillers of greater particle size results in significant loss of mechanical adhesion; simultaneously, such fillers possess low surface to volume ratio, which causes a decrease in the quantity of monomers bound to dentine. Thus, nanofiller releasing from a nanofilled glass ionomer leads to smaller disruption in adhesion strength. Nanofiller possesses high surface to volume ratio, which allows for its introduction into cement with a higher quantity of binding material to the tooth monomers [65]. One of the very important issues is to fully remove the caries from the tooth, which is usually not achieved. This mentioned fact leads to secondary caries, caused by residual bacteria, in restorative dentistry. Therefore, the researchers proposed the incorporation of an antibacterial agent into a restorative primer. The primer is directly linked with dentine and builds an interface between the tooth structures and the seal. Previously, 12-methacryloyloxydodecylpyridinium bromide (MDPB) was implemented into a primer and an antibacterial activity was observed. In order to enhance the antibacterial activity, Zhang et al. have introduced nano silver particles (*n*-Ag) and MDPB into a primer. Commercially available Scotchbond Multi-Purpose (3M, St. Paul, MN, USA; SBMP) was used as the primer. Antibacterial studies were performed and they revealed that this primer (MDPB + *n*-Ag) was the strongest inhibitor of bacterial growth (*Streptococcus* spp. including *S. mutans*) within the studied group. Interestingly, the primer equipped with only *n*-Ag caused significantly lower antibacterial activity than the primer with MDPB. Therefore, this can be observed as a synergistic mechanism of action. Additionally, quantity of lactic acid formation (metabolite of the bacteria) was tested and results showed a that the amount decreased the most with primer-*n*-Ag, followed by primer-MDPB and primer-*n*-Ag-MDPB, which is the same as the antibacterial effect. Authors have also tested bond strength and have concluded that there is no impact on bond strength. All the studied primers exhibited a bond strength of value ca. 30 MPa. The presented modified primer showed no toxic effect on human gingival fibroblasts in the performed cytotoxicity assay [66]. Re-mineralization process between dentine and composite is very important on the contact surface. In order to achieve a high curing potential, nanoamorphous calcium phosphate (NACP) was introduced into the primer and the adhesive material. It was previously proven that NACP can release a substantial amount of Ca and P, especially in cariogenic pH = 4 [39]. Additionally, these materials have been supplied with *n*-Ag for antibacterial treatment. Scotchbond multi-purpose (3M, ESPE, St. Paul, MN, USA) was used as a matrix. The experiments revealed that the introduction of 0.1% *n*-Ag and up to 40% NACP does not impact the dental bond strength in comparison to the reference (pure Scotch bond multi-purpose). Moreover, the newly designed adhesive system achieved a high antibacterial potential. A ca. 10-fold decrease in the total CFU microorganism and 5-fold decrease in the CFU of *S. mutans* as compared to the reference material were noted. Authors have noticed a decrease in the metabolic activity and a decrease in lactic acid production [67]. It is well known that the seal toughness depends on adhesion strength to dental structures. Adhesive systems should be integrated well with dentine to create a layer that will enable composite bonding. An adhesive containing ZnO nanoparticles and ZnCl_2_ was obtained to improve its integrity with dentine. It was a modified commercial adhesive system, Adoper™ Single Bond Plus (3M ESPE, St. Paul, MN, USA—SB). The results have indicated a high stability of bond strength of the modified SB after storage in a simulated body fluids solution for 24 h in comparison with the control. Measurements were performed after 3 months of storage [68]. Interestingly, the adhesive system doped with *n*-ZnO particles revealed an increased hardness. Authors have associated this phenomenon with earlier reported ability to replace calcium ions with zinc ions in the apatite crystals [69]. Figure 4 presents an interface dentine—adhesive system images from a study performed by Toledano et al. It can be concluded that nanofilled adhesives (Figure 4B,C,E,F) integrate well with dental tubules in contrast to the reference. Nanoleakage test with fluorescein was performed, and it showed that nano-ZnO highly reduces nanoleakage when ZnCl_2_-filled adhesives revealed no leakage [70].

In order to improve bond strength, scientists have focused on other candidates for nanofillers to be used in dental adhesives. Lohbauer et al. have obtained zirconia nanoparticles (ZrO_2_) via laser vaporization. The employed technique enables the formation of spherically shaped nanoparticles of ca. 20–50 nm size, which allows to avoid the agglomerate formation. Reduced agglomeration tendency for spherically shaped particles is connected with only one point of Van der Waals interaction between particles. The particles need less energy to interrupt the aforementioned interactions. The authors studied the microtensile bond strength (µTBS) of the commercial adhesive system—Adper Scotchbond Multi-Purpose (3M ESPE, St. Paul, MN—SBMP)—with the addition of zirconia nanofiller into the primer or the adhesive solution. It was noted that µTBS improved with increasing nanofiller concentration up to 20 %wt. Moreover, for 20 %wt of nanofiller in the adhesive solution, a significant difference in µTBS, with an advantage for the nanofilled primer (41 MPa for modified primer and 32 MPa for modified adhesive solution), was noted. Based on TEM micrographs, it has been concluded that zirconia fillers added to the primer accumulate on top of the surface, otherwise addition to adhesive solution resulted in dispersion. µTBS was improved through reinforcement of the interface surface of the bond system [70]. The same research team has recently published data regarding the influence of SiO_2_-BaO glass filler of size 180/500 nm with an irregular shape on the mechanical properties of a bond system. In comparison to an unfilled bond, increase in tensile strength, elastic modulus and µTBS up to 10 %wt. of the nanofiller were observed. Additionally, the agglomeration of nanofillers was noted, which is connected with the irregular shape as mentioned above [71].

The dental pulp exposure in most cases ends with an endodontic treatment. A restorative material protecting the pulp in such a situation has not been developed yet. Yoshida et al. proposed an adhesive material for direct pulp capping. The material was based on Super-bond (SB, Sun Medical Co., Ltd., Shiga, Japan), in which nano hydroxyapatite was incorporated. The material with 30% of nano-component was the most promising. Experiments were performed on a rat pulp exposure model and showed that the studied material caused reconstruction of the dentin with dentin tubules [72]. Nevertheless, results presented by Yoshida et al. drew attention to the fact that the major obstacle to full recovery is pulp contamination with bacteria.

## 5. Endodontics

Endodontics is focused on the treatment of dental pulp disorders. The most important aspect of endodontic treatment is mainly the eradication of bacteria, and viruses and fungi as well, all of which are an origin of endodontic diseases [73]. Infection of dental root canals can be initiated in three ways. First, bacteria penetrates the dentine via healthy dental structures—dental tubules. The diameter of a dentin tubule ranges from 0.2 to 0.9 µm, which is suitable for a bacteria cell. It is important to mention that infection via dental tubules is easier in a non-vital tooth in comparison to a vital one. Dentinal fluid flows through the tubules of vital tooth and it hampers bacteria movements into the pulp. The second infection tract is the direct pulp exposure caused by caries—most common or tooth injuries. The third infection pathway is the anachoresis process—bacteria is supplied with blood and/or lymph through vessels into the apical region of a damaged root. Treatment of pulp disorders proceeds as follows: removing infected pulp structures, shaping root canals for filling, cleaning and decontamination and finally, restoration [73,74]. Among the many bacteria infecting root canals (*Actinomyces naeslundii*, *Aggregatibacter actinomycetemcomitans*, and *Fusobacterium nucleatum*), *Enterococcus faecalis* is the primary cause of dental pulp infections; as a facultative anaerobe, it can persist in unfavorable conditions, especially when it penetrates the dental tubules. The mentioned bacteria is a major obstacle in successful endodontic treatment [75,76]. A commonly used bactericidal agent in dental practice is sodium hypochlorite (NaClO) in concentrations of 4–5% [77]. It was proven that dental tubules can be penetrated by bacteria up to 300 µm inside, whereas clinically used NaClO can penetrate tubules up to 130 µm, which can be the reason for treatment failure and the surviving bacteria in tubules can initiate the infection process once again [78,79]. It should be mentioned that *E. faecalis* exhibits resistance to *β*-lactams (cefalosporines), clindamycin, tetracyclines, macrolides, and glycopeptides (vancomycin) [80,81,82]. Nanostructures can be used in endodontics mostly as antibacterial agents and as nanofillers enhancing the properties of endodontic seals.

In light of the above data, scientists have focused on looking for alternative antibacterial agents in nanoscience. Thus, the usability of photodynamic antimicrobial chemotherapy (PACT) in decontamination of root canals was assessed. Among others, our group has performed studies on development of PSs against *E. faecalis*. At a high activity during the inactivation of this bacteria at the level of above 5 logs, reduction in bacterial growth was reported. Tested compounds belong to the porphyrinoid group, i.e., porphyrazines, phthalocyanines, chlorins, etc. Simultaneously, other researchers reported high potential of PACT in inactivating a wide range of microbe species. Thus, all these promising results obtained in the inactivation of some bacterial species have inspired scientists and manufacturers to develop PACT-based bactericidal systems [83,84,85,86,87,88,89,90,91,92,93,94,95,96,97,98,99]. Antibacterial systems based on PACT, such as PAD™ (Denfotex, London, UK) and Helbo^®^ (HELBO Photodynamic Systems GmbH, Wels, Austria), have been introduced into dental practice. According to manufacturer’s specification, PAD™ and Helbo system^®^ as PS contain toluidine blue and methylene blue. Meire et al. have compared the activity of the mentioned PACT protocols using the NaOCl flushing procedure for growing *E. faecalis* biofilm. Obtained results have indicated that PACT systems have a weak activity against *E. faecalis* in biofilms (ca. 2 log), while NaOCl possesses high potential with >6 log reduction in bacteria viability or decontamination method with laser Er:YAG pulsed irradiation (4.3 log) [21]. PACT has become an adjuvant therapy for root canal disinfection; on the other hand, this method is easy, fast and promising. Thus, scientists are focused on looking for a new PS of higher activity. High potential of PACT as an adjuvant procedure in endodontics has been concluded based on the studies performed on primary molars. To achieve proper occlusion of the secondary teeth, the primary ones should stay in the arch as spacer for the secondary teeth. Therefore, endodontic treatment of deciduous teeth is very important [100]. Pinheiro et al. divided twenty primary molars into two groups, one of them proceeded with a manual instrumentation technique and the second with a rotary one. Three different PACT procedures were performed, each on 10 root canals. Toluidine blue O and laser; fuchsin and halogen light; and fuchsin with LED as light sources were applied in the three procedures. All the PACT techniques demonstrated a significant ability to reduce *E. faecalis* in the CFU units of the root canals. Additionally, the authors have concluded that there are no differences in bacteria decontamination between routine root canals treatment techniques and the adjuvant PACT procedures [101]. Similar studies were performed by the same authors on primary teeth using a PS toluidine blue O combined with a low-intensity laser. The obtained results have indicated that instrumentation enabled to remove ca. 82% of viable bacteria. Interestingly, using an adjuvant treatment PACT with toluidine blue O leads to microbial reduction up to ca. 98% [102]. Moreover, there is a case report on successful endodontic treatment of a 4-year-old female patient. This treatment used methylene blue as a PS at a dose of 50 µg/mL and irradiation during 5 min with a light dose of 40 J/cm^2^ [103]. The high efficacy of PACT in deciduous teeth root canal disinfection was confirmed by de Sant’Anna in a case report of successful endodontic treatment. A five-year-old patient with diabetes mellitus (type I) after incisor injury was treated with methylene blue at a dose of 50 µg/mL and a light density of 40 J/cm^2^ [104]. The main disadvantage associated with PACT is staining of the tooth structure. The staining process is caused by PS, which usually is a member of a dye family compounds, i.e., methylene blue, phthalocyanine derivatives and others. Thus, development of discoloration seems to be one of the interesting phenomena in endodontic PACT. Therefore, studies have been performed on forty canal roots, which have been treated using PACT with methylene blue as a PS. After PACT treatment, three flushing protocols and control (flushing with saline) were used. Firstly, usability of 2.5% sodium hypochlorite (NaOCl) was assessed, secondly 2.5% NaOCl with Endo-PTC cream (10% urea peroxide, 15% Tween-80—detergent, and 75% carbowax as vehicle) and finally the third flushing protocol with 75% ethyl alcohol was tested. Photographs were taken before PACT, after PACT and after flushing. Obtained images were compared via Adobe Photoshop^®^. Authors have concluded that the best discoloration protocol is the combination of NaOCl with Endo-PTC cream, which enables continuous oxygen release, probably responsible for discoloration [105]. In dentistry, methylene blue and toluidine blue with strong blue color are commonly used as PSs. Additionally, other classes of PS reveal a strong color. These mentioned dyes are the cause of temporary staining of soft tissues in periodontology and bone structure in endodontics, because of diffusion into dental tubules. In light of the above data, the decolorization procedures were developed. Späth et al. have studied a new class of PS of natural origin, which possesses a color similar to the natural teeth. This PS was phenalen-1-one derivatives, irradiated with blue light which strongly generate singlet oxygen, the main active factor killing bacteria. The studies performed on them indicated that a new quaternary phenalene compound efficiently inactivates bacterial strains of *E. faecalis* with a magnitude over 5 log [106]. Tennert et al. have performed antimicrobial tests on *E. faecalis* infections of primary and secondary endodontic treatment model. For these studies, 160 premolar and front teeth were chosen. The root canals of the first group of teeth were infected with *E. faecalis* and then treated with three different procedures: (1) flushing with 3% NaOCl, (2) PACT with toluidine blue and irradiation at 635 nm and (3) flushing (3% NaOCl) followed by the PACT procedure. The second group was also infected with the mentioned bacteria and then flushed with 3% NaOCl for 10 min and root canals were filled. Then, re-endodontic treatment was performed, filling material was removed, and three antibacterial procedures described above were performed. Authors have concluded that the combined treatment of (PACT + NaOCl) in primary infection reveals a higher potential in disinfection of root canals. Interestingly, for the secondary endodontic treatment, the best option was flushing with NaOCl solution [107].

**Figure 5:**
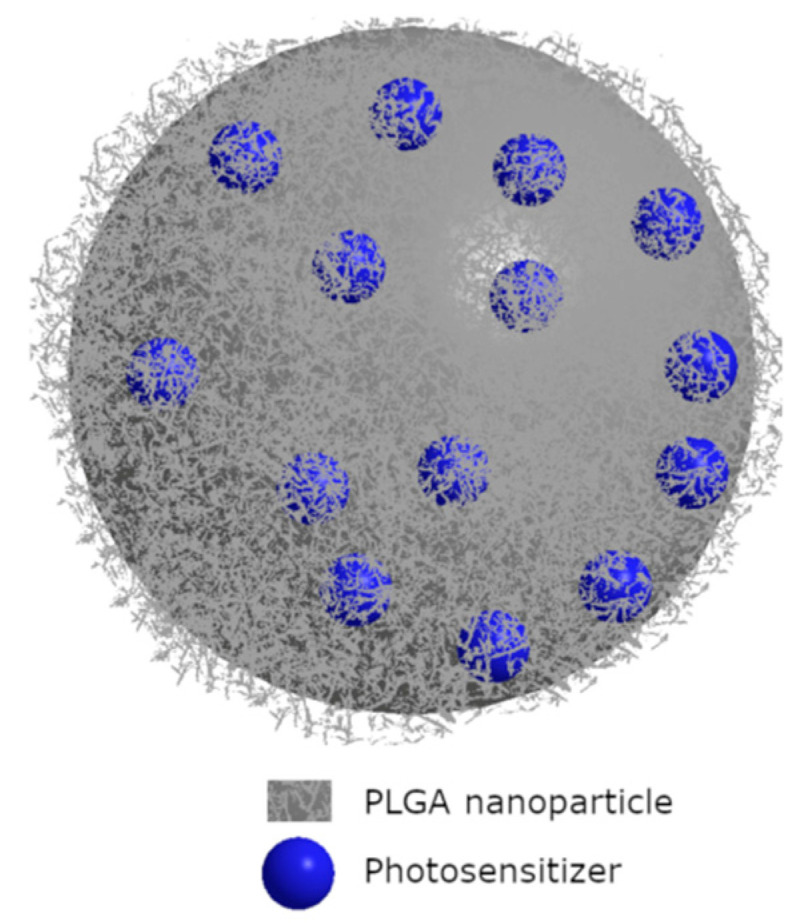
Copolymer of lactic and glycolic acids with methylene blue as a PS.

It has been proven that PDT efficacy against *E. faecalis* depends both on PS used and irradiation time. A trial was performed on eighty single-root human teeth with curcumin as a PS. Firstly, root canals were sterilized, then canal preparation was performed. Secondly, canals were infected with bacteria for 21 days. After this, the authors used several PDT protocols. The obtained data enable to conclude that 5 min LED irradiation time showed a significant decrease in bacterial viability. It should be mentioned that after 7 days of PACT procedures, bacterial growth was noted for all the samples. Even for the group, which showed the highest potential (curcumin + 5 min LED), growth in bacterial viability was observed [108]. Muhammad et al. have compared the activity of the three protocols in bacterial disinfection. They have tested Aseptim Plus^®^, a clinically used system in photodynamic disinfection of root canals. Activity of Aseptim Plus^®^ is based on toluidine blue and LED light of λ = 635 nm with a plastic tip enabling light delivery into the root canal. Additionally, the authors have investigated toluidine blue with laser light of λ = 650 nm equipped with an optic fiber. As a control, ultrasonic irrigation with 17% EDTA and 2.6% NaOCl solutions was used. Experiments were performed on thirty-four canal roots obtained from fifty extracted human teeth. Bacterial suspension for root canal infection was prepared with the following concentrations: 5% *Streptococcus salivarius*, 21% *E. faecalis*, 37% *Prevotella intermedia*, and 37% *Porphyromonas gingivalis*. The obtained results indicated that the ultrasonic irrigation revealed much more efficiency than any other method studied [109]. The new approach in treating *E. faecalis* disinfection was presented by Kranz et al., who have used clinically approved PS in head and neck cancers treatment. Authors have studied the activity of Foscan^®^ (mTHPC) in liposomal formulation known as Foslipos on bacteria in suspension. Obtained data enable to conclude that Foslipos completely inactivates bacteria in a concentration of 50 µm and a light fluence of 100 J/cm^2^. Application of 25 J/cm^2^ light at the same PS concentration has achieved a 6 log reduction in bacterial growth. Interestingly, incubation of 15 min without irradiation caused an 1.5 log bacterial reduction [110]. Further, the same group has published additional data concerning the bactericidal activity of mTHPC. Authors have used a new nano-carrier agent known as invasome. The main difference between liposomes and invasomes is the higher flexibility of invasomes. The property is developed with adding new ingredients such as terpenes and/or ethanol to shape lipid membranes. Scientists have performed studies on freshly extracted human teeth root canals, which were infected with *E. faecalis* strain (DSMZ 20376). They analyzed how deeply bacteria can colonize the dental tubules. According to the mentioned scientific group, *E. faecalis* colonize dental tubules up to 300 µm. The used PACT agent (mTHPC) enables an effective bactericidal effect up to 300 µm, similar to chlorhexidine gel (1%) used as a reference. The strongest effect has been noted for mTHPC encapsulated in invasomes [111]. On the other hand, it was mentioned that *E. faecalis* can penetrate dental tubules up to 980 µm, which was confirmed with scanning electron microscope. Methylene blue used as a PS has enabled bacteria eradication up to 900 µm. About 97% reduction in bacterial viability was achieved [112]. Other nanocarrier systems for PS in bactericidal studies of PACT were also used. Pagonis et al. used a nanocarrier based on polyester copolymer of polylactic acid and polyglycolic acid known as PLGA nanoparticles (Figure 5). Authors have introduced methylene blue into the PLGA of diameter ca. 200 nm and have tested its activity on *E. faecalis* in suspension and in roots of extracted human teeth. TEM images have indicated that the nanoparticles were placed on the bacterial wall. The obtained reduction in bacterial viability equaled suspension to 2 log for suspension and for 1 log root canals [113]. Interestingly, the combination of MB with PLGA improves nanohardness in comparison to conventional disinfectant agents. It was also noted that the use of these nanostructures increases the modulus of elasticity of root dentin. It was suggested that increase in nanohardness and modulus of elasticity is caused by photodynamic collagen cross-linking. In the same study, more increased rate of the mentioned parameters for nanostructures based on chitosan and Rose Bengal was observed. In such structures, a few possible pathways are considered, including internalization of chitosan into collagen of a dental root by photodynamic oxidation of amino groups [114].

**Figure 6:**
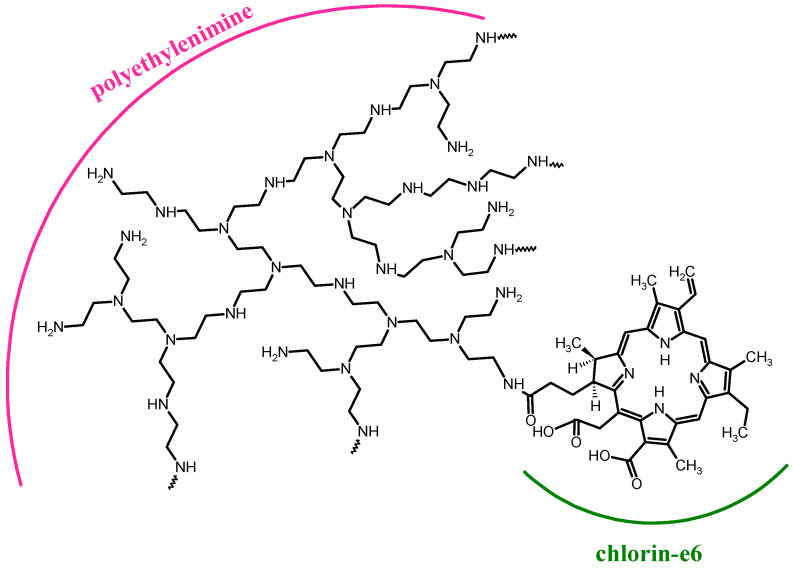
Structure of a PEI—chlorin e6 conjugate.

For PDT, the most important factor, next to a PS, is light delivery. The root canals are relatively long and differently shaped (i.e., curved apical region). Thus, Sabino et al. have tested two light delivery systems on a bioluminescent strain of *Candida albicans* biofilm in extracted teeth. Methylene blue at a dose of 90 µM as PS and λ = 660 nm for irradiation were used by the authors in two approaches: firstly, the laser tip and secondly the optical diffuser (cone-shaped) fiber. Diffuser enables to spread the light beam in all irradiated volumes. Efficacy of the light delivery was measured with a CCD camera. At the beginning of the experiment, root canals were infected with *C. albicans*, then PS was applied and irradiation was performed. After treatment, occurrence of a bioluminescence phenomenon was checked using a CCD camera. In conclusion, they have observed that light distribution over the root canals is dependent on the light delivery system used. The optical diffuser fiber system was more efficient, for which the log reduction in *C. albicans* growth was 3 compared to 1 for the laser tip system [115]. Similar studies were performed by Garcez et al. wherein five conditions were tested. In the first approach, roots were irradiated by laser supplied with a laser tip (area 0.04 cm^2^); in the second experiment, roots were irradiated by laser supplied with a laser tip (area 0.028 cm^2^). The third approach was the same like the first one but the cured teeth with crowns were subjected to study. The curing of the fourth group was performed according to the methodology of the second one, but teeth with crown were cured instead of roots. The fifth group consisted of teeth with crowns irradiated by laser equipped with a fiber optic diffuser tip. PDT procedure was performed with methylene blue as a PS and *E. faecalis* as a model bacteria. For the first and second conditions (only roots), a 2 log reduction in bacterial growth was noted. Interestingly, the third and fourth groups (teeth with crowns) achieved one log reduction in bacterial growth. Finally, for the fifth condition (teeth with crowns), light delivery with a fiber optic diffuser tip was used, and the reduction in bacterial growth was 4 logs. Thus, they have concluded that uniform light distribution in root canal was achieved for light delivery with an optical fiber coupled with a diffuser [116]. The same scientific group applied bioluminescent Gram-negative bacteria strains of *Proteus mirabilis* and *Pseudomonas aeruginosa* to assess bactericidal efficacy of the polyethylenimine chlorin e6 (Figure 6) conjugate. Authors have carried out treatment on freshly extracted human single-rooted teeth. Conventional endodontic treatment and PACT were applied separately as well as combination of both methods. In conclusion, both the conventional procedure and PACT used alone were effective. Interestingly, the combination of both procedures revealed better efficacy in disinfection. Therefore, authors have recommended the PACT technique as an adjuvant treatment for endodontic infection [117].

Not only has the light delivery system has a great impact on PDT treatment, but it also impacts the source of light. Cheng et al. have described a PACT procedure with three light sources: Nd:YAG, Er:YAG, and Er, Cr:YAG lasers. In an experimental model, root canals in extracted human teeth were infected with *E. faecalis*. Then, PACT procedures with methylene blue as PS were performed with the mentioned laser systems. Authors have concluded that the procedure with Er:YAG laser as a source, flushing with NaClO, normal saline, and distilled water has revealed the highest potential within the groups studied. This developed protocol enables to eradicate bacteria inside dentin tubules up to 300 µm, which is a significant improvement in comparison to standard flushing with 5.25% NaClO [118]. Additional studies were performed and it turned out that LED light was not interfered by dentin of human teeth [119].

An interesting approach is to link the conventional treatment based on antibiotics with nanoparticles or bactericidal nanoparticles alone.

Ariaz-Moliz et al. have tested polymP-*n* Active NPs loaded with doxycycline against *E. faecalis* biofilm in the dentine. It was observed that even unloaded NPs can reduce biofilm’s bio-volume by interacting with extracellular polymeric substances. Functionalized NPs with doxycycline enabled the inhibition of biofilm growth. Moreover, the obtained nanoparticles may be used as the delivery platform for doxycycline, which is known for strongly binding to the calcium ions of dentine. What is most exciting the doxycycline can be agglomerated inside the root canal dentine and it forms an antibiotic reservoir [120,121,122,123]. However, in another study performed by Toledano et al., polymP-*n*-active NPs doped with Zn, Ca and doxycycline have been reported. The sealing efficacy and dentin mineralization were tested. It turned out that NPs doped with zinc revealed the best parameters, such as the lowest microleaks, the highest Young modulus and dentin mineralization [69]. Bulavinets et al. have proposed silver NPs for photothermal root canal disinfection. In this initial study, they have noticed only slight growth inhibition of *S. aureus* with NIR (880 nm) irradiation. Authors suggest that increase in NPs concentration may improve the activity [124]. On the other hand, Karczewski et al. have developed a nanofiber of ca. 350 nm length based on polydioxanone functionalized with clindamycin, metronidazole and ciprofloxacin. Newly obtained potentially endodontic nano-tool was evaluated for the purpose of bacteria inactivation (*Actinomyces naeslundii*, *E. faecalis*, *Aggregatibacter actinomycetemcomitans*, and *F. nucleatum*), impact on dentine coloration and dental pulp stem cell viability. All performed tests showed high antibacterial potential and no impact on cell viability. Moreover, no visible changes in dentine color were noted [125]. Baras et al. have proposed a new root canal sealer based on dimethylaminohexadecyl methacrylate (DMAHDM) containing silver NPs and amorphous calcium phosphate NPs (NACP). This developed nanosealer is dedicated to treat secondary root canal infections. It is well known that if DMAHDM comes in contact with bacteria, it can efficiently disrupt the functions of its membrane. Authors also implemented silver NPs, which are released inside the targeted area to kill bacteria in the whole volume of root canal. Reduction in *E. faecalis* biofilm growth of about 3 logs was achieved. What is important, the significant sealer parameters such as flow and thickness were not disturbed in comparison to pure DMAHDM. Authors additionally incorporated NACP to the sealer; NACP was released and it improved dentine microhardness. Before sealing, root canals are treated with aggressive disinfectants such as NaOCl, which, as a side effect, causes dentine demineralization. NACP that is released from seal within 1 month re-mineralizes the dentin and improves its microhardness [126]. Another approach of developing DMAHDM functionalized with silver NPs was presented by Seung et al. They have used 2.5% of DMAHDM and 0.15% Ag NPs (like Baras et al.) and mixed them with clinically approved root sealer epoxy resin—AH Plus^®^. Authors have noted deterioration of physical parameters such as flow, setting time, and dimensional time. Nevertheless, all parameters still meet the ANSI/ADA specifications. Importantly, after 1 day, the modified AH Plus showed the highest inhibition of *E. faecalis* growth, whereas after 7 days its antibacterial activity was ca. 2-fold stronger in comparison to AH Plus^®^, which lost its antibacterial activity [127]. Graphene oxide (GO) has been also proposed for root canal disinfection. Ioannidis et al. modified GO with silver NPs. GO, known for its bi-dimensional structure, may cut bacterial membrane leading to leakage of bacterial cell in the outside. The obtained material in the presence of ultrasounds yielded the same reduction in bacterial biofilm growth like the 2.5% solution of NaOCl, which caused a strong demineralization. It is suspected that ultrasounds improve silver NPs’ release [128,129,130].

Nanostructures have been introduced into endodontic sealers as bactericidal, radiopacifier agents and calcium ion source.

Beyth et al. have tried to develop an endodontic nanosealer with bactericidal activity. Therefore, quaternary ammonium polyethylenimine was introduced into an epoxy-based sealer. Influence of a new nanosealer containing 0%, 1.5% and 2.0% (wt./wt.) nanoparticles on bacterial growth was studied. Obtained results indicated that nanoparticles of 1.5% quantity provide complete inhibition of bacterial viability. It should be mentioned that the best activity was observed at pH < 4 and pH > 6. Authors have associated this phenomenon with the activity of H^+^ ions instead of nanoparticles in a pH range below 4. Otherwise, in a pH range of 4–6, isoelectric point is achieved and electric attractions between particles and bacterial membrane are weaker than at pH > 6. Moreover, cytotoxicity assay was performed and it indicated no cytotoxicity of the new endodontic sealer. Authors have also designed studies to analyze the physical properties of solubility, thermal analysis, and flow assay, which suggest that the introduction of nanoparticles does not significantly influence these sealer parameters [131]. Nowadays, mineral trioxide aggregate is used in dental practice for root perforation repair. It consists of Portland cement and bismuth oxide as a radiopacifier. Unfortunately, the bismuth oxide increases porosity and solubility of the cement, lowering its resistance. Scientists have looked for a new radiopacifier with better characteristics. Zirconium oxide and niobium oxide seem ideal candidates for being used as a radiopacifier. Both have better biocompatibility than bismuth oxide. In light of the above-mentioned data, different compositions of Portland cement and nanoparticulated zirconium oxide or niobium oxide with diameters 74 nm and 83 nm, respectively, have been studied. Radiopacity, influence on pH, and antimicrobial activity of the novel nanofilled Portland cements were tested. Introduction of nano-ZrO_2_ and nano-Ni_2_O_5_ into Portland cement induces no influence on antimicrobial activity in comparison with pure Portland cement and does not change the pH values. Both nanofillers enable to obtain proper X-ray pictures, and can be considered as radiopacifying agents for endodontic sealers [132]. As hydroxyapatite was considered as a radiopacifier, it has been attempted to introduce it into endodontic cements. Hydroxyapatite is known as a well-tolerated material and possesses the ability to form a direct bond with bone and it exhibits an osteoconductive action [133]. Nanoparticulated hydroxyapatite (diameter ca. 26.8 nm) as a radiopacifier was introduced to methacrylate-based endodontic sealer by Collares et al. They have studied radiopacity, flow, and film thickness of the newly obtained nanosealer. They noted that the addition of up to 40% *w*/*w* nanoparticles presents similar flow and film thickness. On the other hand, the best radiopacity profile was observed for 10% of nanoparticles in the sealer [134]. Another impact on endodontic sealer was studied by Saghiri et al., who have investigated nano white mineral trioxide aggregates patented in the US. The studied sealers mainly consists of dicalcium silicate, tricalcium silicate bismuth oxide, and strontium carbonate. Particle size was placed in the range between 40 and 100 nm [135]. Authors have performed mechanical studies and assessed the surface area, setting time, microhardness and other properties. Performed studies have shown that the surface area increased significantly for the nanofilled sealer in comparison to the sealer without nanoparticles. Thanks to the higher surface area, setting time decreased from 43 min for non-nanofilled to 6 min for nanofilled sealer. Microhardness was checked under two pH conditions, at 7.4 and 4.4. At both pH values, the nanofilled sealer revealed significantly higher microhardness [136]. Authors have continued studies on nanomodified mineral trioxide aggregate and have performed push-out bond strength studies on sixty single-rooted human teeth. Strength values of 110 MPa, 138 MPa and 25 MPa were obtained for mineral trioxide aggregates, nanosized mineral trioxide aggregates and bioaggregates, respectively. Good quality cement should stay at root canal and integrate with canal walls even under mechanical stress. According to the obtained data, nanomodified mineral trioxide seems like a good candidate for endodontic sealer [137]. The studies were continued and calcium ion release was tested. The analysis has indicated that calcium ion level in the surrounding environment was the highest for the nanomodified trioxide aggregate in comparison to the mineral trioxide aggregate and its modification with 1% of methylcelulose. Increase in calcium ion concentration simultaneously caused an increase in the pH value. High pH stimulates cell division. However, calcium ions stimulate human dental pulp cells, and enable bone regeneration, endothelial cells proliferation and dentinogenesis [135]. Naseri et al. have checked the influence of particle size of calcium hydroxide on radicular dentin microhardness. It was concluded that nano-calcium hydroxide does not change the microhardness in comparison to the conventional formulation of this drug [138]. 

Newly designed endodontic materials should not exert any side effect on the body. Complex studies of new endodontic cement based on nano-calcium silicate with a diameter between 117 and 477 nm and hydroxyapatite have been performed. Authors have tested the mechanical properties as well as the genotoxic potential. Genotoxic tests have been performed on lymphocytes isolated from blood of five young healthy donors. DNA damage in comet assay and cell viability were assessed. Genotoxicity of calcium silicate was evaluated as low; it was the same for hydroxyapatite. Obtained data allowed to conclude that genotoxicity values of the calcium silicate and hydroxyapatite are lower when compared with the genotoxic profile of the ingredients [139].

## 6. Prosthodontics

Prosthodontics is a subdivision of dentistry also termed as prosthetic dentistry. It focuses on restoring the missing teeth in the tooth arches. It utilizes implants, single tooth restorations as well as removable prostheses (dentures). All the mentioned areas are artificially linked within the term. In prosthodontics, specific materials are used. They are exposed to different factors causing their wear. Thus, in this branch, similar to others, nanotechnology has been used [140].

One of the most important factors which classifies the quality of a denture is wear resistance. The perfect denture should reveal wear similar to the natural teeth. Ghazal et al. have studied susceptibility to wear of different materials. They have used denture teeth manufactured with feldpathic ceramic—FC, nanofilled composite resin (NCR) and acrylic resin. NCR-based teeth as a nano-filler contained SiO_2_ at the size of 12 nm (36–42 wt.%). In this study, authors have applied a chewing simulator and each group was subjected to 200,000 chewing cycles. Then, the studied materials were assessed using a laser scanner, an optical microscope and a scanning electron microscopy technique. Tests have been performed via loading buccal cusps with different combination of teeth including natural ones. Authors have concluded, in agreement with previous studies, that ceramic denture reveals highest resistance to wear, higher than even natural teeth. Therefore, ceramic partial dentures increase the wear of natural teeth. Interestingly, obtained data enable to conclude that nanofilled denture resin causes significantly low destruction of natural teeth, thus, this material is suitable for partial dentures [141]. Dentures based on nanocomposites consisting of poly(methylmethacrylate) (PMMA) as a composite matrix and montmorillonite nanosheets (MMT) as a modifier have been studied. Zheng et al. have checked the biocompability of these materials with cytotoxicity, acute systemic toxicity, oral mucous membrane irritation, guinea pig maximization and mouse bone marrow micronucleus tests. Based on the obtained results, authors have concluded that the presented composite possesses good biocompatibility for denture basal material [142]. The studies on this nanocomposite were continued by Wang et al., who have performed thermal stability tests. These experiments have shown high thermal stability and considered the studied material as very promising for denture building [143]. Often, the problem in manufacturing of dentures based on acrylic resin is the polymerization shrinkage. During the polymerization process, 21% of volume shrinkage of resin has been observed. Therefore, the carbon nanotubes were introduced into the resin. Results have indicated a reduction in shrinkage (no visible shrinkage) of resin with nanotubes at a concentration of 0.5% (wt.%) [144]. 

One of the greatest problems in prosthodontics is the development of denture stomatitis.

It was proven that the *Candida* species, especially *C. albicans*, play a key role in the initiation and progress of these lesions [145,146,147]. Thus, scientists are looking for highly active tools to combat fungi. A denture based on acrylic resin with silver nanoparticles as an antifungal agent was prepared. It is well known that silver nanoparticle is able to combat *Candida* infections at 1 mg/mL, as proven by Panáček et al. [148]. Wady et al. have created a denture based on acrylic resin modified with silver nanoparticles. They have performed experiments to check the inactivation of *C. albicans* both in planktonic and biofilm forms. Obtained results have indicated that unloaded nanoparticles work strongly against *Candida*. Unfortunately, silver nanoparticles bound in a resin matrix possess no activity. The explanation for this can be the low release of silver ions from the highly hydrophobic acrylic resin [149]. Activity of silver nanoparticles introduced into a polymerized denture was further researched by Nam et al. They have performed antifungal activity experiments on polymerized acrylic discs with silver nanoparticles. Acrylic discs with 0; 1.0; 5.0; 10.0; 20.0; and 30 wt.% of nanoparticles were treated with a suspension of *C. albicans* strain. Then, discs with fungi were incubated in 37 °C for 24 h. It turned out that discs containing 20 and 30 wt.% of silver nanoparticles worked as latent antifungal materials. Additionally, they have performed elution tests, and concluded that maximal concentration of Ag^+^ was achieved at 0.356 mg/L. Simultaneously, the minimal inhibitory concentration (MIC) for silver ions was assessed and was equal to 3.0 mg/mL. Authors have concluded that antifungal activity is linked with direct interaction of fungi with denture surface because after nanosilver release, the MIC was not achieved. Authors have also performed thermal analysis, which showed that incorporation of nano-filler improved thermal stability. Color evaluation was performed using a spectrophotometric method and it was concluded that loss of color is high and should be improved [150].

## 7. Periodontics

Periodontitis is a biofilm-related inflammatory periodontal disease, which is the major cause of tooth loss. Its etiology is multiple and mainly associated with a shift in the content of the oral microflora or the destructive host inflammatory response. During the development of periodontitis over an extended period of time, the oral microbiota shift from one consisting primarily of Gram-positive symbiotic aerobes to another consisting primarily of Gram-negative pathogenic anaerobes. Therefore, antibiotics, antiseptics, and probiotics are so often used for the control of periodontitis microflora. As far as the harmful inflammatory background of periodontitis is concerned, matrix metalloproteinases, mainly produced by infiltrating neutrophils, are believed to act as mediators of tissue destruction. Matrix metalloproteinases additionally contribute to bone resorption, which make these enzymes an essential cause of tissue destruction and alveolar bone loss. The most successful treatment approaches are directed at the bacterial and inflammatory factors of the disease [151]. Regeneration of periodontal structures includes the regeneration of four different tissues—cementum, periodontal ligament (PDL), alveolar bone, and gingiva. The wound healing process is exposed to bacterial biofilms and mechanical forces. Plenty of growth factors and cytokines are involved in either speeding up or modifying the healing process, which depends on the migration and differentiation of several different cell populations [152]. The primary therapy of choice is scaling and root planning SRP, often in combination with antimicrobial therapies and host modulation therapy [151]. Photodynamic antimicrobial chemotherapy (PACT) as a nanotechnology approach is considered as an interesting alternative to chemical antimicrobial agents to fight periodontitis [153]. If the resistance against antibiotics increases, PACT becomes a valuable alternative for most indications of antibiotics or for immunosuppressed patients [2,154]. Moreover, according to some authors, because of their drawbacks antibiotics are considered as an adjunct to local periodontal therapy or systemic chemotherapy in conjunction with mechanical debridement (mechano-chemotherapy) [155]. Nowadays, PACT is becoming more and more popular for treatment of periodontal infections, because this treatment does not require anesthesia and can destroy bacteria in a short period of treatment time [156]. Many studies have been carried out to investigate whether PACT improves periodontal outcomes in comparison to SRP or no treatment.

**Figure 7:**
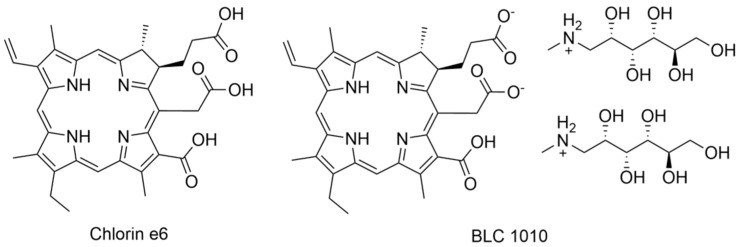
Structures of chlorin e6 and BLC 1010 PSs.

Firstly, the usability of PACT has been evaluated in the in vitro model against bacteria species typical for periodontitis. Pfitzner et al. assessed a novel strategy for killing periodontopathogenic bacteria using PACT. They compared the following PSs: chlorin e6 with a structure of basic porphyrin skeleton, BLC 1010, and BLC 1014 (Figure 7).

The scientists noticed in the CFU method that the anaerobic bacteria *Porphyromonas gingivalis*, *Fusobacterium nucleatum*, and *Capnocytophaga gingivalis* can be inactivated completely by irradiation with a very low light dose of 5.3 J/cm^2^ in the presence of 10 μM chlorin e6 and 10 µM BLC 1010. Zones of inhibition on agar plates were induced with the PSs chlorin e6 and BLC 1010, whereas BLC 1014 failed to produce a zone of inhibition. In the bacterial viability test kit, the PS BLC 1014 caused the lowest photodynamic effect in comparison to the others. PACT with chlorin e6 and BLC 1010 turned out to be effective for suppressing anaerobic periodontopathogenic bacteria [157]. These results clearly indicated that the development of a suitable PS is very complicated and it could be concluded that physical properties, such as solubility and bacterial membrane targeting, are crucial next to ROS generation (no activity in zone of inhibition test vs. CFU method). Therefore, in the initial studies of the new approach, seemingly negative activity can be observed. Some fluctuations in the level of biological activity is linked with the light source. Matevski et al. investigated the capability of red-filtered xenon lamp to activate toluidine blue O (TBO) in vitro to observe if it had similar potential of helium–neon (He-Ne) laser irradiation of a PS in eradicating periodontal pathogens. They subjected *Porphyromonas gingivalis* to irradiation with a light dose of 2.2 J/cm^2^ and 50 µg/mL of TBO concentration. A bacterial reduction of 2.43 ± 0.39 logs was achieved with the He-Ne laser excitation and 3.34 ± 0.24 logs with the lamp. With the increase in light dose, the decrease in bacterial survival was observed, whereas no such relationship was found for the drug concentrations. The maximal bacterial growth inhibition after their exposition to PACT (*P. gingivalis*, *P. intermedia, F. nucleatum, and A. actinomycetemcomitans*) led to a reduction of 5 logs, whereas only *B. forsythus* demonstrated relatively reduced sensitivity. Addition of serum or blood at 50% *v*/*v* to the pathogen suspension prior to irradiation, diminished killing, but after washing off the serum, killing returned to 5 logs for all species tested, except *Bacteroides forsythus* (3.92 ± 0.68 logs reduction). PACT appeared to be effective in fighting periodontal pathogens with the use of xenon lamp at 10 J/cm^2^, 100 mW/cm^2^ and 12.5 µg/mL TBO, even in the environment of serum and blood at high concentrations [158].

The usefulness of the PACT method in the treatment of periodontitis has been extensively evaluated in in vivo models. Sigush carried out studies to check the suitability of PACT as a possible alternative to conventional methods for suppressing periodontopathogenic bacteria. They infected beagle dogs with *Porphyromonas gingivalis* and *Fusobacterium nucleatum* in all subgingival areas and monitored clinical parameters, such as increase in redness and bleeding on probing. For the treatment, two PSs, chlorin e6 and BLC1010 (Figure 7), and a diode laser with a wavelength of 662 nm and a maximum power of 2.5 W, were applied. The light irradiation lasted 20 s per tooth and constituted a maximum energy density of 12.7 J/cm^2^ per site. The animals were divided into treatment and control groups (laser only and no treatment). PACT led to a significant reduction in redness and bleeding on probing. Periodontal inflammation was clearly reduced and the species *P. gingivalis* was markedly suppressed due to the use of chlorine e6 [159].

Polansky et al. carried out studies to assess the bactericidal potential and clinical effects of PACT in the treatment of periodontitis. Fifty-eight subjects with chronic periodontitis (CP) were enrolled in the clinical pilot trial. Each patient exhibited at least three active periodontal pockets of 5 mm or deeper, bleeding on probing (BoP) and the presence of *P. gingivalis*. *P. gingivalis* was markedly reduced both in the laser and control group (ultrasound treatment). No significant reductions in *T. forsythia* and *T. denticola* were observed in either group. No significant difference in the microbial parameters was observed between the laser and the control group. The mean probing pocket depths (PPDs) decreased from 5.79 to 4.55 mm in the laser group and from 5.54 to 4.51 in the control group. BoP decreased from 100%, evaluated at the beginning, to 47% in the laser group and to 59% in the control group. A single cycle of PACT did not act as an adjunct in ultrasonic periodontal treatment. No further reductions in PDs and BoP were found. Similarly, no additional effects with regard to eradicating bacteria were demonstrated in comparison to conventional treatment alone. PACT failed to improve probing depths (PDs), attachment levels and microbiological parameters compared with ultrasonic therapy alone, which indicates no extra benefit in combining ultrasonic therapy with adjunctive PACT [160]. Similar conclusions have been drawn by Rühling et al., who performed comparative studies on the short term application of a course of single PACT and a conventional ultrasonic debridement (UST) in patients with persistent pockets. For UST, the mean PD was reduced from 5.3 to 4.5 mm and for PACT it was reduced from 5.3 to 4.7. Microbial counts decreased by about 30% to 40% after debridement but increased to the initial values at three months interval in cases of both modalities. It turned out that PACT had no long-lasting impact on the microbial counts of perio-pathogens after three months [161]. Queiroz et al. investigated the microbiologic effects of adjunctive PACT on nonsurgical periodontal treatment of smokers with CP. Twenty smokers with CP had two contralateral teeth randomly assigned to receive SRP or SRP followed by a single episode of PACT. For PACT, a diode laser with a wavelength of 660 nm and a maximum power of 60 mW/cm^2^ was used along with a phenothiazine PS in a concentration of 10 mg/mL for 10 s. After periodontal treatment, the levels of some bacterial species decreased and some other species increased. *T. forsythia*, for example, increased in the test group and decreased in the control group, while *P. gingivalis* increased in the control group and remained stable in the test group, and *T. denticola* decreased in both the groups. However, statistically significant differences could not be detected between the groups. The adjunctive effect of a single episode of PACT to SRP in smokers failed to demonstrate microbiological improvements as smoking impaired periodontal healing [162]. On the other hand, Lulic et al. confirmed the benefits of repeated adjunctive PACT to conventional treatment of residual pockets in periodontal maintenance patients. Ten maintenance patients with 70 residual pockets were enrolled for treatment: five times in two weeks with PACT (test group) or non-activated laser (control group) following debridement. The diode laser was operated with a wavelength of 670 nm and a power density of 75 mW/cm^2^ for 1 min., together with phenothiazine chloride as a PS. The patients were observed after one, three, six and twelve months. PPD, clinical attachment level (CAL) and BoP were compared at three, six and twelve months. Better parameters were obtained for PACT applied repeatedly. In this test, greater PPD reductions and higher CAL gain were observed in patients after six months. Also, BoP percentages decreased significantly in the test group after three, six and twelve months [163].

As mentioned above, for now, PACT cannot become a method of choice in the treatment of periodontitis. Therefore, researchers have started to test the impact of PACT on the conventional protocols. Bottura et al. studied the effectiveness of laser and PACT as an adjunct SRP in the treatment of corona-induced periodontitis in rats immunosuppressed with tacrolimus. The animals were divided into five groups, each comprising six rats. The first group was the control and they were only administered a saline solution throughout the study period of 42 days. The second group was treated with a saline solution and SRP; the third one received tacrolimus (1 mg/kg per day) and was subjected to SRP. The fourth group was treated identically to the third one and then administered a laser treatment. The fifth group was treated identically to the third one and then administered the PACT. Decreased bone loss was observed but not associated with the types of treatment administered to groups. Laser and PACT therapies turned out to be effective as adjunctive treatment to SRP in limiting bone loss induced by periodontitis in animals treated systemically with immunosuppressive tacrolimus [164]. The potential of PACT as an adjuvant protocol has been also reported by Braun et al. They investigated the efficiency of adjunctive PACT in CP. The study included twenty patients with untreated CP. All teeth were subjected to SRP. On the basis of a split-mouth design, some patients were additionally treated with PACT. Sulcus fluid flow rate and BoP were evaluated at the beginning, and one week and three months after treatment. Relative attachment level, PDs and gingival recession (GR) were assessed at the beginning and three months after treatment. The median values for PD, GR and RAL evaluated at the beginning did not differ for both the test and the control group. Values for RAL, PD, SFFR and BOP decreased three months after treatment in the control group, more significantly on the sites subjected to PACT. GR increased three months after treatment with and without adjunctive PACT, irrespective of the groups. In the study outcomes, a positive effect of the adjunctive use of PACT on CP treatment was confirmed [165]. The impact of PACT on the periodontitis parameters reported by Braun et al. has been confirmed by Christodoulides et al. They tried to compare the clinical and microbiologic effects of the adjunctive use of PACT to the non-surgical treatment of CP. Twenty patients were randomly subjected to SRP followed by a single episode of PACT (test group) or SRP alone (control group). Full-mouth plaque score, full-mouth bleeding score, PD, GR, and CAL were evaluated at the beginning and three and six months after therapy. The main result variables were changes in PD and CAL. At three and six months, a greater improvement in FMBS was observed in the test group, whereas CAL, PD, FMPS, or microbiologic changes at three and six months after treatment did not differ statistically between the groups. Additional application of a single treatment of PACT to SRP caused a higher reduction in bleeding scores in comparison to SRP alone, but there were no PD reduction and increase in CAL [166]. Promising impact of photodynamic tools was described by Giannelli et al., who studied the efficacy of photoablative and photodynamic diode laser in addition to SRP and SRP alone for the treatment of CP in a group of 26 patients. Each patient underwent two parallel treatments: the teeth on the test maxillary quadrant were subjected to laser treatment combined with SRP, whereas those of the contra-lateral control quadrant were subjected to sham-laser treatment combined with SRP. In the photoablative method, the gingival mucosa was irradiated with a diode laser operating at 810 nm wavelength (light dose of 66.7 J/cm^2^). At the next clinical stage, multiple photodynamic sessions were performed (once weekly, 4–10 applications, and mean ± SD: 3.7 ± 2.4) using a diode laser operating at 635 nm wavelength, with 100 mW output power, continuous wave and 0.3% methylene blue as a PS. PD, CAL, and BoP were evaluated at days 0 and 365. Polymorphonuclear leukocytes, red blood cells, damaged epithelial cells, and bacteria from gingival exfoliative samples taken at days 0, 15, 30, 45, 60, 75, 90, and 365 and were analyzed using cytofluorescence. At day 365, all the results were compared with the control quadrants. For the combined therapy of laser with SRP, a reduction in PD (−1.9 mm), CAL (−1.7 mm) and BoP (−33.2% bleeding sites), as well as bacterial contamination was observed. Diode laser treatment (photoablation followed by multiple photodynamic cycles), adjunctive to conventional SRP, substantially contributed to healing of CP [167]. PACT’s role as a significant adjuvant role has been also reported by Sigush et al., who evaluated the clinical and microbiological effects of PACT administered adjuvantly after SRP in patients with localized chronic periodontitis. The following clinical parameters were assessed during the initial examination and at 1, 4, and 12 weeks after PACT: plaque index (PI), reddening, BoP, PD, GR, and CAL. In patients with LCP subjected to PACT treatment, significant reductions in reddening, BOP, and mean PD and CAL were noted during the study period with respect to the controls. Four and twelve weeks after PACT, the mean PD and CAL differed significantly from the initial values and from those of the control group. The *F. nucleatum* DNA concentration in the PACT-administered patients 12 weeks after treatment decreased significantly in comparison to the initial level. The adjuvant application of the studied PACT method proved to be efficient in alleviating periodontal inflammatory symptoms and in the successful treatment of infection with *F. nucleatum* [168]. PACT, when used as an adjuvant tool, does not improve treatment effects in the long term, as reported by Abuderman and Muzaheed. They performed studies to compare the effectiveness of SRP with and without adjunct PACT in reducing a Gram-negative anaerobic bacterium, *Campylobacter rectus*, occurring in the oral biofilm of patients with periodontitis. Thirty chronic gastritis patients with Grade-B/Stage-II periodontitis were divided into two groups—a test group (SRP + PACT) and a control group (SRP alone). In all the subjects, full-mouth PI, BoP, PD, marginal bone loss and counts of *C. rectus* in subgingival oral biofilm were measured at the beginning and 12 weeks after treatment. As far as PACT is concerned, methylene blue (0.005%) was applied as a PS. Diode laser was operated at a wavelength of 660 nm with an energy fluence of 150 mW/cm^2^ for 60 s in all periodontally compromised pockets. At the beginning, all parameters were comparable in both the groups. At 12-weeks follow-up, PI, GI and PD were substantially lower in the test group in comparison to the initial parameters. The percentages of *C. rectus*-positive individuals were lower in the test than the control group at 12 weeks. Adjuvant use of PACT contributed to the elimination of *C. rectus* in the oral biofilm and the reduction in clinical parameters in patients with Grade-B/Stage-II periodontitis [169]. Reduction in viable bacteria has been also noted by Alhamoudi et al., who carried out a clinical trial to assess the efficacy of PACT as an adjunct to open flap debridement in the treatment of generalized aggressive periodontitis (GAP). The PI, pocket depth (PD), full-mouth probing depth, BoP, relative attachment loss, full-mouth relative attachment loss and the microbial levels of *Aggregatibacter actinomycetemcomitans*, *Porphyromonas gingivalis* and *Tannarella forsythia* were assessed at the beginning of the study and after three months. The patients of the test group were subjected to PACT after SRP. In terms of PACT, phenothiazine chloride (10 mg/mL) was applied as a PS and the site was irradiated with a diode laser at a wavelength of 660 nm, energy density of 0.6 J/cm^2^ and power of 60 mW/cm^2^. BOP appeared to be one of the clinical periodontal parameters that showed substantial improvement three months after the application of PACT. Both groups of patients revealed a reduction in the microbiological levels for *A. actinomycetemcomitans*, *P. gingivalis* and *T. forsythia* after three months. However, no significant differences in the levels of Ag, Pg, and Tf between the groups were observed [170] Similar findings have been shown by de Oliveira et al. They compared two approaches of treating GAP. Ten patients were subjected to either PACT or SRP with hand instruments. For the PACT group, a diode laser with a wavelength of 690 nm and a maximum power of 60 mW/cm^2^ was used along with phenothiazine chloride as a PS in 10 mg/mL of concentration. For clinical purposes, the following parameters were assessed at the beginning and three months after treatment with an automated periodontal probe: PI, gingival index (GI), BoP, PD, GR, and relative clinical attachment level (RCAL). Three months after treatment, the plaque results decreased and remained low. GI and BOP were reduced in both groups after three months. The mean PD decreased in the PACT group from the initial 4.92 ± 1.61 mm to 3.49 ± 0.98 mm after three months and in the SRP group from the initial 4.92 ± 1.14 mm to 3.98 ± 1.76 after three months. The mean RCAL for the PACT group was reduced from 9.93 ± 2.10 mm to 8.74 ± 2.12 mm and for the SRP group, it reduced from 10.53 ± 2.30 mm to 9.01 ± 3.05 mm after three months. The scientists obtained similar clinical results for both types of treatment (PACT and SRP) of GAP [171]. The problem of comorbidities is not without meaning for PACT efficacy. Al-Zahrani et al. investigated the effect of the adjunctive use of PACT on periodontal condition and glycemic control of patients with diabetes and periodontitis. They selected patients with type 2 diabetes and CP and divided them into three groups. Each group, consisting of 15 subjects, was assigned to different treatment modalities: SRP only, SRP plus systemic doxycycline, and SRP plus PACT. They compared the plaque and bleeding scores, PD, CAL, and glycosylated hemoglobin (HbA1c) levels at the beginning of the study and three months after periodontal treatment. Differences in the mean PD, CAL, plaque deposit, and BoP were found 12 weeks after treatment, whereas no significant differences in periodontal parameters and glucose levels were found for all groups. Reduction in the mean HbA1c level after treatment appeared in all groups but it was significant only for the SRP plus doxycycline group, which indicated that PACT does not contribute to non-surgical periodontal therapy in diabetic patients [172]. On the other hand, de Almeida et al. have performed studies on the potential of PACT in treating periodontitis in the presence of diabetes. Studies have been carried out using animal models (rats). Two hundred forty rats were divided into two equal groups: non-diabetic (ND) and alloxan diabetic (D). In all animals, periodontal disease was induced at the first mandibular molar. After the removal of the ligature 7 days later, all the rats were subjected first to SRP and next—after assigning them to a certain group—to irrigation with a saline solution (SRP group), irrigation with a phenothiazinium dye (100 mg/mL) (TBO group), a low intensity laser irradiation (660 nm and 24 J) (LLLT group), and PACT (TBO and laser irradiation group). The low intensity laser was a Gallium-Aluminum-Arsenide (GaAlAs) laser, with a wavelength of 660 nm. The therapeutic laser was released with a power density of 0.428 W/cm^2^ and a total energy of 24 J for the total area. After 1 min of TBO application, LLLT was applied using the aforementioned parameters. The animals from the control group treated via PACT revealed less bone loss than the SRP group, the TBO group and the LLLT group at all tested periods. Similarly, in the group with diabetes, the animals treated with PDT showed less bone loss than the animals from the other groups at all time points. The scientists confirmed the fact that cells in some pathologic states (with reduced redox potential) are more sensitive to irradiation. In case of diabetes, oxidative stress increases in the tissue and plasma due to the accumulation of advanced glycation end products, AGEs. These phenomena result from the continuous cycle of metabolic stress, tissue damage, and cellular death, leading to the production of free radicals and increased vascular dysfunction [173]. Chitsazi studied the potential of adjunctive PACT in the treatment of periodontitis. Twenty-four patients with GAP were subjected to SRP. The teeth of one quadrant of each arch with ≥4 mm of PD were additionally subjected to PACT (test group). A diode laser with a wavelength of 670–690 nm and a power of 75 mW for two minutes was employed in the test group after the application of toluidine blue photosensitizer dye. The control group included selected teeth from the contralateral quadrant (SRP only). Treatment groups showed an improvement in all the clinical parameters: CAL, PI, BoP, PD and GR, and a marked decrease in the counts of *A. actinomycetecommitans* at 90 days compared to the initial counts. There were no significant differences between the two groups after three months and no additional benefits from PACT as an adjunctive treatment for patients with GAP were noted [174]. It could be concluded that PACT as an adjuvant tool improves some parameters and has no impact on others. 

Photodynamic methods work via multi-target pathways and in several experiments the potential of modulation of cytokine levels and its consequences were reported.

Braham et al. demonstrated that due to its strong antibacterial potential, PACT can be applied as an adjunctive modality in combination with SRP in the treatment of periodontitis. The scientists observed that PACT treatment can simultaneously combat *P. gingivalis* and inactivate its virulence-associated protease. It also neutralizes host cytokines, tumor necrosis factor-alpha (TNF-a) and interleukin (IL)-1b, which are the mediators of tissue destruction in many immuno-inflammatory diseases, impairing periodontal restoration. It turned out that PACT treatment in vitro resulted in a 4 log reduction in the bacterial growth of *P. gingivalis*, pathogens associated with the destruction of the host tissue by expression of proteases able to degrade host connective tissue and disarm the innate host response. Protease inactivation was observed at lower concentrations of a PS and less time of light exposure. PACT treatment provided promising healing results for periodontitis due to its wide mechanisms of action. It increased bacterial elimination, and inactivated both bacterial virulence factors and host cytokines [175]. What is interesting, SRP alone could reduce cytokine levels. De Oliveira et al. studied cytokine levels in the gingival crevicular fluid (GCF) of patients with GAP. Ten subjects were randomly treated with PACT or SRP. For PACT, a diode laser with a wavelength of 660 nm and a maximum power of 60 mW/cm^2^ was used with a phenothiazine PS in a concentration of 10 mg/mL. After collecting the GCF samples, the concentrations of tumor necrosis factor-alpha (TNF-α) and inducing periodontal bone resorption, receptor activator of nuclear factor-kappa B ligand (RANKL) were measured [176,177]. It turned out that both non-surgical periodontal treatment with PACT and SRP resulted in reductions of TNF-α level 30 days after therapy. Levels of TNF-α and RANKL at the different time points were similar in both groups, which confirmed similar effects of PACT and SRP in patients with GAP [177].

Giannopoulou et al. investigated the local biologic effects of PACT, diode soft laser (DSL) therapy, and conventional deep SRP in residual pockets of 32 patients with periodontitis and persistent sites of the disease with PDs > 4 mm and bleeding on probing. After the debridement of residual pockets with an ultrasonic device, they were randomly subjected either to PACT, DSL, or SRP. In DSL treatment, subgingival irradiation was performed using a diode laser with a wavelength of 810 nm and power output of 1 W for 60 s. In PACT treatment, a diode laser with a wavelength of 660 nm and an output power of 100 mW was used. A 100 mg/mL phenothiazine chloride sterile solution was placed into the pockets, incubated for three minutes, rinsed with water and subsequently irradiated with the diode laser for one minute per tooth. Samples of GCF were collected before treatment, after 14 days, and at 2 and 6 months to determine the levels of 13 cytokines and 9 acute-phase proteins. For measurement, a bead-based multiplexing analysis system was employed. Compared with the initial content, levels of interleukin-17, basic fibroblast growth factor, granulocyte colony-stimulating factor, granulocyte macrophage colony-stimulating factor, and macrophage inflammatory protein 1-α were reduced 14 days and 2 months after treatment. The differences remained significant during the study, except for granulocyte colony-stimulating factor. The levels of α-2 macroglobulin, haptoglobin, serum amyloid P, procalcitonin, and tissue plasminogen activator increased at six months. Levels of all indicators of inflammation present in GCF significantly changed after each modality treatment. There was no specific observation of any expression of inflammatory mediators associated with DSL or PACT [178]. The long-term impact of PACT on periodontal parameters was studied by Lui et al. who performed a short-term clinical trial. PACT was included to overcome the limitations of SRP and to reach the deep periodontal pockets and furcation. Twenty-four nonsmoking adult patients were subjected to scaling and root debridement with or without one course of adjunctive PACT and low-level laser therapy within five days. After the procedure of scaling and root debridement, the test teeth were irradiated using a low-level diode laser with a wavelength of 940 nm, for 5–10 s and with no more than 4 J/cm^2^ of energy. The next day, the same tested teeth were subjected to PACT. Topical anesthetic gel of 20% benzocaine was initially applied, then washed off and the periodontal pockets were filled with a PS. A methylene blue solution of 1% was left for three min and for the irradiation the diode laser was operated at a peak power of 5.0 W. After three days, the patients received the final low-level laser therapy on the test teeth. Clinical parameters such as plaque, BoP, PD and GR were observed at the beginning, one and three months after the treatment and compared to the control teeth. Moreover, interleukin-1b levels in GCF were determined at the beginning, after one week and after one month. The number of sites with BoP and mean PD of the test teeth decreased after one month. A reduction in GCF volume was found in both groups at one week and a further decrease was observed at one month in the test sites. For the test sites, a much lower level of interleukin 1b in GCF at one week was determined than for the control sites. At three months, periodontal parameters did not essentially differ between the test and control teeth [179]. Similar conclusions have been confirmed by Monzavi et al. They performed a full-mouth double-blind randomized controlled clinical study. Fifty patients were enrolled in the study and all received SRP. Next, the patients were randomly assigned either to the test group (PACT + SRP) or the control group (SRP). For PACT, ICG was used as a PS and a diode laser was operated at a wavelength of 810 nm and a power of 200 mW. The modality was applied after 7, 17 and 27 days. BoP, CAL, PI, PPD, full-mouth plaque score and full-mouth bleeding score were monitored at the beginning and after one and three months. Improvements in BOP, PPD and FMBS were demonstrated in the test group, whereas no significant differences in PI, FMPS and CAL were observed between both groups. Application of PACT with IC led to complete resolution of inflammation and an essential decrease in periodontal pocket. Nevertheless, the combination of PACT and SRP did not outweigh conventional SRP in the aspects of clinical attachment gain and plaque score [180].

Nanoparticles in periodontitis treatment are not only focused on the PSs for PDT; there are a few examples of other approaches.

Hayakumo et al. investigated the suitability of ozone nano-bubble water (NBW3) as an irrigation adjunct for periodontal treatment due to its clinical and microbiological effects. Twenty-two patients were randomly divided into two treatment groups. One group received full-mouth mechanical debridement with tap water (WATER) and the latter one received full-mouth mechanical debridement with NBW3. Clinical parameters were examined at the beginning and four and eight weeks after treatment, whereas microbiological parameters were examined before and after treatment and at one and eight weeks after treatment. Improvements in clinical parameters were observed in both groups. The decrease in PPD and the increase in clinical attachment after four and eight weeks in the NBW3 group were greater than those in the WATER group. Additionally, the mean total number of bacteria in subgingival plaque decreased within the studied groups. NBW3 turned out to be a promising adjunct to periodontal treatment due to its potent antimicrobial effects, high level of safety, and long storage stability [181].

**Figure 8:**
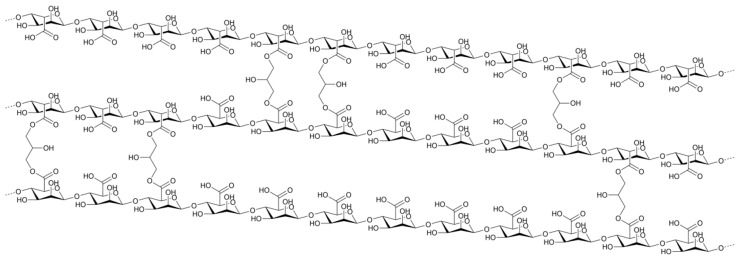
Visual representation of cross-linked alginate.

On the other hand, Johnston et al. proposed placing biomaterials inside the periodontal pocket to treat periodontal disease. They prepared drug-loaded cross-linked and plasticized alginate fibers (cl-PAFs, Figure 8) for prolonged drug delivery via extrusion-gelification. The following four formulations were used: drug-free, ciprofloxacin-loaded, diclofenac sodium-loaded and ciprofloxacin/diclofenac sodium-loaded fibers. Rigid fibers were formed at high cross-linker concentrations, whereas weaker fibers were obtained with low strain values, after addition of plasticizer. It was associated with the cohesive energy density, porosity and space filling. The concentration of cross-linker and amount of plasticizer had an impact on drug entrapment and release, respectively [182].

## 8. Implants

Implants are widely used in dentistry. In fact, the most suitable material utilized clinically in dentistry is titanium [183]. Titanium implants have variety of advantages, such as good biocompatibility and mechanical properties, and in general sufficient corrosion resistance. Additionally, titanium is rather unlikely to induce immunological reactions [184,185]. Although the acidic and saline conditions inside the oral cavity can induce electrochemical reactions between the implant and tissues, the corrosion resistance of titanium implants is not absolute. Implants under such conditions are still prone to release wear particles and ions, which can result in local immune response and weakening of the material and finally in implantation failure [185,186]. Another factor affecting the corrosion is the influence of microorganisms. Effects of their enzymatic activity may result in the loss of surface passivity and therefore in increased corrosion rate [186].

Taking the above into account, new methods are being developed to employ nanostructures for increasing osteophilicity.

Although titanium implants are most extensively used in dentistry, their properties are far from the ideal. Uncoated implants with fixed surfaces have relatively poor osseointegration, which results in impaired long-term healing [187]. It appears that the roughness of the surface is important, as pores enhance adsorption and proliferation of proteins and tissues on the implant [183,188,189,190,191]. Another advantage of implant coating is the rise of stress transfer, as pointed by Cheng et al.—thicker coating can result in better stress reduction and nanoporous structure can be responsible for more uniform stress distribution [10]. It should be emphasized that different modifications may be beneficial in contrasting terms, as shown by de Barros et al. They concluded that nano-modified surface could provide benefits in the early stages of bone healing, whereas micro-scale modification had better long-term outcomes [192].

Surface modification of titanium implants also allows for coating with different materials, mainly derived from calcium phosphate, such as hydroxyapatite (HA). Those materials are similar to bone apatite, and therefore their presence can improve the bone filling around the implant [189]. This phenomenon can result in the precision of surgical fitting and decrease healing time. It also appears that stronger attachment and spreading of the cells on the uncoated surface do not necessarily mean higher cell activity, as shown by Meirelles et al. on a rabbit model. In fact, implant with plasma-sprayed HA had better developed cells than pure Ti, despite their weaker attachment and spreading. However, due to similar nanotopography between coated and uncoated ones, the studies of Meirelles et al. did not show significant advantages of HA over pure Ti [189]. Instead of HA, Yokota et al. coated the titanium implant with amorphous calcium phosphate (ACP) using the magnetron sputtering technique. Results of their studies clearly indicate rapid resorption in vitro, as well as improved long-term bone formation in a rabbit model, compared to the non-coated titanium implants [193,194]. Other studies, such as the one performed by Alghamdi et al. in dogs model with nano-calcium phosphate (nano-CaP) and collagen coatings, indicate that the influence of the coating can be more complex. Alghamdi et al. concluded that despite better bone deposition near the implants, supportive effect of CaP was not observed, similar to the study by Meirelles et al. They hypothesized that this can be associated with possibly extremely rapid dissolution of the coating, too poor osseous environment or the source of collagen [195]. HA coatings appear to be successful also in human models, as shown by De Wilde et al., who compared it to pure titanium implants and described no significant differences in biocompatibility [196] ncorporation of other metals into HA coating can also provide interesting results, as shown by Zhao et al., who substituted HA with Zn using electrochemical methods. They compared HA and Zn-HA in rabbit models and did not observe significant differences in short-term evaluation, whereas in long-term studies Zn-HA showed more significant improvement in bone area. This was associated with similar morphology of Zn-HA and bone and this indicated that substitution with Zn may be highly beneficial [197] nstead of incorporation of other metals, there are also possibilities of connecting HA with anticoagulant, as showed by Bozzini et al., who prepared HA-heparin composites in order to enhance the bioactivity of the coating. Comparison of cell viability on non-coated, HA-coated and Heparin-HA-coated surfaces indicated better cell viability on both HA-coated implants (similar for both) than on bare Ti ones. It also appeared that the composite grows on the implant surface as nanowires, possibly resulting in the promotion of bone ingrowth and osseointegration [186]. However, despite their promising properties, HA coatings are often characterized with poor mechanical properties due to their brittle structure [198].

Another type of coating consists of biomimetic composites, as shown by Lee et al., who described the gelatin–gold composite. It appeared that the presence of gold and fully resorbable gelatin had a positive impact on the growth and survival of osteoblasts in vitro. Lee et al. also showed that osseointegration can be induced by appropriate growth factor signaling [190]. Biomolecules have also been investigated by Jin et al. They utilized hyaluronic acid–collagen system along with basic fibroblast factor in order to mimic the extracellular matrix and its functions. The system was shown to be successful in stimulating attachment and growth of gingival fibroblasts [199] oactive nanocomposites based on glass were also presented. Such an approach was shown by Mehdikhani-Nahrkhalaji et al., who utilized Ti implants coated with nanocomposite consisting of poly(lactide-*co*-glycolide) (PLGA), bioactive glass and HA. Studies have proven that the nanocomposite stimulated the attachment and viability of human-derived stem cells in vitro, as well as rapid formation of bone-like apatite in rabbit model, which was associated with pores formation due to PLGA degradation [200].

Instead of pure titanium, its alloys can be used as well. Addition of other elements, such as aluminum and vanadium, increases the mechanical properties and fatigue resistance of implants and does not suppress osseointegration. Mainly, the commercially used alloys are Ti_6_Al_4_V and Ti_6_Al_7_Nb (90% Ti, 6% Al and 4% V or 87% Ti, 6% Al and 7% Nb, respectively) [188,193,201]. However, those materials can be prone to toxic ion release and their use can result in inflammation and implant failure [188,201]. Overcoming this issue can be achieved by using appropriate coatings which may be crucial for the development of better implants. As an example, Metzler et al. investigated the influence of microwave plasma chemical vapor-deposited diamond coating on titanium alloy (Ti_6_Al_4_V) and found that this coating slightly increased the bone-to-implant contact, although it was not statistically significant. They also pointed out that the diamond coating reduces debris and the release of wear particles [188]. Another idea to overcome those issues was presented by Elias et al., who utilized a modified alloy, marked as Ti G4 Hard alloy, instead of the standard Ti6Al4V. They have proven good mechanical properties of the modified alloy which also does not release any toxic ions. Clinical tests also confirmed good osseointegration properties of the alloy [201].

Titanium oxides have been adapted as well. Wang et al. described the use of hierarchical nanostructure consisting of self-assembled TiO_2_ nanotubes. The elements of the coating appeared to have similar dimensions to collagen fibers in a rat model. Wang et al. pointed out that the hierarchical nanostructure is characterized with highly increased surface roughness and high hydrophilicity. This can be associated with good mechanical properties, and better attachment and growth of osteoblasts, compared to smooth and micro-treated (acid-etched) surfaces. Wang et al. also suggested that the nano-TiO_2_ interface can stimulate the formation of hydroxyapatite, thereby improving osseointegration [202]. Similar conclusions were drawn by Moon et al., who utilized surface oxidation to obtain combined micro- and nanostructures and evaluated their osseointegration in a rabbit model. They concluded that the combined structures enhanced the activity of alkaline phosphatase (ALP), and therefore stimulated the mineralization [203]. One of the important factors determining the biological response to implants is their wettability. It appears that Ti- and TiO_2_-based implants are prone to functionalization with UV light, which allows control over their hydrophilicity, as shown by Al Qahtani et al. They concluded that different ranges of UV can turn various hydrophobic surfaces of implants into hydrophilic ones, depending on their structure and properties [204].

Nanoparticles are under investigation to determine their potential in curing peri-implantitis.

Peri-implantitis constitutes a serious problem with dental implants, often leading to failure of implants and even deterioration of the bone support. The main factor of peri-implantitis occurrence is inflammation, associated either with mechanical influence or bacteria action, mainly by a biofilm-forming species. Thus, it is important to maintain cleanliness of the implant surface, as well as ensure decontamination of the place where the implant has to be placed [191,205].

In order to overcome the bacteria-induced peri-implantitis, various strategies have been proposed, including treatment with antiseptics (such as chlorhexidine), PDT, or antimicrobial coatings of the titanium surface [206].

Shibli et al. indicated that PDT may be successful in peri-implantitis treatment in a dog model when combined with guided bone-regeneration (a technique of directing the bone growth using barrier membranes). Use of toluidine blue O along with GaAlAs diode laser appeared to be successful against periodontal pathogens, and resulted in improved healing process [191]. Studies with methylene blue, as reported by Marotti et al., also suggested that PDT may be beneficial if used with conventional disinfectants, such as chlorhexidine. In addition, use of laser alone showed lesser activity, which was explained as a hidden photodynamic effect due to synthesis of porphyrin pigments by mouth bacteria [207,208]. Also, Schär et al. investigated phenothiazine-mediated PDT in order to compare it with another non-surgical treatment, local delivery of minocycline. Similar to Marotti et al., they have proven that usage of PDT along with antibiotics remains effective (both ~3 log reduction) [205].

Huang et al. showed that nano-structured HA can act as antimicrobial agent itself. Nano-HA appeared to be more active against Gram-positive rather than Gram-negative bacteria. However, antibacterial activity along with growth enhancement and differentiation of osteoblasts seems to be beneficial [206]. Antibacterial properties of HA nanocomposite were also reported by Mehdikhani-Nahrkhalaji et al. [204,209]. Dorkhan et al. suggested that oxidized Ti and Ti6Al4V-possessing nano-structured surfaces can lower the adherence of oral biofilm-forming bacteria [210]. In further research, they pointed out that implant surface oxidation does not interfere with the adherence of keratinocytes, fibroblasts and osteoblasts. These findings indicate that proper surface modification could promote adherence of cells and simultaneously limit the adherence of bacteria. However, they also concluded that the presence of oral bacteria can significantly diminish adherence of the desired cells; hence, there is a need to initially control the population of microbes [211]. Incorporation of silver nanoparticles into implant coating was also utilized in order to obtain a nanoporous layer with antibacterial properties. Massa et al. used systems based on starch-capped silver nanoparticles doped with nanoporous silica, which showed bacterial reduction and inhibition of the biofilm formation. Although the reduction was insignificant (<0.1 log), those properties along with potential good osseointegration due to highly ordered nanoporosity seem to be a promising approach in the development of novel antibacterial coatings [212]. Another idea associated with silver placement was presented by Godoy-Gallardo et al., who utilized electrochemical process involving silver nitrate. The obtained Ag/AgO/Ag_2_O coatings presented some antibacterial properties (up to 1.5 log for *S. sanguinis* and 0.5 log for *L. salivarius*), while fibroblast viability was unaffected. Additionally, the silver and silver oxide coating had increased roughness and wettability, compared to untreated Ti implants [213]. Similarly, coating of the implants with Zn can also have good antimicrobial properties. Such an idea was presented by Memarzadeh et al., who utilized ZnO and mixed ZnO-HA coatings on glass implants. The coatings were tested in vitro and both of them revealed good antibacterial properties (up to 3 log). Additionally, these coatings did not interfere with human cells proliferation [214] Hayek et al. investigated the effects of PACT and the conventional technique of microbial reduction in ligature-induced peri-implantitis on around 18 dental implants in 9 Labrador retriever dogs. Peri-implantitis is an infectious illness affecting gingiva and the supporting bones, caused by a specific microflora colonization on the implant surface. Eighteen third premolars were extracted and the implants were submerged. Peri-implantitis was induced after osseointegration. After 4 months, the ligature was removed. Natural bacterial plaque accumulated for another 4 months. The animals were then randomly subjected to various types of treatment. One group was treated conventionally with mucoperiosteal flaps for scaling the implant surface and chlorexidine irrigation. In the latter, only mucoperiosteal scaling was applied and followed by PDT. The peri-implant pockets were filled with a paste-based azulene PS and exposed to a GaAlAs low-power laser (λ = 660 nm and light dose = 7.2 J/cm^2^). Microbiological samples were taken just before and after the therapy. Before treatment, one implant was analyzed via scanning electron microscopy for the contamination check. The outcomes were similar for both groups as they demonstrated significant decrease in *Prevotella* sp., *Fusobacterium* sp., and *S. pyogenes β-hemolyticus*, which may indicate the potential of PDT against periimplantitis pathogenic microorganisms [215].

Osseointegration (or osteointegration) is the process by which living bone tissue grows and attaches to the surface of an artificial implant, such as a dental implant. It is favorable that the material of the implant offers the possibility of fast and complete bone integration, resulting in lower psychophysical distress of the patient. Evaluation of the implant strength integration includes biomechanical evaluation, such as pull-out test and histological evaluation allowing to observe calcification process, and other imaging methods like SEM techniques with the ability to evaluate the topology of the link between bone and the implant [216,217]. Usage of these basic techniques opens a wide scope to develop and optimize different implant materials. The history of osseointegration goes back several centuries, but critical research on this process began only in the 20th century [218]. In 1952, the Swedish orthopedist Per-Ingvar Brånemark, who was researching blood circulation, accidentally discovered that titanium had integrated into the tissue of a rabbit that had a titanium tube implanted in its bone [219,220] discovering the phenomenon, Brånemark began investigating osseointegration in order to develop durable orthopedic implants [219]. In 1965, he designed the first prototype of a dental implant that could be integrated into the patient’s bone and was able to hold the denture. Since 1971, the Brånemark system has been introduced to the market and it is now estimated that one million endosseous dental implants are placed each year. Today, osseointegration has become a commonly used method for treating teeth and orthopedic implants [221]. Osseointegration is a relatively new issue and it was first fully described only in 1977. The first full definition was presented in 1981 in the groundbreaking work of Albrektsson et al., but it has been undergoing dynamic changes to this day [222].

Osseointegration is a physiological process in which the integration of living human tissues with external materials, such as titanium or platinum, is observed in such a way that a permanent connection between the implant and the tissue (e.g., bone tissue) occurs [223]. This phenomenon enables the development of artificial body implants that are stable, reliable and can be used for many purposes, including dentures, dental implants and more. According to the definition of osseointegration discussed earlier, in the case of orthopedic and dental implants, it is crucial to insert the surgical implant into the bone in such a way that the bone tissue grows on the surface of the implant and creates a permanent connection between the implant and human tissue [222]. This process depends on many factors, including the characteristics of the implant used (its quality, shape and materials), the condition of the bone tissue, surgical methods, procedures used, as well as the body’s ability to regenerate and heal [224].

The nanomaterials play a special role, being mainly used as the surface coatings, which allows to alter the topology (increased surface area of bone to coating connection) and bioactivity (improved recalcification and cell adhesion). Herein, we will discuss a few strategies employed for improving the above-mentioned parameters of the implant.

Using nanomaterials opens the possibility to optimize the topography of an implanted object by introducing hierarchical micro/nanostructures to the surface, which not only significantly increase the surface area but also enhances osseointegration. Ref. [225] A commonly used implant alloy (Ti–6Al–4V) was coated with ceramic, strontium substituted hardystonite (Sr-HT), hardystonite HT and classic hydroxyapatite HAp. A hierarchical topography was obtained via plasma spraying of HT or Sr-HT micron-sized powders onto the commercially available Ti alloy plates. As a result of the procedure the micron rough surface was covered with nanosized grains. Ref. [225] The coated plates were then evaluated in in vivo and in vitro tests. The in vitro analysis included the dissolution products of the coating in canine bone marrow mesenchymal stem cells (BMMSCs) culture environment, expression of adhesion-related and osteogenic-related genes of the BMMSCs, activity of the alkaline phosphatase (ALP) and calcium deposition assay. The in vivo tests conducted on femurs of the beagle dogs focused on detecting newly formed bone–implant connections and histomorphometric observation and biomechanical assay using the push-out test. The results demonstrated significant differences in favor of HT and Sr-HT-coated implants compared to the HAp ones. There was an improvement in cell adhesion and promotion of cell re-mineralization and new bone tissue formation, resulting in a possibility to consider the occurrence of a synergistic effect of hierarchical topography and dissolution products [225]. Not only the types of coatings researched but also different methods of developing a coated layer of an implant are tested. The nanocoatings based on calcium silicate (CS) are used to improve osseointegration [221]. The developed nanosheet-like structures of CS coating were used on Ti–6Al–4V implant alloy. The combination of two fabrication methods, atmosphere plasma spray (APS) and hydrothermal technology (HT), endowed the coating with desired properties such as high crystallinity and improvement in the degradation rate of the coating which is important for the storage and transportation of potential implants. The nanostructured CS coatings also possessed better apatite-mineralization, better cell attachment properties, enhanced cell proliferation, positively influenced osteogenic differentiation and increased expression of angiogenic factors compared to the traditionally obtained CS coating in the in vitro studies on the rat bone marrow stromal cells [221]. It is not necessary to coat the implant to obtain the micro/nano hierarchical topographic effect. The raw material of the implant can be machined in a way to obtain a developed surface on a micro and on a nanoscale. By treating the Ti–6Al–4V titanium alloy implants with the diluted solution of a hydrofluoric acid, the micro surface can be developed. Moreover, the process can be continued via anodisation of the material, resulting in the formation of the nanotubes, thereby creating a hierarchical micro/nano surface [220]. In the in vivo rat models, in comparison to naked, machined implants of the same titanium alloy, sophisticated implant surfaces are sufficient to improve the pull-out test outcomes, increase trabecular thickness and decrease trabecular separation from the implant surface, all in favor of micro/nano-modified implants and the micro-modified ones [220]. Facilitating the proliferation of the connective tissue cells and revascularization in the disturbed implant area may also contribute to better healing and integration of the implant. Taking this fact into consideration, a PS contained in a nanoformulation might improve the revascularization process and thus the healing of the surgical wound during photobiostimulation [226,227]. In a in vivo study conducted on mongrel dogs, their mandibular bone defects were treated with autogenous bone and satisfactory results in terms of new tissue formation and connection between bone and the implant were obtained. However, the liposomic solution and the nanoemulsion of the (PS) while activated with the LED light of an appropriate wavelength performed statistically better than the defects filled with blood clot alone [226]. Thus, further research on improving nanocarriers and PSs for photobiostimulation seems necessary.

## 9. Maxillofacial Surgery

Nanotechnology in maxillofacial surgery, despite intensive development in recent decades, has been still a relatively new branch of dentistry. Since the science of design, synthesis, characterization and application of materials and extremely small devices can be part of nanotechnology, its use in surgical practice can be very diversified. According to Nandagopal et al., three basic approaches of dental nanotechnology can be distinguished: (1) bottom-up approach—building particles by combining atomic elements; (2) top-down approach—using equipment to create mechanical nanoscale objects; and (3) regenerative nanotechnology—bio-mimicry [228]. Taking into account the benefits of using nanomedicine (such as advancement in the fields of drug delivery systems, gene therapies, body and organ imaging, surgical tools, and diagnostic procedures), it is obvious that it has found its use in dental surgery [229].

One of the areas of nanotechnology application in dental surgery is pain management, especially orofacial pain. Orofacial pain (OFP) can result from various physiological disorders and has very different etiology. Most common causes are related to temporomandibular disorders (TMDs), which include a number of clinical problems that involve the masticatory musculature, the temporomandibular joint (TMJ) or both. Trigeminal neuritis may also be an important factor triggering OFP. Its most important causes include injury secondary to dental procedures, dysfunction of the nervous system, neoplasias and/or infections. Neurovascular disorders can also cause chronic orofacial pain. Currently, the treatment of OFP consists of mono- or polytherapy containing non-pharmacological and pharmacological modalities. A multidisciplinary and multi-pronged approach seems to be crucial and beneficial for optimizing therapy [230]. A novel approach is the use of nanotechnology to eliminate or mitigate OFP, including OFP resulting from surgical procedures. For instance, vibrotactile devices (battery operated vibrators VibraJact, DentalVibe, Accupal) are operated on the principle of gate control theory through the simultaneous activation of nerve fibers via vibrations. Another pain control method used is the computer controlled local anesthesia system (CCLAD) (Wand^TM^/CompuDent^TM^ system) [231]. This technology allows for a modifiable and controlled method of administering the anesthetic preparation through light-weight hand-piece and foot control. An interesting topic, although still in the conceptual sphere, is the potential of anesthetic microrobots. The idea involves the use of a suspension containing nanobots that can reach the dentin and move in a specific direction to the dental pulp. The navigation factor could be, for example, a temperature gradient, chemical agents and/or a computer used externally by the dentist. After reaching the site of action, the nanobots would modify neurotransmission until the end of the medical procedure. Despite the increased opportunity for the development of such technological constructs since the discovery of the scanning tunneling microscope and the atomic force microscope, they have not been developed further [232].

One of the most interesting compounds from the point of view of nanotechnology in dental surgery is poly(L-lactic acid) (PLLA). PLLA is a safe and biodegradable material used for various applications, such as reversing signs of aging, treating facial fat loss, and serving as a scaffold in tissue engineering and drug delivery. Moreover, PLLA has been approved by the FDA for use in the reconstructive surgery of bone. It occurs in various numerous combinations, including hydroxyapatite and morphogenic protein 2 (BMP-2). A product based on magnesium–hydroxyapatite (HA) and human demineralized bone matrix under the trade name DBSint^®^ has been already approved for clinical use [233]. In two papers, Tavakol et al. also examined the possibility of using combinations of hydroxyapatite with other substances in surgical practice. In the first publication, nano-hydroxyapatite and hydroxyapatite/chitosan nanocomposites were used. The starting materials were obtained via in situ hybridization. Better results were observed for combinations characterized by smaller particle size, greater wettability and homogeneity. With these parameters, higher protein adsorption and cell differentiation and percentage of bone formation area were observed. At the same time, these data were confirmed empirically in an in vivo study on Wistar rats. The best parameters were obtained for pure nano-hydroxyapatite, while increasing the chitosan content worsened the regeneration potential of the system [234]. In the second paper, the group developed a material based on chitosan/nano-hydroxyapatite (CT/n-HAp) powder containing Ag and Si. The study aimed to find materials with a bactericidal or bacteriostatic component. Both physicochemical parameters of the material and biocompatibility were assessed. The model pathogen used in the study was *E. coli*. In this case, it was also confirmed that smaller particle size of CT/n-HAp/Ag (10.00 ± 0.09 nm) was associated with better cell viability and bacteriostatic activity compared to the CT/n-HAp/Si particles (18.00 ± 0.14 nm). At the same time, the hybridization of silver with the nanocomposite impaired the release of Ag^+^ ions. Despite this, the material was characterized with favorable parameters, such as high biocompatibility and bacteriostatic activity [235]. An in-depth analysis of the use of hydroxyapatite as a promising material for surgical use was carried out by Balhuc et al. They point to the enormous potential of this material, which has been intensively studied over the past 10 years. It should be emphasized that it can be used in very different areas of dentistry. Studies show the benefits of using it in oral implantology and bone reconstruction, as well as in restorative and preventive dentistry. Undoubtedly, based on the available literature, it can be indicated that HAnps have outstanding physical, chemical, mechanical and biological properties. On the other hand, the relatively optimistic results obtained so far have required extensive evaluation based on well-designed and randomized clinical trials [236].

An interesting material with potential application in dentistry was obtained by Wang et al. It was based on nano-TiO_2_ combined with medical silicone elastomer in various proportions. Silicone elastomer filled with 2% TiO_2_ nanoparticles results in a material with improved physical properties for the maxillofacial prostheses. At the same time, it is very important to note that in clinical practice, the addition of the nanomaterial significantly counteracted thermal aging of the material. It should be emphasized that currently the use of TiO_2_ in medicine is widely discussed in the world of science and there are no unambiguous opinions about its safety. Cytotoxicity tests were performed in the experiment, revealing that silicone elastomer filled with nano-TiO_2_ particles had short-term biocompatibility [237].

## 10. Orthodontics

Orthodontics is a branch of dentistry concerning the treatment of malocclusions. Correct bite is not only an esthetic issue but more importantly, it enables proper food grinding. What is interesting, malocclusions can be a primary cause of gastrointestinal problems. In orthodontic treatment, arch wires, power chains, elastomeric ligatures and many others are commonly used to move teeth [101,238]. Nanotechnology is garnering more attention to form new-shape memory materials (i.e., providing lower ion leaking), brackets (i.e., preventing caries formation) and ligatures (i.e., wearing resistance) with better properties.

**Figure 9:**
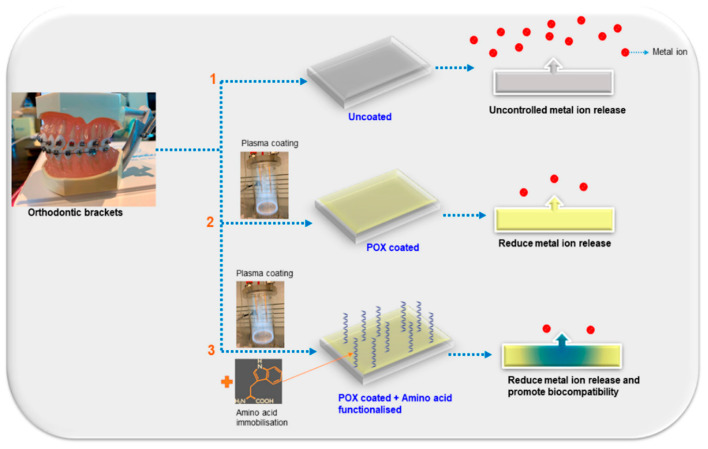
Schematic illustration of the design of surface-functionalized orthodontic brackets involving plasma coating and subsequent immobilization with enantiomers of amino acid to combat metal ion release [239] Reprinted with permission from Kumarasinghe et al., Coatings; published by MDPI, 2021.

It is well known that some metal ions could be toxic for humans and cause severe complications. Following long lasting orthodontic treatment the oral cavity is exposed to different metals (archwires, brackets, etc.). Therefore, Kumarasinghe et al. applied plasma-induced polymerization to coat orthodontic brackets to lower metal ion leak into the oral environment. Firstly, polyoxazoline was deposited on the brackets. Next, functionalization with tryptophan of the obtained surface coat was performed (Figure 9). These coated brackets showed only minimal metal ion leak in comparison to the untreated ones. Moreover, in the cytotoxicity assessment, decrease in viability of the fibroblast was not observed [239].

There are other tactics to limit ion release from arch wires. Especially, Ni ions leak from commonly used shape-memory arch wires—NiTi. Zhao et al. deposited a nanofilm consisting of TiO_2_ and HfO_2_ onto the NiTi alloy. Hafnium and many of its derivatives are highly biocompatible and possess anticorrosive properties. Therefore, improved wear-resistance was observed in comparison to the untreated NiTi arches. Modified wires reveal improved pseudoelastic abilities and keep its integrity under tensions [240].

The key issue in orthodontic treatment is maintaining the achieved occlusion. For this purpose, stainless steel retainers are commonly used. This causes long lasting metal ion leak.

Retainer based on glass fiber is available in the market. Khan et al. have proposed the modification of a glass fiber retainer with nano-hydroxyapatite and silanization to obtain a new retainer with more desirable properties. Firstly, the presence of hydroxyapatite enables the re-mineralization process but, more importantly, authors showed that the new fiber significantly hampered *S. aureus* and *C. albicans* adhesion. It should be pointed out that bonding strength of the obtained E-glass fiber modified with nHA is significantly lower in comparison to stainless steel retainers. Lower bonding property does not discourage the potential clinical use of the new retainer because its bonding strength is comparable with the clinically approved everStick Ortho^®^ fiber retainer [241].

Interestingly, it is estimated that more than 50% of force must be applied to move the teeth and overcome the frictional barrier.

In light of the above, Gracco et al. proposed modification of stainless steel wires with molybdenum disulfide and tungsten disulfide nanoparticles. These nanoparticles were attached to wires via electrodeposition. The experiments showed promising results according to which, in the “dry” model, the sliding was improved, whereas in the “wet” model, enhancement was only partial [242]. This finding paves the way for development of tools to minimalize forces (possible side effects and pain) and maximize the outcome of treatment.

Orthodontic appliances form hardly available spaces which have to be properly cleaned. There is a need to modify arch wires and other components (i.e., brackets, ligatures, etc.) with bactericidal agents providing self-cleaning tool.

Lin et al. have developed a coating of AISI 304 stainless steel used in dental appliances. This coating was composed of plasma-polymerized fluorocarbon layer bearing numerous nanoparticles. The obtained layer provided high hydrophobicity enabling self-cleaning and hampering bacterial attachment. Therefore, its use has been growing. Moreover, promising results were noted when modified stainless steel was treated with simulation of tooth brushing or peanut and nougat chewing. It turned out that under the aforementioned conditions, the layer was intact and did not lose its properties [243,244] less steel and NiTi arch wires were coated to prolong the release of bactericidal agents. Chlorhexidine hexametaphosphate nanoparticles were deposited on arch wires and pure chlorhexidine, a disinfectant commonly used as a working ingredient of mouthwash, was eluted. It was assessed that it kept on releasing for 28 days but no plateau level was achieved [245]. Metallic nanoparticles of well-known bactericidal activity are important silver nanoparticles. Thus, they were under investigation as well. NiTi arch wires were coated with silver nanoparticles via the electrodeposition method. Evaluation of this material showed that nickel ions release did not change but as a result of silver release, a ca. 1 log bacterial growth inhibition in the in vitro model was noted [246].

Self-cleaning or hampering of bacterial attachment to the surface is also important for removable appliances, which often have a rough top. Farhadian et al. have studied the impact of introduction of silver nanoparticles with a mean size 40 nm to the methacrylate resin of Hawley appliance in vivo. The authors have observed the significant reduction in *S. mutans* colony-forming units. The idea seems to be interesting but according to the authors, this study has limitations and further extensive experiments are needed. Especially, the impact of nanoparticles on mechanistic parameters, biocompatibility and bactericidal activity time should be assessed [247].

Orthodontic treatment can sometimes induce gingivitis and periodontitis as side effects of using a fixed appliance. In such a situation, intervention is necessary. A new approach to solve this problem is the Nano-Bio Fusion Gel (NanoCureTech, Gangdong-gu, Seoul, Republic of Korea), which has been studied in vivo. This aforementioned drug is a mixture of vitamins, sodium monofluorophosphate, and herbs extracts in a form of nanoemulsion. It was proven that within 90 days of use, this drug improved the following parameters: PI, papillary bleeding GI and PD [248].

**Figure 10:**
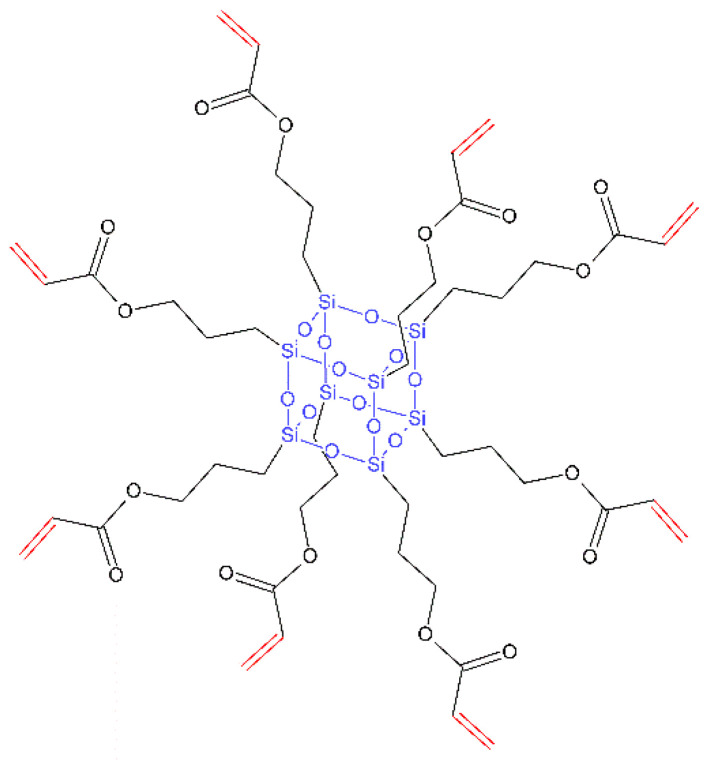
Chemical structure of APOSS: blue—nanocluster; red—bonds engaging in polymerization reaction with 2-octyl cyanoacrylate.

The bonding system not only enables formation of dentine seal interface, but it is very important in orthodontics as well to connect orthodontic brackets with the teeth. In order to develop a new orthodontic bonding system, nanoparticles have been introduced. Two commercially available adhesives, composite adhesive Transbond XT (3M, Unitek, Monrovia, CA, USA) and nanocomposite adhesive Filtek Z350 XT (3M, ESPE, St. Paul, MN, USA), were compared. Authors have concluded that tensile bond strength of the nanofilled adhesives achieved much lower values than the reference, but it is still sufficient for clinical use. However, the adhesive remnant index (ARI) indicated that nanofilled bond may decrease the risk of enamel destruction [249]. Scientists are continually looking for a new orthodontic bond with better properties. Fadaie et al. have tried to use non-toxic and poorly irritating tissues, 2-cyanoacrylate. Cyanoacrylates are well-known compounds with dental application (periodontitis and oral surgery). Although the bond strength of cyanoacrylates is sufficient for binding orthodontic bracket to the tooth, performed studies indicated that bonding strength dramatically dropped during long-term tests. Therefore, authors have introduced silica-based nanocluster with linkers enabling linking to the polymer net (APOSS, Figure 10) into the polyacrylate bond. It has been observed with the shrinkage of the newly obtained composite bond, amount of APOSS increased up to 20 %wt., and then they have noticed no changes in the shrinkage strain. The observed phenomenon was connected with net cross-linking by APOSS in higher concentrations. Moreover, the flexural strength increased with increasing APOSS concentrations up to 20 %wt. as well as micro shear bond strength. The new bond for orthodontic applications also revealed lower water sorption and resistance to hydrolytic decomposition [250].

Formation of enamel white spots during long-lasting orthodontic treatment is very common, the cause of which is linked with the demineralization process of enamel.

Many attempts have been made to solve this situation. Modified orthodontic adhesives with re-mineralization activity were introduced; nevertheless, the demineralization process has not been stopped. Therefore, researchers have started introducing nanotechnology to obtain a new generation of orthodontic adhesives. An interesting approach is the modification of commercial adhesive Transbond XT (3M Unitek, Monrovia, CA, USA) with nanoparticles of amorphous calcium phosphate (NACP). Such a mixture with up to 5% of NACP has no impact on shear bond strength. Simultaneously, higher mineralization around brackets bounded with modified adhesive was observed in comparison to the control. Nevertheless, slight loss of enamel minerals around and below the brackets in comparison to bracket-free enamel was observed. After six months, a decrease in *S. mutans* colonies was observed in comparison to the control. It probably resulted from the increasing pH caused by the release of calcium ions and phosphates [251] Liu et al. have proposed a solution to overcome the problems of enamel white spot formation around the teeth-bounded brackets. They have developed a novel adhesive based on 2-methacryloxylethyl dodecyl methyl ammonium bromide (MAE-DB), a polymerizable quaternary ammonium salt with antibacterial activity. Moreover, NACP has been added to enable the enamel re-mineralization process in the resin. Following the experiments, the authors concluded that in comparison to commercial orthodontic adhesives, these modified materials with 5% MAE-DB and 40% of NACP do not change with increasing bond strength of the brackets. Importantly, the presence of MEB-DB significantly hamper the growth of *S. mutans* biofilm on the resin. However, simultaneously, NACP is a reservoir of calcium ions and phosphate that are required in the re-mineralization process [252]. Antibacterial acrylic resin as an orthodontic adhesive based on nano-ZnO has been proposed as well. The obtained adhesive showed its mechanistic parameters including shear bond strength at the same level as adhesives without nanoparticles and the commercially available ones [253]. The tests were also performed with commercial silver-modified nanoparticle adhesives. Eslamian et al. introduced silver particles with a mean size of 50 nm into Transbond XT^®^ (3M Unitek, CA, USA) via direct mixing before use. The modification resulted in significantly reduced viability of *S. mutans*. Unfortunately, nano-adhesive also decreased shear bond strength. It should be pointed out that the value of this parameter stayed at an acceptable level [254] Yi et al. decided to prepare orthodontic adhesives containing nano-CaF_2_ (58 nm) to improve the release–recharge parameters. They have used PEHB resin as a base. The best parameters were observed for resin loaded with 30% nano-CaF_2_. The fluoride release lasted much longer and achieved higher concentrations (about 80%) than the commercial resin. Moreover, a solution of NaF, commonly present in mouth-rinses, was used as a recharge fluid. After the resin was recharged with fluoride, its re-release was observed which lasted one week. Authors also noted relatively good biocompatibility and shear bond strength similar to the control resin [255]. Studies on the mechanical properties of tooth enamel after brackets bonding–debonding have been performed. The authors used 20 human molars divided into two groups. In the first group, the teeth were bonded to ceramic brackets (Clarity Metal-Reinforced 6400-823, 3M Unitek, Monrovia, CA, USA) with a commercial composite adhesives (Transbond XT, 3M Unitek, USA). In the second group, a commercial nanocomposite adhesive (Filtek Z350 XT, 3M ESPE, Irvine, CA, USA) was used as a bond. Sharp-edged pliers were applied for brackets debonding. The results pointed out that the nano adhesive system did not increase the mechanical properties of enamel after the debonding procedure in comparison with the non-nano bonding system. After debonding, enamel porosity increased and formation of micro cracks was noted for both groups. Authors have linked the decrease in mechanical properties of enamel with the debonding procedure, and not with the adhesive system used [256]. One of the possible solution of the problem is the development of a proper primer. Xu et al. prepared a primer containing fluorinated calcium phosphate nanoparticles. After investigations, it became clear that the primer with 35 wt.% nanoparticles efficiently prevented formation of white spots [257].

## 11. Nanoparticles vs. Oral Bacteria

Bacterial presence in oral cavity leads to problems mainly associated with biofilm formed by some species [258,259] lm constitutes a solid resistant barrier that makes bacteria more resistant to various antimicrobial treatment, making it much more difficult to eliminate [259,260] chanisms that could be associated with the increased resistance are reduced penetration of drugs, inactivation of the antimicrobial agent, or the existence of bacteria in the slow growing state [228,261] evertheless, the presence of bacterial biofilm can induce dental caries and periodontitis. Caries is related to tooth demineralization induced by fermentation of carbohydrates into acids. Acidification of the environment influences the bacterial composition, leading to low-pH sensitivity of teeth [262,263,264]. On the other hand, periodontitis is associated with gum inflammation due to excess accumulation of plaque. This can lead to destruction of tissues and further to the loss of alveolar bone around the teeth, therefore causing their loss [262] Qiao et al. investigated the safety and the photodynamic effect of PACT on two cell types critical for wound healing of periodontal tissues, human periodontal ligament cells (hPDLCs) and human gingival fibroblasts (hFBs) in vitro and other mechanisms to promote the healing of periodontal tissue. They evaluated the proliferation of hPDLCs and hFBs and cell attachment on cementum slices of hPDLCs and hFBs via the MTT assay. It turned out that PACT treatment induced a strong time-dependent increase in hPDLC and hFB proliferation at 24 h, 72 h and 6 days. Similarly, PACT also promoted time-dependent attachment of hPDLCs and hFBs on the cementum slices at all time points compared to the controlled cells. Type I collagen synthesis of hPDLCs and hFBs was significantly stimulated via PACT in a time-dependent manner. A marked increase in the specific ALP activity in hPDLCs was observed. PACT not only exhibited no cytotoxicity to hPDLCs or hFBs, but also stimulated proliferation, attachment and collagen synthesis of hPDLCs and hFBs and ALP activity of hPDLCs [265].

Bacteria are one of the main causal factors in many diseases, such as periodontal disease and caries, but the pathogenic effect is in fact a result of complex interactions among their presence and health conditions of the patient. Their population needs to be controlled in order to prevent disease development [262,263]. As with peri-implantitis, dealing with bacterial biofilms includes similar methods, such as mechanical removal, PACT, and the use of antiseptics or antibacterial agents. Great effort was put into the development of PACT-mediated treatment and various PSs were tested, and despite the activity potential of the method, it still requires further research, as plaque reduction often appears insufficient for medical applications. As bacterial growth is rather logarithmic, reduction by 90% or even 99% has no effect; the bacterial population still can be rebuilt relatively quickly [263]. Therefore, the accepted threshold for sufficient antibacterial activity is 99.9% (3 log) reduction. Soukos et al. showed potentially good activity of chlorin e6, modified with lysine pentamer (pL-Ce6) against bacteria present in human subgingival plaque, in the presence of 662 nm light. Employment of pulsed photomechanical wave increased the activity up to 2 log (<1 log without pulses), due to enhancement in drug uptake [262] Müller et al. compared methylene blue (MB)-mediated PACT to ozone and use of antiseptics (chlorhexidine and sodium hypochlorite) against multiple-species biofilm. They indicated that ozone, PACT and 0.2% chlorhexidine showed no activity. In contrast, 2% chlorhexidine and 0.5% sodium hypochlorite showed 1 log reduction, whereas 5% sodium hypochlorite removed the plaque completely [263]. MB became a popular PS and many other studies presented its effects, although its activity was rather low towards oral bacteria biofilms (<1 log) [266,267] Iniz et al. pointed that despite insufficient activity, MB-mediated PACT remains safe for dental pulp. Another popular PS, Toluidine blue O, according to Lima et al., exerts good antibacterial effect (up to 5 log) on bacteria involved in dental caries, which can contribute to clinical applications [268]. Another study by Lin et al. was focused on the treatment of oral wound infections in a rat model and it showed significantly lower activity, up to 1.5 log [269] Similar to Lin et al., Ichinose-Tsuno reported approx. 1.4 log reduction with Toluidine blue O in a human model, and pointed that PACT may decrease fibroblasts viability, similar to standard antiseptics (hydrogen peroxide and benzalkonium chloride) [270] Asnaashari et al. investigated the influence of light source on Toluidine blue O activity. Both diode laser and LED lamp showed significant reduction in *E. faecalis* populations (~5.5 log and ~6 log, respectively) [271]. Erythrosine also appears to be useful in PACT. Chen et al. showed that when combined with chitosan nanoparticles, erythrosine showed up to 4.5 log reduction in *S. mutans* and up to 3.5 log for *C. albicans* biofilms, whereas for *P. aeruginosa* biofilm, the reduction was only 2 log, indicating that Gram-negative species are more resistant to this kind of therapy [272]. The effect of erythrosine in comparison with Rose Bengal against bacterial biofilms was also investigated by Pereira et al., indicating its potential. However, the obtained reductions were not significant for clinical applications (<1 log) [273].

Silver, widely known for its antimicrobial properties, was also incorporated into biofilm control. Besinis et al. compared silver nanoparticles coated onto dentine, to chlorhexidine and silver nitrate towards *S. mutans* biofilm. They showed up to 1.3 log reduction for both silver nitrate and silver nanoparticles, whereas 1% (*v*/*v*) chlorhexidine showed an insignificant effect (<1 log) [274] Marsich et al. utilized silver nanoparticles bonded electrostatically with lactose-modified chitosan doped onto alginate–hydroxyapatite scaffolds. Those systems revealed good inhibition of several bacterial species (>3 log), although there was an exception (~2 log) [275] Amazanzadeh et al. tested other antibacterial materials based on ZnO and CuO, and indicated that ZnO showed an insignificant reduction in bacterial viability, although CuO either alone or combined with ZnO showed rapid bacterial reduction [276].

## 12. Nanoparticles vs. Oral Cancer

There are several types of oral cancers, but more than 90% of all oral neoplasms are squamous cell carcinomas, and therefore the term oral cancer tends to be used interchangeably with oral squamous cell carcinoma (OSCC) [277]. Oral cancers are part of a group of cancers referred to as head and neck cancers (HNCs). They are routinely discovered late in their development, which makes them particularly dangerous and thus are associated with low survival rate. In the early stages, patients do not present pain or symptoms that they might easily recognize. The advent of HPV16 virus is another obstacle in the early diagnosis of oral cancers and it mainly contributes to their incidence rate. This virus prevents production of visible lesions or discolorations in the oropharynx; the tonsils and the base of the tongue are the areas of the mouth where the early warning signs of the disease process are present. Oral cancer is often discovered after its metastasis to another location, such as the lymph nodes of the neck, which makes the prognosis significantly worse than for a cancer localized in the intraoral area only. At the later stages, the local structures are subject to deep cancer invasion [278]. A role in the initiation of OSCC has been also attributed to other viruses such as *Herpes simplex virus* (HSV), *Epstein–Barr Virus* (EBV), *human herpesvirus-8* (HHV-8) and *cytomegalovirus*. Moreover, there have been some reports indicating a link between *Candida* infections and OSCC onset [279].

PDT has been used in the curative and palliative treatment of OSCC.

Basically, porfimer sodium (Photofrin) and hematoporphyrin derivative (HpD/Photofrin) as well as the second-generation synthetic drug mTHPC (Foscan) are used for the treatment of head and neck cancers. Photofrin has relatively poor tumor selectivity and limited absorption of red light with good drug penetration. New-generation chlorin-based PSs are developed to improve selectivity and reduce the exposure time and photosensitivity during administration. The chlorine e6 derivative (Photolon) is becoming more and more popular for superficial and deep lesions in the head and neck area, contributing to a shorter period of post-treatment photosensitivity. Systemically administered PSs, such as mTHPC, can be applied in premalignant lesions of both early-invasive and untreatable carcinomas of the oropharyngeal region [280]. The comparison of the efficiency of PDT treatment and surgery in oral cavity tumors has continued. The efficiency of PDT treatment of primary T1 (of the smallest size) and T2 tumors (more than 2 cm but not more than 5 cm across) was compared with surgery. In case of the treatment of T1 tumors of the oral cavity via either mTHPC-mediated PDT or transoral surgery, the outcomes seemed similar. For T2 tumors, surgery appeared to be more effective. Both approaches resulted in similar overall survival rates for the T1 and T2 tumors. Therefore, there are suggestions that PDT could be a primary treatment option without some of the disadvantages associated with surgery, such as speech and swallowing impairment and poor aesthetic results. However, effective light penetration for mTHPC-mediated PDT is approximately 10 mm at 652 nm, which limits curative treatment with surface illumination to tumors with a ≤5 mm invasion depth [281] Karakullukcu et al. confirmed the comparability of the surgical approach and mTHPC-mediated PDT in terms of early-stage oral cavity cancer control and survival to trans-oral resection [282] Anand et al. developed an approach based on PDT, in which 5-ALA (a precursor for PPIX) was administered before irradiation to test its efficiency in treating non-melanoma skin cancers (NMSCs), including squamous cell carcinoma (SCC). They carried out studies on mice that were administered natural dietary vitamin D_3_ (cholecalciferol) prior to PDT to improve PPIX accumulation and the anti-tumor effect of PDT. On the 11th day of the dietary regimen or on the 4th day of the systemic regimen, ALA was administered intraperitoneally for 4 h. At this time, mice were either sacrificed for tumor and skin harvest or tumors were exposed to red light (633 nm) using a non-coherent light source at a fluence of 100 J/cm^2^. Ten days of vitamin D3 oral supplementation raised three to four levels of PPIX and caused a 20-fold enhancement in PDT-mediated cell death in subcutaneous A431 tumors. Minimal hypercalcemic risk in serum of animals was observed. The researchers came to a conclusion that such an administration of cholecalciferol can render ALA-PDT to be a safe neo-adjuvant and it could be used for cancer pretreatment before PDT for human patients [283] Bhuvaneswari et al. evaluated the efficacy of PDT in combination with an epidermal growth factor receptor (EGFR) inhibitor, nimotuzumab, in oral cancer cell lines and an OSCC xenograft tumor model. The scientists demonstrated that EGFR inhibitors, nimotuzumab and cetuximab, impeded the migration and invasion of oral cancer cell lines and human endothelial cells. They applied intravenously chlorin e6 (Ce6), a second-generation PS with nimotuzumab and cetuximab, and later they irradiated tumors with a laser light at a wavelength of 665 nm (light dose of 150 J/cm^2^). The combination of PDT and monoclonal antibodies resulted in a reduction in cell proliferation in different oral cancer and endothelial cells. Moreover, the combination therapy in in vivo studies on mice synergistically delayed tumor growth in comparison to control and PDT-only treated tumors. The anti-tumor activity of the combination therapy involves the reduced expression of EGFR, proliferation marker Ki-67, and the cluster of differentiation CD31, which provides a rationale for future clinical investigations [284] The epidermal growth factor receptor (EGFR) is highly expressed in OSCC and drugs blocking EGFR proteins are used in cancer therapy. He et al. investigated in vivo and in vitro the administration of an EGFR inhibitor, nimotuzumab, after treatment with aminolevulinic acid (ALA)-PDT. For in vitro irradiation, He-Ne ion laser was operated at a wavelength of 635 nm. A light power density of 0.2 W/cm^2^, an illumination time of 50 s, and a light energy density of 10 J/cm^2^ were used. Two cell lines, CAL-27 and SCC-25, were subjected to the combination procedure and in both cases, intracellular ROS generation increased. However, the pretreatment with ALA-PDT combined with nimotuzumab did not essentially increase ROS generation in both CAL-27 and SCC-25 cells in comparison with the ALA-PDT group. The in vivo tests were performed on mice with induced tumors. The animals were divided into four groups, including control (PBS), ALA-PDT (200 mg/kg), nimotuzumab (1 mg/mouse), and ALAPDT + nimotuzumab. The protocol for light stimulation of PDT was 108 J/cm^2^ for 540 s. Mice in the ALA-PDT group received peritoneal injection with ALA (2 h later) followed by irradiation. Western blotting was carried out to compare EGFR expression in the different groups. The scientists observed an enhanced inhibition of OSCC cell growth in vitro and in vivo as a result of the combined treatment with nimotuzumab and ALA-PDT and this approach seems to contribute to an increase in the cure rate in OSCC without increasing toxicity [285].

However, apart from PDT, for oral cancer treatment, much effort must be spent to develop various nanoparticles that can provide targeted delivery of chemotherapeutic agents and can enhance cellular uptake. The use of nanoparticles enables to load low concentrations of the chemotherapeutic drugs, which reduces their adverse effects.

**Figure 11:**
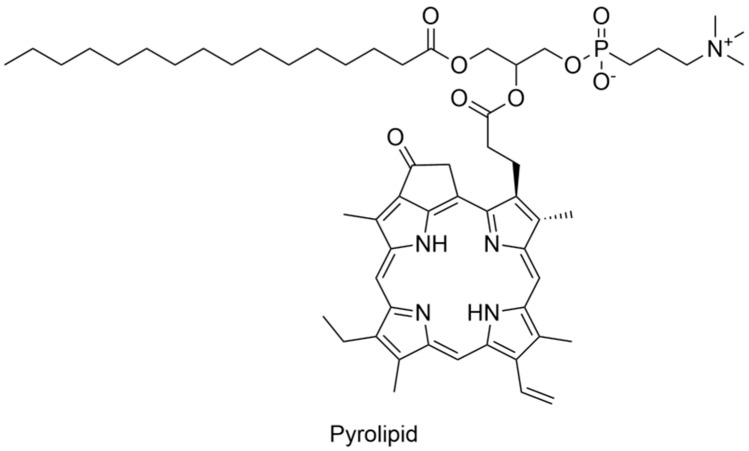
Structure of pyrolipid PS.

Chen et al. investigated the therapeutic effect of topical photosan–PDT and developed anisamide-targeted lipid–calcium–phosphate (LCP) nanoparticles to deliver encapsulated HIF1α siRNA to the tumor cells of oral squamous cell carcinoma. They intended to demonstrate the enhancement in the cell-killing efficacy of topical photosan–PDT as a result of the inhibition of HIF1α expression by siRNA nanoparticles. They performed experiments in both cultured cells and xenograft tumors in nude mice. They used PS Photosan, which is converted intracellularly into the active form, protoporphyrin IX (PpIX), that produces reactive oxygen after irradiation with a 630 nm red light. HIF1α plays a key role in the regulation of apoptosis and cell cycle progression. Calcium phosphate (CaP) binds nucleic acids with high affinity and dissolves in the acidic pH of the endosome. After increasing osmotic pressure, the endosome ruptures and its cargo is released into the cytoplasm. CaP cores were stabilized using dioleoylphosphatydic acid (DOPA) and then coated with a cationic lipid. The target of the combination therapy is the cytoplasm of the sigma receptor-expressing human squamous cell carcinoma. Significant siRNA accumulation and reduced HIF1α expression both in human SCC4 cells or SAS-xenografted mice were observed. The combination of nanoparticles with PS led to a 40% decrease in OSCC tumor volume after 10 days. Combination therapy turned out to be more effective than either HIF1α siRNA or PDT alone and resulted in an effective reduction in HIF1α expression, increased cell death, and significantly inhibited cell growth [286] He at al. developed a nanoformulation for combined chemotherapy and PDT to achieve synergistic effects. They designed nanoscale coordination polymer (NCP)-based core–shell nanoparticles loaded with *cis*-platin and PS pyrolipid (pyropheophorbide bonded to a lipid, Figure 11).

The delivery system NCP@pyrolipid released the drug and PS to cause cancer cell apoptosis and necrosis. The scientists investigated the novel formulation in the *cis*-platin-resistant human head and neck cancer SQ20B xenograft murine model and its efficiency is compared with that of monotherapy. LED-light irradiation with a total light dose of 180 J/cm^2^ was applied to excite PS. The outcomes of the in vivo pharmacokinetic and biodistribution studies revealed prolonged blood circulation times, low uptake in organs, and high tumor accumulation of *cis*-platin and pyrolipid. Tumor volume was reduced in 83% at low drug doses. NCP@pyrolipid turned out to be able to carry high loadings of *cis*-platin and pyrolipid and after activation it could release the payloads at the target locations. The nanoformulation reduced *cis*-platin and pyrolipid efflux due to cell membrane-modification by pyrolipid. NCP@pyrolipid avoided mononuclear phagocyte system uptake, which led to prolonged systemic circulation after intravenous injection for enhanced and specific tumor accumulation, thanks to the EPR effect [287] Zhao et al. designed a monomeric self-assembled nucleoside nanoparticles (SNNP) loaded with 5-fluorouracile (5-FU) to obtain 5-FU-SNNP and applied it against oral cancer. They formulated such a novel nanoparticle drug platform to overcome the disadvantages of conventional oligonucleotide-based drug delivery systems, such as long synthetic protocols, high cost, and poor chemical or enzymatic stability. Furthermore, they intended to tackle the limitations of 5-FU, such as short biological half-life and toxic side effects. As individual nucleosides cannot form stable nanostructures in an aqueous solution, the scientists had to develop a monomeric SNNP for a drug delivery system with the expected properties. They used a L-configurational pyrimido [4,5-d]pyrimidine nucleoside building block to form stable nanoparticles in one step using water as the sole solvent. The anticancer efficiency of 5-FU-SNNP was tested in a mouse xenograft model of OSCC and significant inhibition of the tumor growth compared with free 5-FU was noted. Noteworthy, SNNP alone showed no anti-tumor effect [288] Wang et al. prepared gold nanorods (GNRs) conjugated with Rose Bengal (RB) molecules to investigate their usefulness for in vivo photodynamic and photothermal oral cancer therapies. The evaluation was performed on hamsters with induced oral carcinoma. The cell viability in vitro was examined in human oral squamous cell carcinoma cell line (CAL-27). The developed formulation of RB-GNRs efficiently produced singlet oxygen after irradiation with 532 nm green light and revealed high photothermal efficiency under 810 nm of near-infrared irradiation. After the combined PDT-PTT treatment, the tumors were nearly cleared with the preservation of normal tissues and the treatment could be repeated as often as necessary. The RB-GNRs combined with the capabilities of PDT-PTT turned out to be more efficient against oral cancer than the single PDT or photothermal therapy (PTT). Thanks to the specificity of RB to oral cells, RBGNRs accumulate preferentially in the cancer cells to overcome the nonspecific delivery of heat by GNRs while sparing the normal cells. The enhanced PDT efficacy of RB-GNRs resulted from the increased uptake of RB by the cancer cells. The studied PDT-PTT treatment was superior to conventional treatments in terms of improved organ functions, cosmetic appearance, cytocompatibility and presented promising potential in cancer treatment strategies [289] Wang et al. studied the possibility of using a special nanoformulation for oral cancer treatment. They developed novel nanostructured materials composed of chitosan/tripolyphosphate (CS-TPP) loaded with ALA and methylenetetrahydrofolate dehydrogenase 1-like (MTHFD1L) shRNA, a tumor-associated cytokine, to assess the efficacy of the simultaneous delivery of shRNA/PS on the gene expression of oral squamous cell carcinoma (OSCC) cells. Therapeutic trials were performed in vivo on mice. The tumor volume was measured and it turned out that it grew much slower due to stimulating tumor cell apoptosis in the nanoparticles-mediated combination therapy in comparison to the control. The application of nanoparticles appeared to improve gene therapy and ALA-PDT efficiency. CS-TPP-shMTHFD1L-ALA-PDT could promote ROS accumulation in OSCC cells, which can result in mitochondrial dysfunction. The developed co-delivery system seemed to be a promising candidate for PS/gene delivery [290].

The crucial determinant of successful cancer treatment is its detection at an early stage. Nanotechnology also brings potential solutions to this problem, applicable in the dentist’s office during routine visit.

Yang et al. worked on novel methods of detecting oral cancer, which is difficult to diagnose in the early stages. They designed a high performance nanoparticle for the photodynamic detection (PDD) of oral cancer. They formulated a system consisting of a negatively charged polymer succinate-modified chitosan (SCHI) complexed with folic acid-modified chitosan. Chitosan is a biocompatible and biodegradable polymer with antibacterial properties and the ability to easily approach the cell membrane. Nanoparticles were expected to have a high drug (ALA) loading efficiency and good drug release in the cellular lysosome. The nanoparticles were taken up by oral cancer cells via folate receptor-mediated endocytosis. The release of ALA in the lysosome was promoted by the reduced attraction intensity between chitosan and the PS because of the deprotonated SCHI molecules, resulting in a higher accumulation of intracellular PPIX for photodynamic detection. This N-succinyl-chitosan-incorporated and folic-acid-conjugated chitosan nanoparticles turned out to be a promising system for oral-specific delivery of ALA for fluorescent endoscopic detection [291].

PDT is also effective in pre-cancerous lesions as Romeo et al. reported. They evaluated the effectiveness of topical aminolevulinic acid (ALA-PDT) in the treatment of oral proliferative verrucous leukoplakia, which is considered to be the cause of about 60% of oral cancers. Complete response of the lesion was observed after four sessions of PDT and no recurrence appeared after one year [292].

## 13. Personal Care

Simultaneously, with the growth of common knowledge about dental health the need for high-quality personal care products that are both innovative and more importantly functional rose significantly. The key factor is also the rising demand for tooth aesthetics. All of these challenges might be met with a little help of nanostructures such as nano-hydroxyapatite (*n*-HA), nano-carbonate apatite (*n*-CAP), nano-carbonate substituted hydroxyapatite (nano-CHA), nano-fluorhydroxyapatite (*n*-FA), and nano-sodium trimetaphosphate (TMP) [12,13,14,15,16,17,18]. Dentition nanostructures, such as tea polyphenol-modified calcium phosphate nanoparticles (TP-CaP), nano silver fluoride (NSF) and silver diamine fluoride nanoparticles (SDF), are being researched as potential anticaries and antimicrobial agents that can additionally prevent enamel erosion and promote its re-mineralization [293,294,295].

Dentine hypersensitivity is a condition when the dentine tubules—structures connecting pulp and oral cavity—are exposed due to a lesion in the tooth surface. Its characteristic symptom is a sharp pain occurring when dentine is subjected to stimulus of a certain character, such as chemical, tactile, thermal, or osmotic [296] he nano-apatite particles (mentioned above) have the ability to adhere to the surface of tooth enamel and due to their nanosize, they can penetrate the dental tubules. Moreover, their biomimetic properties allow them to grow apatite crystals of similar nanostructure to the one present in natural enamel, thus letting them integrate [13,14]. This phenomenon results in the desensitizing ability of nanostructures used in toothpastes, wherein they occlude the exposed tubules and promote lesion re-mineralization. A study showed that during an eight-week-long treatment, subjects cured with a dentifrice containing CHA nanocrystals compared to subjects treated with a conventional method containing sodium fluoride/potassium nitrate demonstrated significantly greater improvement in sensitivity tests, i.e., airblast test [18].

The biointegration abilities promote re-mineralization of the enamel which may be crucial in combination with the fluoride’s abilities, such as hardening the enamel and preventing tooth decay [297]. study showed that TMP nanoparticles combined with the fluoride-containing toothpaste indicated a synergistic effect causing more fluoride to integrate into enamel thus preventing caries more efficiently [13]. The increase in effective re-mineralization caused by the application of TMP coupled with fluoride formulation may allow to decrease fluoride concentrations in commercially available personal care products thus decreasing the risk of fluorosis. Another nanostructure promoting re-mineralization and fluoride embedding into enamel is *n*-HA. An oral rinse and a toothpaste containing *n*-HA and fluoride—demineralizing agents—were used in a study to protect the enamel against acid. Individually, *n*-HA and fluoride presented significant re-mineralizing properties—the fluoride being a stronger re-mineralizing agent—but only the combined application of both *n*-HA and fluoride displayed the best results in the prevention of demineralization of the studied tooth. Moreover, it led to the formation of fluoroapatite which is known to be more acid-resistant than standard hydroxyapatite [298].

It is well known that teeth bleaching leads to tooth surface loss and is responsible for evoking hypersensitivity of the dentition; at the same time, bleaching is not permanent and repetitions of the procedure are required to maintain the desired effect [299] hitening is widely used whether at home or under the supervision of dental personnel in office which might have side effects affecting the comfort level of patients. To decrease the intensity and the occurrence of side effects, such as tooth hypersensitivity and enamel decolorization, nanomaterials may be applied in the bleaching solutions or in the after-treatment dentifrice as a counteragent. An in vitro study on bovine specimens indicated that 10% solution of the *n*-CAP used after bleaching showed promising properties of recovering damaged enamel structures and maintaining color of the tooth after bleaching [16]. Also, a clinical trial reports that the addition of 2% nano-hydroxyapatite to a 6% hydrogen peroxide bleaching gel can help reduce the side effects of bleaching after-treatment, i.e., tooth hypersensitivity. However, in comparison to the results produced by a gel without *n*-HA, the addition of 2% *n*-HA to the bleaching gel did not significantly impact the change in the tooth shade even after nine month follow-up. The results of both groups were comparable [15].

Dental caries is termed as a chronic illness of childhood, called the “silent epidemic” that mostly affects the young, the poor and minorities, especially in less privileged countries [297]. Being a worldwide problem, dental decay generates a fair amount of healthcare costs. Therefore, it is necessary to search for more effective and highly efficient strategies against it. Nano silver fluoride (NSF), containing chitosan silver nanoparticles with the addition of fluoride, is being researched as an anticaries agent and it promises a new approach to tackle children’s dental decay. A clinical double-blind trial reports that a solution of NSF is capable of successfully arresting active dental caries. Having the additional advantage of not staining the surface of a treated tooth and more importantly not requiring a clinical setting, this non-invasive strategy might be suitable for treating primary tooth decay in children, especially from poor communities [295]. Another advantage of NSF is its low cytotoxicity compared to other conventionally used agents for treating caries, such as, chlorhexidine and silver diamine fluoride [294].

## 14. Summary

**Figure 12:**
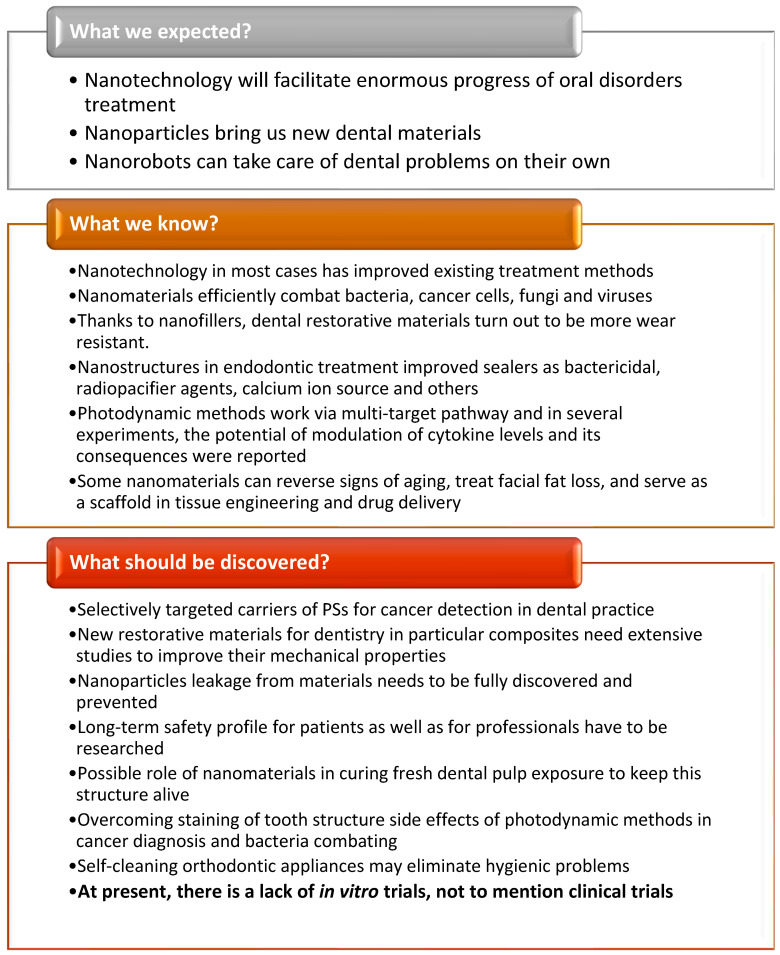
Summary of outlooks.

**Figure 13:**
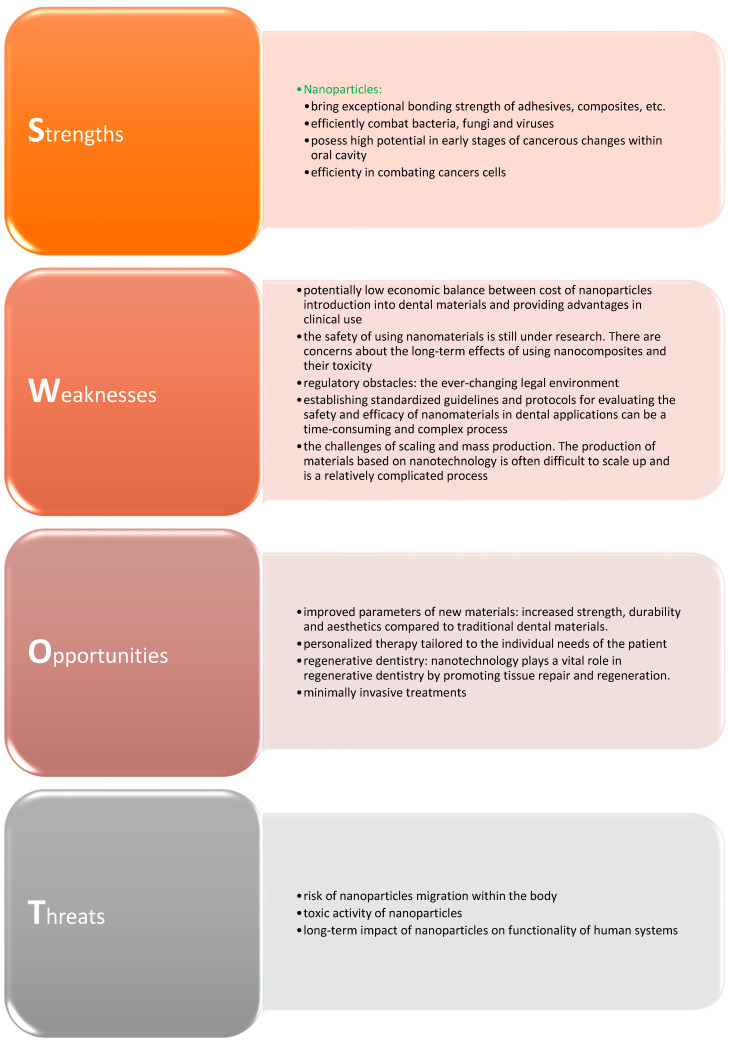
SWOT analysis.

Nanomaterials are becoming increasingly popular due to its potential use in a wide range of areas, including clinical dentistry. Nanotechnology has completely altered how dental materials interact and behave with oral tissues, which is proven by the development of antimicrobial dental adhesives, cosmetic restorative materials based on nanoparticles, surface decoration for dental implants, and high-strength denture bases. Numerous investigations for therapeutic dental applications, such as fluoride release and medication administration, have now been conducted due to a better knowledge of materials and oral tissues’ contact at the nanoscale. The primary objective of the proposed overview is to highlight the current advancements in the therapeutic uses of nanoparticles in dentistry. Far-reaching future perspectives have been outlined by Sreenivasalu et al. They pointed out the important role that will be played by 3D printing, nanobots and nanozymes [300]. It is undoubtedly true that nanotechnology has improved several conventional approaches that are being used currently in dentistry. Continuation of the above-mentioned studies may solve many problems that persist in the present dental treatment scenario. In restorative dentistry, one of the biggest problem to overcome is unsealing the tooth seal. This has opened a lot of possibilities to prepare new composites and adhesives to provide a strong connection between the seal and tooth structures. Another significant challenge is choosing a composite color. Though there are many available dental materials that are commonly used and producers declare them to be color-fitting, they are not perfect. Much work is required in order to provide a really smart-colored self-fitting filling. On the other hand, it is well-known that change in the color of ones teeth is dependent on nutrition and environmental factors. The best solution may be to use nanotechnology to simultaneously change the color of the tooth structures. Endodontics is the second largest area for nanotechnology implementation. The ongoing research has to be intensified to obtain a root canal sealant with high strength. It is extraordinarily important to develop a sealant with permanent antibacterial activity, which is available not only within the seal but also in the dentine around it. Such an approach may eliminate the risk of re-endodontics treatment. In orthodontics, one of the key factors determining the success of the therapy is the constant force applied to teeth in the required direction. The material used needs to be changed in particular intervals to maintain the movement of teeth. Application of nanotechnology will help the material retain its mechanic properties for a much longer period. Simultaneously, nanotechnology may provide self-cleaning surfaces to achieve good teeth health during orthodontic treatment. Another significant occurrence is the formation of white spots after debonding brackets. Therefore, intensifying the development of new orthodontic adhesives based on nanomaterials is highly desirable. The unsolved dental problems of patients result in decreasing bone strength, eventually leading to loss of teeth. Moreover, in the treatment of periodontitis as well, nanotechnology is garnering the attention of researchers. The summary of outlooks is presented in Figure 12, and the SWOT analysis is shown in Figure 13.

## Figures and Tables

**Table 1:** Flexural strength and modulus of some commercial dental restorative materials.

Ref.	Name	Manufacturer	Flexural Strength [MPa]	Flexural Modulus [GPa]
[44]	Z250	Filtek	136.40	11.53
[45]	Hybrid resin composite	TPH	119.15	4.90
[46]	Artglass	Heraeus Kulzer	114.00	13.00
[46]	Concept	Ivoclar	109.00	15.00
[45]	Compomer	Dyract	91.49	6.17
[45]	Resin modified glass ionomer	Vitremer	39.36	5.57
[45]	Glass ionomer	Ketac-Bond	17.02	5.20

## Data Availability

No new data were created or analyzed in this study. Data sharing is not applicable to this article.

## References

[1] Damodharan J. (2021). Nanomaterials in Medicine—Overview. Mater. Today Proc..

[2] Glowacka-Sobotta A.A., Ziental B.D., Sobotta C.L. (2021). Chapter 12 Porphyrinoids Used for Photodynamic Inactivation against Bacteria. Applications of Porphyrinoids as Functional Materials.

[3] Wu Q., Miao W., Zhang Y., Gao H., Hui D. (2020). Mechanical Properties of Nanomaterials: A Review. Nanotechnol. Rev..

[4] Saxena S.K., Nyodu R., Kumar S., Maurya V.K., Saxena S.K., Khurana S.M.P. (2020). Current Advances in Nanotechnology and Medicine. NanoBioMedicine.

[5] Nikolova M., Slavchov R., Nikolova G., Hock F.J., Gralinski M.R. (2020). Nanotechnology in Medicine. Drug Discovery and Evaluation: Methods in Clinical Pharmacology.

[6] Contera S., Bernardino de la Serna J., Tetley T.D. (2020). Biotechnology, Nanotechnology and Medicine. Emerg. Top. Life Sci..

[7] Can Karanlık C., Aguilar-Galindo F., Sobotta L., Güzel E., Erdoğmuş A. (2023). Combination of Light and Ultrasound: Exploring Sono–Photochemical Activities of Phthalocyanine-Based Sensitizers. J. Phys. Chem. C.

[8] Ziental D., Mlynarczyk D.T., Kolasinski E., Güzel E., Dlugaszewska J., Popenda Ł., Jurga S., Goslinski T., Sobotta L. (2022). Zinc(II), Palladium(II), and Metal-Free Phthalocyanines Bearing Nipagin-Functionalized Substituents against *Candida auris* and Selected Multidrug-Resistant Microbes. Pharmaceutics.

[9] Güzel E., Atmaca G.Y., Kuznetsov A.E., Turkkol A., Bilgin M.D., Erdoğmuş A. (2022). Ultrasound versus Light: Exploring Photophysicochemical and Sonochemical Properties of Phthalocyanine-Based Therapeutics, Theoretical Study, and In Vitro Evaluations. ACS Appl. Bio Mater..

[10] Cheng H.-Y., Chu K.-T., Shen F.-C., Pan Y.-N., Chou H.-H., Ou K.-L. (2013). Stress Effect on Bone Remodeling and Osseointegration on Dental Implant with Novel Nano/Microporous Surface Functionalization. J. Biomed. Mater. Res..

[11] Aksakal B., Kom M., Tosun H.B., Demirel M. (2014). Influence of Micro- and Nano-Hydroxyapatite Coatings on the Osteointegration of Metallic (Ti6Al4 V) and Bioabsorbable Interference Screws: An in Vivo Study. Eur. J. Orthop. Surg. Traumatol..

[12] Min J.H., Kwon H.K., Kim B.I. (2011). The Addition of Nano-Sized Hydroxyapatite to a Sports Drink to Inhibit Dental Erosion—In Vitro Study Using Bovine Enamel. J. Dent..

[13] Danelon M., Pessan J.P., Neto F.N.S., de Camargo E.R., Delbem A.C.B. (2015). Effect of Toothpaste with Nano-Sized Trimetaphosphate on Dental Caries: In Situ Study. J. Dent..

[14] Taha S.T., Han H., Chang S.-R., Sovadinova I., Kuroda K., Langford R.M., Clarkson B.H. (2015). Nano/Micro Fluorhydroxyapatite Crystal Pastes in the Treatment of Dentin Hypersensitivity: An in Vitro Study. Clin. Oral Investig..

[15] Vano M., Derchi G., Barone A., Genovesi A., Covani U. (2015). Tooth Bleaching with Hydrogen Peroxide and Nano-Hydroxyapatite: A 9-Month Follow-up Randomized Clinical Trial. Int. J. Dent. Hyg..

[16] Kim Y.S., Kwon H.K., Kim B.I. (2011). Effect of Nano-Carbonate Apatite to Prevent Re-Stain after Dental Bleaching in Vitro. J. Dent..

[17] Generosi A., Rau J.V., Rossi Albertini V., Paci B. (2010). Crystallization Process of Carbonate Substituted Hydroxyapatite Nanoparticles in Toothpastes upon Physiological Conditions: An in Situ Time-Resolved X-Ray Diffraction Study. J. Mater. Sci. Mater. Med..

[18] Orsini G., Procaccini M., Manzoli L., Giuliodori F., Lorenzini A., Putignano A. (2010). A Double-Blind Randomized-Controlled Trial Comparing the Desensitizing Efficacy of a New Dentifrice Containing Carbonate/Hydroxyapatite Nanocrystals and a Sodium Fluoride/Potassium Nitrate Dentifrice: A New Dentifrice Desensitizing Efficacy. J. Clin. Periodontol..

[19] Thorat S.B., Diaspro A., Salerno M. (2014). In Vitro Investigation of Coupling-Agent-Free Dental Restorative Composite Based on Nano-Porous Alumina Fillers. J. Dent..

[20] Melo M.A., Guedes S.F., Xu H.H., Rodrigues L.K. (2013). Nanotechnology-Based Restorative Materials for Dental Caries Management. Trends Biotechnol..

[21] Meire M.A., Coenye T., Nelis H.J., De Moor R.J.G. (2012). Evaluation of Nd:YAG and Er:YAG Irradiation, Antibacterial Photodynamic Therapy and Sodium Hypochlorite Treatment on *Enterococcus faecalis* Biofilms: Laser-Assisted Killing of *E. faecalis* Biofilms. Int. Endod. J..

[22] Biel M. (2006). Advances in Photodynamic Therapy for the Treatment of Head and Neck Cancers. Lasers Surg. Med..

[23] Biel M.A., Gomer C.J. (2010). Photodynamic Therapy of Head and Neck Cancers. Photodynamic Therapy: Methods and Protocols.

[24] Srikanth K.B., Sharma D.M., Murru K., Gopu D.B.N., Gulia D.S.K., Nandini D.A.K., Tiwari D.H. (2021). Knowledge of Academic Omfs in Regenerative Nanotechnology in Field of Oral and Maxillofacial Surgery: A Questionnaire Survey. Ann. Rom. Soc. Cell Biol..

[25] Deyhle H., Bunk O., Müller B. (2011). Nanostructure of Healthy and Caries-Affected Human Teeth. Nanomed. Nanotechnol. Biol. Med..

[26] Chung C.-J., Wu B.-H., Lin J.-F., Han C.-F., Chuang S.-F., Li W.-L. (2011). Nano-Structure and Nano-Mechanical Properties of Human Teeth. Proceedings of the 2011 6th IEEE International Conference on Nano/Micro Engineered and Molecular Systems.

[27] Beniash E., Stifler C.A., Sun C.-Y., Jung G.S., Qin Z., Buehler M.J., Gilbert P.U.P.A. (2019). The Hidden Structure of Human Enamel. Nat. Commun..

[28] Cheong Y., Choi S., Kim S.J., Park H.-K. (2012). Nanostructural Effect of Acid-Etching and Fluoride Application on Human Primary and Permanent Tooth Enamels. Mater. Sci. Eng. C.

[29] Lacruz R.S., Habelitz S., Wright J.T., Paine M.L. (2017). Dental Enamel Formation and Implications for Oral Health and Disease. Physiol. Rev..

[30] Oliveira C.A., Bergqvist L.P., Line S.R.P. (2001). A Comparative Analysis of the Structure of the Dentinoenamel Junction in Mammals. J. Oral Sci..

[31] Kerebel B., Daculsi G., Kerebel L.M. (1979). Ultrastructural Studies of Enamel Crystallites. J. Dent. Res..

[32] Zelic K., Milovanovic P., Rakocevic Z., Askrabic S., Potocnik J., Popovic M., Djuric M. (2014). Nano-Structural and Compositional Basis of Devitalized Tooth Fragility. Dent. Mater..

[33] Li M.-Y. (2012). Contemporary Approach to Dental Caries.

[34] Anusavice K.J., Phillips R.W., Shen C., Rawls H.R. (2013). Phillips’ Science of Dental Materials.

[35] Burrow M.F. (2013). Composite Adhesive Restorative Materials for Dental Applications. Non-Metallic Biomaterials for Tooth Repair and Replacement.

[36] Ferracane J.L. (2011). Resin Composite—State of the Art. Dent. Mater..

[37] Coutinho E., Cardoso M.V., De Munck J., Neves A.A., Van Landuyt K.L., Poitevin A., Peumans M., Lambrechts P., Van Meerbeek B. (2009). Bonding Effectiveness and Interfacial Characterization of a Nano-Filled Resin-Modified Glass-Ionomer. Dent. Mater..

[38] Falsafi A., Mitra S.B., Oxman J.D., Ton T.T., Bui H.T. (2014). Mechanisms of Setting Reactions and Interfacial Behavior of a Nano-Filled Resin-Modified Glass Ionomer. Dent. Mater..

[39] Xu H.H.K., Moreau J.L., Sun L., Chow L.C. (2011). Nanocomposite Containing Amorphous Calcium Phosphate Nanoparticles for Caries Inhibition. Dent. Mater..

[40] Farrugia C., Camilleri J. (2015). Antimicrobial Properties of Conventional Restorative Filling Materials and Advances in Antimicrobial Properties of Composite Resins and Glass Ionomer Cements—A Literature Review. Dent. Mater..

[41] Liu F., Wang R., Shi Y., Jiang X., Sun B., Zhu M. (2013). Novel Ag Nanocrystals Based Dental Resin Composites with Enhanced Mechanical and Antibacterial Properties. Prog. Nat. Sci. Mater. Int..

[42] Tavassoli Hojati S., Alaghemand H., Hamze F., Ahmadian Babaki F., Rajab-Nia R., Rezvani M.B., Kaviani M., Atai M. (2013). Antibacterial, Physical and Mechanical Properties of Flowable Resin Composites Containing Zinc Oxide Nanoparticles. Dent. Mater..

[43] Taheri M.M., Abdul Kadir M.R., Shokuhfar T., Hamlekhan A., Shirdar M.R., Naghizadeh F. (2015). Fluoridated Hydroxyapatite Nanorods as Novel Fillers for Improving Mechanical Properties of Dental Composite: Synthesis and Application. Mater. Des..

[44] Eick J.D., Kotha S.P., Chappelow C.C., Kilway K.V., Giese G.J., Glaros A.G., Pinzino C.S. (2007). Properties of Silorane-Based Dental Resins and Composites Containing a Stress-Reducing Monomer. Dent. Mater..

[45] Xu H.H.K., Eichmiller F.C., Antonucci J.M., Schumacher G.E., Ives L.K. (2000). Dental Resin Composites Containing Ceramic Whiskers and Precured Glass Ionomer Particles. Dent. Mater..

[46] Xu H.H., Quinn J.B., Smith D.T., Giuseppetti A.A., Eichmiller F.C. (2003). Effects of Different Whiskers on the Reinforcement of Dental Resin Composites. Dent. Mater..

[47] Houshyar A., Khavandi A.R., Javadpour J., Samani S., Naimi-Jamal M.R., Atai M. (2013). Enhancement of Mechanical Properties of Experimental Composite by Fuller’s Earth Nanofibers for Cervical Restoration: Experimental Composite Enhancement by FE Nanofiber for Cervical Restoration. J. Biomed. Mater. Res. Part B Appl. Biomater..

[48] Mucci V., Pérez J., Vallo C.I. (2011). Preparation and Characterization of Light-Cured Methacrylate/Montmorillonite Nanocomposites. Polym. Int..

[49] Wang H., Zhu M., Li Y., Zhang Q., Wang H. (2011). Mechanical Properties of Dental Resin Composites by Co-Filling Diatomite and Nanosized Silica Particles. Mater. Sci. Eng. C.

[50] Atai M., Pahlavan A., Moin N. (2012). Nano-Porous Thermally Sintered Nano Silica as Novel Fillers for Dental Composites. Dent. Mater..

[51] Afsharnezhad S., Kashefi M., Behravan J., Ehtesham Gharaee M., Meshkat M., Shahrokh Abadi K., Homayoni Tabrizi M. (2014). Investigation of Nano-SiO_2_ Impact on Mechanical and Biocompatibility Properties of Cyanoacryalate Based Nanocomposites for Dental Application. Int. J. Adhes. Adhes..

[52] Zhang F., Xia Y., Xu L., Gu N. (2008). Surface Modification and Microstructure of Single-Walled Carbon Nanotubes for Dental Resin-Based Composites. J. Biomed. Mater. Res. Part B Appl. Biomater..

[53] Silva R.M., Pereira F.V., Mota F.A.P., Watanabe E., Soares S.M.C.S., Santos M.H. (2016). Dental Glass Ionomer Cement Reinforced by Cellulose Microfibers and Cellulose Nanocrystals. Mater. Sci. Eng. C.

[54] Yu B., Lim H.-N., Lee Y.-K. (2010). Influence of Nano- and Micro-Filler Proportions on the Optical Property Stability of Experimental Dental Resin Composites. Mater. Des..

[55] Miao X., Zhu M., Li Y., Zhang Q., Wang H. (2012). Synthesis of Dental Resins Using Diatomite and Nano-Sized SiO_2_ and TiO_2_. Prog. Nat. Sci. Mater. Int..

[56] Durner J., Dębiak M., Bürkle A., Hickel R., Reichl F.-X. (2011). Induction of DNA Strand Breaks by Dental Composite Components Compared to X-Ray Exposure in Human Gingival Fibroblasts. Arch. Toxicol..

[57] Durner J., Stojanovic M., Urcan E., Hickel R., Reichl F.-X. (2011). Influence of Silver Nano-Particles on Monomer Elution from Light-Cured Composites. Dent. Mater..

[58] Durner J., Obermaier J., Draenert M., Ilie N. (2012). Correlation of the Degree of Conversion with the Amount of Elutable Substances in Nano-Hybrid Dental Composites. Dent. Mater..

[59] Mayworm C.D., Camargo S.S., Bastian F.L. (2008). Influence of Artificial Saliva on Abrasive Wear and Microhardness of Dental Composites Filled with Nanoparticles. J. Dent..

[60] Korbmacher-Steiner H.M., Schilling A.F., Huck L.G., Kahl-Nieke B., Amling M. (2013). Laboratory Evaluation of Toothbrush/Toothpaste Abrasion Resistance after Smooth Enamel Surface Sealing. Clin. Oral Investig..

[61] Turssi C., Ferracane J., Vogel K. (2005). Filler Features and Their Effects on Wear and Degree of Conversion of Particulate Dental Resin Composites. Biomaterials.

[62] Elsaesser A., Howard C.V. (2012). Toxicology of Nanoparticles. Adv. Drug Deliv. Rev..

[63] Van Landuyt K.L., Yoshihara K., Geebelen B., Peumans M., Godderis L., Hoet P., Van Meerbeek B. (2012). Should We Be Concerned about Composite (Nano-)Dust?. Dent. Mater..

[64] Van Noort R., Barbour M.E. (2013). Introduction to Dental Materials.

[65] Calvo A.F.B., Alves F.B.T., Lenzi T.L., Tedesco T.K., Reis A., Loguercio A.D., Raggio D.P. (2014). Glass Ionomer Cements Bond Stability in Caries-Affected Primary Dentin. Int. J. Adhes. Adhes..

[66] Zhang K., Cheng L., Imazato S., Antonucci J.M., Lin N.J., Lin-Gibson S., Bai Y., Xu H.H.K. (2013). Effects of Dual Antibacterial Agents MDPB and Nano-Silver in Primer on Microcosm Biofilm, Cytotoxicity and Dentine Bond Properties. J. Dent..

[67] Melo M.A.S., Cheng L., Zhang K., Weir M.D., Rodrigues L.K.A., Xu H.H.K. (2013). Novel Dental Adhesives Containing Nanoparticles of Silver and Amorphous Calcium Phosphate. Dent. Mater..

[68] Toledano M., Sauro S., Cabello I., Watson T., Osorio R. (2013). A Zn-Doped Etch-and-Rinse Adhesive May Improve the Mechanical Properties and the Integrity at the Bonded-Dentin Interface. Dent. Mater..

[69] Vasant S.R., Joshi M.J. (2011). Synthesis and Characterization of Pure and Zinc Doped Calcium Pyrophosphate Dihydrate Nanoparticles. Eur. Phys. J. Appl. Phys..

[70] Lohbauer U., Wagner A., Belli R., Stoetzel C., Hilpert A., Kurland H.-D., Grabow J., Müller F.A. (2010). Zirconia Nanoparticles Prepared by Laser Vaporization as Fillers for Dental Adhesives. Acta Biomater..

[71] Belli R., Kreppel S., Petschelt A., Hornberger H., Boccaccini A.R., Lohbauer U. (2014). Strengthening of Dental Adhesives via Particle Reinforcement. J. Mech. Behav. Biomed. Mater..

[72] Yoshida S., Sugii H., Itoyama T., Kadowaki M., Hasegawa D., Tomokiyo A., Hamano S., Ipposhi K., Yamashita K., Maeda H. (2021). Development of a Novel Direct Dental Pulp-Capping Material Using 4-META/MMA-TBB Resin with Nano Hydroxyapatite. Mater. Sci. Eng. C.

[73] Walton R.E., Torabinejad M. (2008). Endodontics Principles and Practice.

[74] Koprowicz A., Koprowicz P. (2021). Efficacy of Finisher Files in the Removal of Calcium Hydroxide Paste from the Root Canal System—Preliminary Results. JMS.

[75] Lim Z., Cheng J., Lim T., Teo E., Wong J., George S., Kishen A. (2009). Light Activated Disinfection: An Alternative Endodontic Disinfection Strategy. Aust. Dent. J..

[76] Jurič I.B., Plečko V., Pandurić D.G., Anić I. (2014). The Antimicrobial Effectiveness of Photodynamic Therapy Used as an Addition to the Conventional Endodontic Re-Treatment: A Clinical Study. Photodiagn. Photodyn. Ther..

[77] Siqueira J.F., Machado A.G., Silveira R.M., Lopes H.P., Uzeda M. (1997). de Evaluation of the Effectiveness of Sodium Hypochlorite Used with Three Irrigation Methods in the Elimination of *Enterococcus faecalis* from the Root Canal, in Vitro. Int. Endod. J..

[78] Berutti E., Marini R., Angeretti A. (1997). Penetration Ability of Different Irrigants into Dentinal Tubules. J. Endod..

[79] Peters L.B., Wesselink P.R., Buijs J.F., Van Winkelhoff A.J. (2001). Viable Bacteria in Root Dentinal Tubules of Teeth with Apical Periodontitis. J. Endod..

[80] Portenier I., Waltimo T.M., Haapasalo M. (2003). *Enterococcus faecalis*–The Root Canal Survivor and ‘Star’ in Post-Treatment Disease. Endod. Top..

[81] Athanassiadis B., Abbott P., Walsh L. (2007). The Use of Calcium Hydroxide, Antibiotics and Biocides as Antimicrobial Medicaments in Endodontics. Aust. Dent. J..

[82] Fisher K., Phillips C. (2009). The Ecology, Epidemiology and Virulence of Enterococcus. Microbiology.

[83] Cieplik F., Tabenski L., Buchalla W., Maisch T. (2014). Antimicrobial Photodynamic Therapy for Inactivation of Biofilms Formed by Oral Key Pathogens. Front. Microbiol..

[84] de Oliveira B., Aguiar C., Camara A. (2014). Photodynamic Therapy in Combating the Causative Microorganisms from Endodontic Infections. Eur. J. Dent..

[85] Sobotta L., Dlugaszewska J., Kasprzycki P., Lijewski S., Teubert A., Mielcarek J., Gdaniec M., Goslinski T., Fita P., Tykarska E. (2018). In Vitro Photodynamic Activity of Lipid Vesicles with Zinc Phthalocyanine Derivative against *Enterococcus faecalis*. J. Photochem. Photobiol. B Biol..

[86] Sobotta L., Sniechowska J., Ziental D., Dlugaszewska J., Potrzebowski M.J. (2019). Chlorins with (Trifluoromethyl)Phenyl Substituents—Synthesis, Lipid Formulation and Photodynamic Activity against Bacteria. Dye. Pigment..

[87] Sobotta L., Dlugaszewska J., Gierszewski M., Tillo A., Sikorski M., Tykarska E., Mielcarek J., Goslinski T. (2018). Photodynamic Inactivation of *Enterococcus faecalis* by Non-Peripherally Substituted Magnesium Phthalocyanines Entrapped in Lipid Vesicles. J. Photochem. Photobiol. B Biol..

[88] Sobotta L., Skupin-Mrugalska P., Piskorz J., Mielcarek J. (2019). Non-Porphyrinoid Photosensitizers Mediated Photodynamic Inactivation against Bacteria. Dye. Pigment..

[89] Sobotta L., Dlugaszewska J., Ziental D., Szczolko W., Koczorowski T., Goslinski T., Mielcarek J. (2019). Optical Properties of a Series of Pyrrolyl-Substituted Porphyrazines and Their Photoinactivation Potential against *Enterococcus faecalis* after Incorporation into Liposomes. J. Photochem. Photobiol. A Chem..

[90] Czarczynska-Goslinska B., Stolarska M., Ziental D., Falkowski M., Glowacka-Sobotta A., Dlugaszewska J., Goslinski T., Sobotta L. (2021). Photodynamic Antimicrobial Activity of Magnesium(II) Porphyrazine with Bulky Peripheral Sulfanyl Substituents. Phosphorus Sulfur Silicon Relat. Elem..

[91] Sobotta L., Ziental D., Sniechowska J., Dlugaszewska J., Potrzebowski M.J. (2019). Lipid Vesicle-Loaded Meso-Substituted Chlorins of High in Vitro Antimicrobial Photodynamic Activity. Photochem. Photobiol. Sci..

[92] Sobotta L., Skupin-Mrugalska P., Piskorz J., Mielcarek J. (2019). Porphyrinoid Photosensitizers Mediated Photodynamic Inactivation against Bacteria. Eur. J. Med. Chem..

[93] Sobotta L., Lijewski S., Dlugaszewska J., Nowicka J., Mielcarek J., Goslinski T. (2019). Photodynamic Inactivation of *Enterococcus faecalis* by Conjugates of Zinc(II) Phthalocyanines with Thymol and Carvacrol Loaded into Lipid Vesicles. Inorg. Chim. Acta.

[94] Sobotta L., Skupin-Mrugalska P., Mielcarek J., Goslinski T., Balzarini J. (2015). Photosensitizers Mediated Photodynamic Inactivation Against Virus Particles. Mini-Rev. Med. Chem..

[95] Ziental D., Zajac J., Lewandowski K., Dlugaszewska J., Potrzebowski M.J., Sobotta L. (2022). Oxospirochlorins as New Promising Photosensitizers against Priority Pathogens. Dye. Pigment..

[96] Stolarska M., Glowacka-Sobotta A., Ziental D., Dlugaszewska J., Falkowski M., Goslinski T., Sobotta L. (2021). Photochemical Properties and Promising Activity against Staphylococci of Sulfanyl Porphyrazines with Dendrimeric Moieties. Inorg. Chim. Acta.

[97] Stolarska M., Glowacka-Sobotta A., Ziental D., Dlugaszewska J., Falkowski M., Mielcarek J., Goslinski T., Sobotta L. (2021). Photochemical Properties and Photocytotoxicities against Wound Bacteria of Sulfanyl Porphyrazines with Bulky Peripheral Substituents. J. Organomet. Chem..

[98] Ziental D., Mlynarczyk D.T., Czarczynska-Goslinska B., Lewandowski K., Sobotta L. (2021). Photosensitizers Mediated Photodynamic Inactivation against Fungi. Nanomaterials.

[99] Stolarska M., Glowacka-Sobotta A., Mlynarczyk D.T., Dlugaszewska J., Goslinski T., Mielcarek J., Sobotta L. (2020). Photodynamic Activity of Tribenzoporphyrazines with Bulky Periphery against Wound Bacteria. Int. J. Mol. Sci..

[100] Proffit W.R., Fields H.W. (2000). Contemporary Orthodontics.

[101] Pinheiro S.L., da Silva J.N., Gonçalves R.O., Villalpando K.T. (2014). Manual and Rotary Instrumentation Ability to Reduce *Enterococcus faecalis* Associated with Photodynamic Therapy in Deciduous Molars. Braz. Dent. J..

[102] Pinheiro S.L., Schenka A.A., Neto A.A., de Souza C.P., Rodriguez H.M.H., Ribeiro M.C. (2009). Photodynamic Therapy in Endodontic Treatment of Deciduous Teeth. Lasers Med. Sci..

[103] Da Silva Barbosa P., Duarte D.A., Leite M.F., de Sant’ Anna G.R. (2014). Photodynamic Therapy in Pediatric Dentistry. Case Rep. Dent..

[104] De Sant’Anna G.R. (2014). Photodynamic Therapy for the Endodontic Treatment of a Traumatic Primary Tooth in a Diabetic Pediatric Patient. J. Dent. Res. Dent. Clin. Dent. Prospect..

[105] Carvalho E.d.S., Mello I., Albergaria S.J., Habitante S.M., Lage-Marques J.L., Raldi D.P. (2011). Effect of Chemical Substances in Removing Methylene Blue After Photodynamic Therapy in Root Canal Treatment. Photomed. Laser Surg..

[106] Späth A., Leibl C., Cieplik F., Lehner K., Regensburger J., Hiller K.-A., Bäumler W., Schmalz G., Maisch T. (2014). Improving Photodynamic Inactivation of Bacteria in Dentistry: Highly Effective and Fast Killing of Oral Key Pathogens with Novel Tooth-Colored Type-II Photosensitizers. J. Med. Chem..

[107] Tennert C., Feldmann K., Haamann E., Al-Ahmad A., Follo M., Wrbas K.-T., Hellwig E., Altenburger M.J. (2014). Effect of Photodynamic Therapy (PDT) on *Enterococcus faecalis* Biofilm in Experimental Primary and Secondary Endodontic Infections. BMC Oral Health.

[108] Da Frota M.F., Guerreiro-Tanomaru J.M., Tanomaru-Filho M., Bagnato V.S., Espir C.G., Berbert F.L.C.V. (2015). Photodynamic Therapy in Root Canals Contaminated with *Enterococcus faecalis* Using Curcumin as Photosensitizer. Lasers Med. Sci..

[109] Muhammad O.H., Chevalier M., Rocca J.-P., Brulat-Bouchard N., Medioni E. (2014). Photodynamic Therapy versus Ultrasonic Irrigation: Interaction with Endodontic Microbial Biofilm, an Ex Vivo Study. Photodiagn. Photodyn. Ther..

[110] Kranz S., Guellmar A., Völpel A., Gitter B., Albrecht V., Sigusch B.W. (2011). Photodynamic Suppression of *Enterococcus faecalis* Using the Photosensitizer MTHPC. Lasers Surg. Med..

[111] Ossmann A., Kranz S., Andre G., Völpel A., Albrecht V., Fahr A., Sigusch B.W. (2015). Photodynamic Killing of *Enterococcus faecalis* in Dentinal Tubules Using MTHPC Incorporated in Liposomes and Invasomes. Clin. Oral Investig..

[112] Bumb S.S. (2014). Assessment of Photodynamic Therapy (PDT) in Disinfection of Deeper Dentinal Tubules in a Root Canal System: An In Vitro Study. J. Clin. Diagn. Res..

[113] Pagonis T.C., Chen J., Fontana C.R., Devalapally H., Ruggiero K., Song X., Foschi F., Dunham J., Skobe Z., Yamazaki H. (2010). Nanoparticle-Based Endodontic Antimicrobial Photodynamic Therapy. J. Endod..

[114] Muthalib S., Verma A.H., Sundar S., Sampath Kumar T.S., Velmurugan N., Krithikadatta J. (2020). Evaluation of Effect of Two Different Functionalized Nanoparticle Photodynamic Therapy on Nanohardness of Root Dentin—An in Vitro Study. Photodiagn. Photodyn. Ther..

[115] Sabino C.P., Garcez A.S., Núñez S.C., Ribeiro M.S., Hamblin M.R. (2015). Real-Time Evaluation of Two Light Delivery Systems for Photodynamic Disinfection of *Candida albicans* Biofilm in Curved Root Canals. Lasers Med. Sci..

[116] Garcez A.S., Fregnani E.R., Rodriguez H.M., Nunez S.C., Sabino C.P., Suzuki H., Ribeiro M.S. (2013). The Use of Optical Fiber in Endodontic Photodynamic Therapy. Is It Really Relevant?. Lasers Med. Sci..

[117] Garcez A.S., Ribeiro M.S., Tegos G.P., Núñez S.C., Jorge A.O.C., Hamblin M.R. (2007). Antimicrobial Photodynamic Therapy Combined with Conventional Endodontic Treatment to Eliminate Root Canal Biofilm Infection. Lasers Surg. Med..

[118] Cheng X., Guan S., Lu H., Zhao C., Chen X., Li N., Bai Q., Tian Y., Yu Q. (2012). Evaluation of the Bactericidal Effect of Nd:YAG, Er:YAG, Er,Cr:YSGG Laser Radiation, and Antimicrobial Photodynamic Therapy (APDT) in Experimentally Infected Root Canals. Lasers Surg. Med..

[119] Turrioni A.P.S., de Oliveira C.F., Basso F.G., Moriyama L.T., Kurachi C., Hebling J., Bagnato V.S., de Souza Costa C.A. (2012). Correlation between Light Transmission and Permeability of Human Dentin. Lasers Med. Sci..

[120] Arias-Moliz M.T., Baca P., Solana C., Toledano M., Medina-Castillo A.L., Toledano-Osorio M., Osorio R. (2021). Doxycycline-functionalized Polymeric Nanoparticles Inhibit *Enterococcus faecalis* Biofilm Formation on Dentine. Int. Endod. J..

[121] Kim T.-S., Bürklin T., Schacher B., Ratka-Krüger P., Schaecken M.T., Renggli H.H., Fiehn W., Eickholz P. (2002). Pharmacokinetic Profile of a Locally Administered Doxycycline Gel in Crevicular Fluid, Blood, and Saliva. J. Periodontol..

[122] Porter M.L.A., Münchow E.A., Albuquerque M.T.P., Spolnik K.J., Hara A.T., Bottino M.C. (2016). Effects of Novel 3-Dimensional Antibiotic-Containing Electrospun Scaffolds on Dentin Discoloration. J. Endod..

[123] Rasimick B.J., Wan J., Musikant B.L., Deutsch A.S. (2010). Stability of Doxycycline and Chlorhexidine Absorbed on Root Canal Dentin. J. Endod..

[124] Bulavinets T., Kulpa-Greszta M., Tomaszewska A., Kus-Liśkiewicz M., Bielatowicz G., Yaremchuk I., Barylyak A., Bobitski Y., Pązik R. (2020). Efficient NIR Energy Conversion of Plasmonic Silver Nanostructures Fabricated with the Laser-Assisted Synthetic Approach for Endodontic Applications. RSC Adv..

[125] Karczewski A., Feitosa S.A., Hamer E.I., Pankajakshan D., Gregory R.L., Spolnik K.J., Bottino M.C. (2018). Clindamycin-Modified Triple Antibiotic Nanofibers: A Stain-Free Antimicrobial Intracanal Drug Delivery System. J. Endod..

[126] Baras B.H., Sun J., Melo M.A.S., Tay F.R., Oates T.W., Zhang K., Weir M.D., Xu H.H.K. (2019). Novel Root Canal Sealer with Dimethylaminohexadecyl Methacrylate, Nano-Silver and Nano-Calcium Phosphate to Kill Bacteria inside Root Dentin and Increase Dentin Hardness. Dent. Mater..

[127] Seung J., Weir M.D., Melo M.A.S., Romberg E., Nosrat A., Xu H.H.K., Tordik P.A. (2018). A Modified Resin Sealer: Physical and Antibacterial Properties. J. Endod..

[128] Ioannidis K., Niazi S., Mylonas P., Mannocci F., Deb S. (2019). The Synthesis of Nano Silver-Graphene Oxide System and Its Efficacy against Endodontic Biofilms Using a Novel Tooth Model. Dent. Mater..

[129] Akhavan O., Ghaderi E., Esfandiar A. (2011). Wrapping Bacteria by Graphene Nanosheets for Isolation from Environment, Reactivation by Sonication, and Inactivation by Near-Infrared Irradiation. J. Phys. Chem. B.

[130] Akhavan O., Ghaderi E. (2010). Toxicity of Graphene and Graphene Oxide Nanowalls Against Bacteria. ACS Nano.

[131] Beyth N., Kesler Shvero D., Zaltsman N., Houri-Haddad Y., Abramovitz I., Davidi M.P., Weiss E.I. (2013). Rapid Kill—Novel Endodontic Sealer and *Enterococcus faecalis*. PLoS ONE.

[132] Guerreiro Tanomaru J.M., Storto I., Da Silva G.F., Bosso R., Costa B.C., Bernardi M.I.B., Tanomaru-Filho M. (2014). Radiopacity, PH and Antimicrobial Activity of Portland Cement Associated with Micro- and Nanoparticles of Zirconium Oxide and Niobium Oxide. Dent. Mater. J..

[133] Suwanprateeb J., Thammarakcharoen F., Wasoontararat K., Chokevivat W., Phanphiriya P. (2012). Preparation and Characterization of Nanosized Silver Phosphate Loaded Hydroxyapatite by Single Step Co-Conversion Process. Mater. Sci. Eng. C.

[134] Collares F.M., Leitune V.C.B., Rostirolla F.V., Trommer R.M., Bergmann C.P., Samuel S.M.W. (2012). Nanostructured Hydroxyapatite as Filler for Methacrylate-Based Root Canal Sealers: HAnano in Root Canal Sealers. Int. Endod. J..

[135] Saghiri M.A., Asatourian A., Orangi J., Lotfi M., Soukup J.W., Garcia-Godoy F., Sheibani N. (2015). Effect of Particle Size on Calcium Release and Elevation of PH of Endodontic Cements. Dent. Traumatol..

[136] Saghiri M.A., Asgar K., Lotfi M., Garcia-Godoy F. (2012). Nanomodification of Mineral Trioxide Aggregate for Enhanced Physiochemical Properties: Nanomodification of Mineral Trioxide Aggregate. Int. Endod. J..

[137] Saghiri M.A., Garcia-Godoy F., Gutmann J.L., Lotfi M., Asatourian A., Ahmadi H. (2013). Push-out Bond Strength of a Nano-Modified Mineral Trioxide Aggregate. Dent. Traumatol..

[138] Naseri M., Eftekhar L., Gholami F., Atai M., Dianat O. (2019). The Effect of Calcium Hydroxide and Nano–Calcium Hydroxide on Microhardness and Superficial Chemical Structure of Root Canal Dentin: An Ex Vivo Study. J. Endod..

[139] Opačić-Galić V., Petrović V., Živković S., Jokanović V., Nikolić B., Knežević-Vukčević J., Mitić-Ćulafić D. (2013). New Nanostructural Biomaterials Based on Active Silicate Systems and Hydroxyapatite: Characterization and Genotoxicity in Human Peripheral Blood Lymphocytes. Int. Endod. J..

[140] Saeed F., Muhammad N., Khan A.S., Sharif F., Rahim A., Ahmad P., Irfan M. (2020). Prosthodontics Dental Materials: From Conventional to Unconventional. Mater. Sci. Eng. C.

[141] Ghazal M., Hedderich J., Kern M. (2008). Wear of Feldspathic Ceramic, Nano-Filled Composite Resin and Acrylic Resin Artificial Teeth When Opposed to Different Antagonists. Eur. J. Oral Sci..

[142] Zheng J., Su Q., Wang C., Cheng G., Zhu R., Shi J., Yao K. (2011). Synthesis and Biological Evaluation of PMMA/MMT Nanocomposite as Denture Base Material. J. Mater. Sci. Mater. Med..

[143] Wang X., Su Q., Hu Y., Wang C., Zheng J. (2014). Structure and Thermal Stability of PMMA/MMT Nanocomposites as Denture Base Material. J. Therm. Anal. Calorim..

[144] Turagam N., Prasad Mudrakola D. (2013). Effect of Micro-Additions of Carbon Nanotubes to Polymethylmethacrylate on Reduction in Polymerization Shrinkage: Nanotubes Overcome Acrylic Resin Shrinkage. J. Prosthodont..

[145] Dağistan S., Aktas A.E., Caglayan F., Ayyildiz A., Bilge M. (2009). Differential Diagnosis of Denture-Induced Stomatitis, *Candida*, and Their Variations in Patients Using Complete Denture: A Clinical and Mycological Study. Mycoses.

[146] Chandra J., Mukherjee P.K., Leidich S.D., Faddoul F.F., Hoyer L.L., Douglas L.J., Ghannoum M.A. (2001). Antifungal Resistance of Candidal Biofilms Formed on Denture Acrylic in Vitro. J. Dent. Res..

[147] He X.Y., Meurman J.H., Kari K., Rautemaa R., Samaranayake L.P. (2006). In Vitro Adhesion of *Candida* Species to Denture Base Materials. Mycoses.

[148] Panáček A., Kolář M., Večeřová R., Prucek R., Soukupová J., Kryštof V., Hamal P., Zbořil R., Kvítek L. (2009). Antifungal Activity of Silver Nanoparticles against *Candida* spp.. Biomaterials.

[149] Wady A.F., Machado A.L., Zucolotto V., Zamperini C.A., Berni E., Vergani C.E. (2012). Evaluation of *Candida albicans* Adhesion and Biofilm Formation on a Denture Base Acrylic Resin Containing Silver Nanoparticles: Antifungal Activity of Silver Nanoparticles. J. Appl. Microbiol..

[150] Nam K.-Y., Lee C.-H., Lee C.-J. (2012). Antifungal and Physical Characteristics of Modified Denture Base Acrylic Incorporated with Silver Nanoparticles: Antifungal Denture Base with Silver. Gerodontology.

[151] Berezow A.B., Darveau R.P. (2011). Microbial Shift and Periodontitis: Microbial Shift. Periodontol. 2000.

[152] Larjava H., Yang Y., Putnins E., Heino J., Häkkinen L. (2012). Biological Agents and Cell Therapies in Periodontal Regeneration: Biological Agents and Cell Therapies in Periodontal Regeneration. Endod Top..

[153] Soukos N.S., Goodson J.M. (2011). Photodynamic Therapy in the Control of Oral Biofilms: Photodynamic Therapy in the Control of Oral Biofilms. Periodontol. 2000.

[154] Meisel P., Kocher T. (2005). Photodynamic Therapy for Periodontal Diseases: State of the Art. J. Photochem. Photobiol. B Biol..

[155] Takasaki A.A., Aoki A., Mizutani K., Schwarz F., Sculean A., Wang C.-Y., Koshy G., Romanos G., Ishikawa I., Izumi Y. (2009). Application of Antimicrobial Photodynamic Therapy in Periodontal and Peri-Implant Diseases. Periodontol. 2000.

[156] Malik R., Manocha A., Suresh D. (2010). Photodynamic Therapy—A Strategic Review. Indian J. Dent. Res..

[157] Pfitzner A., Sigusch B.W., Albrecht V., Glockmann E. (2004). Killing of Periodontopathogenic Bacteria by Photodynamic Therapy. J. Periodontol..

[158] Matevski D., Weersink R., Tenenbaum H.C., Wilson B., Ellen R.P., Lépine G. (2003). Lethal Photosensitization of Periodontal Pathogens by a Red-Filtered Xenon Lamp in Vitro: Lethal Photosensitization. J. Periodontal Res..

[159] Sigusch B.W., Pfitzner A., Albrecht V., Glockmann E. (2005). Efficacy of Photodynamic Therapy on Inflammatory Signs and Two Selected Periodontopathogenic Species in a Beagle Dog Model. J. Periodontol..

[160] Polansky R., Haas M., Heschl A., Wimmer G. (2009). Clinical Effectiveness of Photodynamic Therapy in the Treatment of Periodontitis. J. Clin. Periodontol..

[161] Rühling A., Fanghänel J., Houshmand M., Kuhr A., Meisel P., Schwahn C., Kocher T. (2010). Photodynamic Therapy of Persistent Pockets in Maintenance Patients—A Clinical Study. Clin. Oral Investig..

[162] Queiroz A.C., Suaid F.A., de Andrade P.F., Novaes A.B., Taba M., Palioto D.B., Grisi M.F.M., Souza S.L.S. (2014). Antimicrobial Photodynamic Therapy Associated to Nonsurgical Periodontal Treatment in Smokers: Microbiological Results. J. Photochem. Photobiol. B Biol..

[163] Lulic M., Leiggener Görög I., Salvi G.E., Ramseier C.A., Mattheos N., Lang N.P. (2009). One-Year Outcomes of Repeated Adjunctive Photodynamic Therapy during Periodontal Maintenance: A Proof-of-Principle Randomized-Controlled Clinical Trial. J. Clin. Periodontol..

[164] Bottura P.E., Milanezi J., Fernandes L.A., Caldas H.C., Abbud-Filho M., Garcia V.G., Baptista M.A.S.F. (2011). Nonsurgical Periodontal Therapy Combined with Laser and Photodynamic Therapies for Periodontal Disease in Immunosuppressed Rats. Transplant. Proc..

[165] Braun A., Dehn C., Krause F., Jepsen S. (2008). Short-Term Clinical Effects of Adjunctive Antimicrobial Photodynamic Therapy in Periodontal Treatment: A Randomized Clinical Trial. J. Clin. Periodontol..

[166] Christodoulides N., Nikolidakis D., Chondros P., Becker J., Schwarz F., Rössler R., Sculean A. (2008). Photodynamic Therapy as an Adjunct to Non-Surgical Periodontal Treatment: A Randomized, Controlled Clinical Trial. J. Periodontol..

[167] Giannelli M., Formigli L., Lorenzini L., Bani D. (2012). Combined Photoablative and Photodynamic Diode Laser Therapy as an Adjunct to Non-Surgical Periodontal Treatment. A Randomized Split-Mouth Clinical Trial. J. Clin. Periodontol..

[168] Sigusch B.W., Engelbrecht M., Völpel A., Holletschke A., Pfister W., Schütze J. (2010). Full-Mouth Antimicrobial Photodynamic Therapy in Fusobacterium Nucleatum–Infected Periodontitis Patients. J. Periodontol..

[169] Abuderman A.W.A., Muzaheed (2021). Antibacterial Effectiveness of Scaling and Root Planing with and without Photodynamic Therapy against *Campylobacter rectus* Counts in the Oral Biofilm of Patients with Periodontitis. Photodiagn. Photodyn. Ther..

[170] Al-Hamoudi N., Mokeem S., Shafqat S.S., Vohra F., Abduljabbar T. (2021). Effectiveness of Antimicrobial Photodynamic Therapy as an Adjunct to Open Flap Debridement in Patients with Aggressive Periodontitis. Photodiagn. Photodyn. Ther..

[171] de Oliveira R.R., Schwartz-Filho H.O., Novaes A.B., Taba M. (2007). Antimicrobial Photodynamic Therapy in the Non-Surgical Treatment of Aggressive Periodontitis: A Preliminary Randomized Controlled Clinical Study. J. Periodontol..

[172] Al-Zahrani M.S., Bamshmous S.O., Alhassani A.A., Al-Sherbini M.M. (2009). Short-Term Effects of Photodynamic Therapy on Periodontal Status and Glycemic Control of Patients With Diabetes. J. Periodontol..

[173] De Almeida J.M., Theodoro L.H., Bosco A.F., Nagata M.J.H., Bonfante S., Garcia V.G. (2008). Treatment of Experimental Periodontal Disease by Photodynamic Therapy in Rats With Diabetes. J. Periodontol..

[174] Chitsazi M.T., Shirmohammadi A., Pourabbas R., Abolfazli N., Farhoudi I., Daghigh Azar B., Farhadi F. (2014). Clinical and Microbiological Effects of Photodynamic Therapy Associated with Non-Surgical Treatment in Aggressive Periodontitis. J. Dent. Res. Dent. Clin. Dent. Prospect..

[175] Braham P., Herron C., Street C., Darveau R. (2009). Antimicrobial Photodynamic Therapy May Promote Periodontal Healing Through Multiple Mechanisms. J. Periodontol..

[176] Chen B., Wu W., Sun W., Zhang Q., Yan F., Xiao Y. (2014). RANKL Expression in Periodontal Disease: Where Does RANKL Come From?. BioMed Res. Int..

[177] De Oliveira R.R., Schwartz-Filho H.O., Novaes A.B., Garlet G.P., Freitas de Souza R., Taba M., Scombatti de Souza S.L., Ribeiro F.J. (2009). Antimicrobial Photodynamic Therapy in the Non-Surgical Treatment of Aggressive Periodontitis: Cytokine Profile in Gingival Crevicular Fluid, Preliminary Results. J. Periodontol..

[178] Giannopoulou C., Cappuyns I., Cancela J., Cionca N., Mombelli A. (2012). Effect of Photodynamic Therapy, Diode Laser, and Deep Scaling on Cytokine and Acute-Phase Protein Levels in Gingival Crevicular Fluid of Residual Periodontal Pockets. J. Periodontol..

[179] Lui J., Corbet E.F., Jin L. (2011). Combined Photodynamic and Low-Level Laser Therapies as an Adjunct to Nonsurgical Treatment of Chronic Periodontitis: Photodynamic and Low-Level Laser Therapies for Periodontitis. J. Periodontal Res..

[180] Monzavi A., Chinipardaz Z., Mousavi M., Fekrazad R., Moslemi N., Azaripour A., Bagherpasand O., Chiniforush N. (2016). Antimicrobial Photodynamic Therapy Using Diode Laser Activated Indocyanine Green as an Adjunct in the Treatment of Chronic Periodontitis: A Randomized Clinical Trial. Photodiagn. Photodyn. Ther..

[181] Hayakumo S., Arakawa S., Mano Y., Izumi Y. (2013). Clinical and Microbiological Effects of Ozone Nano-Bubble Water Irrigation as an Adjunct to Mechanical Subgingival Debridement in Periodontitis Patients in a Randomized Controlled Trial. Clin. Oral Investig..

[182] Johnston D., Kumar P., Choonara Y.E., du Toit L.C., Pillay V. (2013). Modulation of the Nano-Tensile Mechanical Properties of Co-Blended Amphiphilic Alginate Fibers as Oradurable Biomaterials for Specialized Biomedical Application. J. Mech. Behav. Biomed. Mater..

[183] Bozzini B., Barca A., Bogani F., Boniardi M., Carlino P., Mele C., Verri T., Romano A. (2014). Electrodeposition of Nanostructured Bioactive Hydroxyapatite-Heparin Composite Coatings on Titanium for Dental Implant Applications. J. Mater. Sci. Mater. Med..

[184] Meng W., Zhou Y., Zhang Y., Cai Q., Yang L., Zhao J., Li C. (2011). Osteoblast Behavior on Hierarchical Micro-/Nano-Structured Titanium Surface. J. Bionic Eng..

[185] Metzler P., von Wilmowsky C., Stadlinger B., Zemann W., Schlegel K.A., Rosiwal S., Rupprecht S. (2013). Nano-Crystalline Diamond-Coated Titanium Dental Implants—A Histomorphometric Study in Adult Domestic Pigs. J. Cranio-Maxillofac. Surg..

[186] Chaturvedi T. (2009). An Overview of the Corrosion Aspect of Dental Implants (Titanium and Its Alloys). Indian J. Dent. Res..

[187] Lee Y.-H., Bhattarai G., Aryal S., Lee N.-H., Lee M.-H., Kim T.-G., Jhee E.-C., Kim H.-Y., Yi H.-K. (2010). Modified Titanium Surface with Gelatin Nano Gold Composite Increases Osteoblast Cell Biocompatibility. Appl. Surf. Sci..

[188] Shibli J.A., Martins M.C., Ribeiro F.S., Garcia V.G., Nociti F.H., Marcantonio E. (2006). Lethal Photosensitization and Guided Bone Regeneration in Treatment of Peri-Implantitis: An Experimental Study in Dogs. Clin. Oral Implant. Res.

[189] Meirelles L., Albrektsson T., Kjellin P., Arvidsson A., Franke-Stenport V., Andersson M., Currie F., Wennerberg A. (2008). Bone Reaction to Nano Hydroxyapatite Modified Titanium Implants Placed in a Gap-Healing Model. J. Biomed. Mater. Res..

[190] Palmquist A., Lindberg F., Emanuelsson L., Brånemark R., Engqvist H., Thomsen P. (2010). Biomechanical, Histological, and Ultrastructural Analyses of Laser Micro- and Nano-Structured Titanium Alloy Implants: A Study in Rabbit. J. Biomed. Mater. Res..

[191] Freitas G.P., Lopes H.B., Martins-Neto E.C., de Oliveira P.T., Beloti M.M., Rosa A.L. (2016). Effect of Surface Nanotopography on Bone Response to Titanium Implant. J. Oral Implantol..

[192] De Barros R.R.M., Novaes A.B., Queiroz A., de Almeida A.L.G. (2012). Early Peri-Implant Endosseous Healing of Two Implant Surfaces Placed in Surgically Created Circumferential Defects. A Histomorphometric and Fluorescence Study in Dogs. Clin. Oral Impl. Res..

[193] Yokota S., Kurihara J., Nishiwaki N., Tamate S., Ueda K., Narushima T., Kawamura H., Sasaki K., Suzuki O., Takahashi N. (2012). Accelerated Bone Formation Around Titanium Dental Implants with Amorphous Calcium Phosphate Coating in Rabbits. Interface Oral Health Science 2011.

[194] Ueda K., Narushima T., Goto T., Katsube T., Nakagawa H., Kawamura H., Taira M. (2007). Evaluation of Calcium Phosphate Coating Films on Titanium Fabricated Using RF Magnetron Sputtering. Mater. Trans..

[195] Alghamdi H.S., AJA van Oirschot B., Bosco R., van den Beucken J.J.J.P., Aldosari A.A.F., Anil S., Jansen J.A. (2013). Biological Response to Titanium Implants Coated with Nanocrystals Calcium Phosphate or Type 1 Collagen in a Dog Model. Clin. Oral Impl. Res..

[196] De Wilde E.A.W.J., Jimbo R., Wennerberg A., Naito Y., Coucke P., Bryington M.S., Vandeweghe S., De Bruyn H. (2015). The Soft Tissue Immunologic Response to Hydroxyapatite-Coated Transmucosal Implant Surfaces: A Study in Humans: Immunologic Soft Tissue Response to Nanostructures. Clin. Implant Dent. Relat. Res..

[197] Zhao S., Dong W., Jiang Q., He F., Wang X., Yang G. (2013). Effects of Zinc-Substituted Nano-Hydroxyapatite Coatings on Bone Integration with Implant Surfaces. J. Zhejiang Univ. Sci. B.

[198] Salarian M., Xu W.Z., Wang Z., Sham T.-K., Charpentier P.A. (2014). Hydroxyapatite–TiO_2_-Based Nanocomposites Synthesized in Supercritical CO_2_ for Bone Tissue Engineering: Physical and Mechanical Properties. ACS Appl. Mater. Interfaces.

[199] Jin C., Ren L., Ding H., Shi G., Lin H., Zhang F. (2012). Enhanced Attachment, Proliferation, and Differentiation of Human Gingival Fibroblasts on Titanium Surface Modified with Biomolecules. J. Biomed. Mater. Res..

[200] Mehdikhani-Nahrkhalaji M., Fathi M.H., Mortazavi V., Mousavi S.B., Hashemi-Beni B., Razavi S.M. (2012). Novel Nanocomposite Coating for Dental Implant Applications in Vitro and in Vivo Evaluation. J. Mater. Sci. Mater. Med..

[201] Elias C.N., Fernandes D.J., Resende C.R.S., Roestel J. (2015). Mechanical Properties, Surface Morphology and Stability of a Modified Commercially Pure High Strength Titanium Alloy for Dental Implants. Dent. Mater..

[202] Wang F., Shi L., He W.-X., Han D., Yan Y., Niu Z.-Y., Shi S.-G. (2013). Bioinspired Micro/Nano Fabrication on Dental Implant–Bone Interface. Appl. Surf. Sci..

[203] Moon S.-K., Kwon J.-S., Uhm S.-H., Lee E.-J., Gu H.-J., Eom T.-G., Kim K.-N. (2014). Biological Evaluation of Micro–Nano Patterned Implant Formed by Anodic Oxidation. Curr. Appl. Phys..

[204] Al Qahtani M.S.A., Wu Y., Spintzyk S., Krieg P., Killinger A., Schweizer E., Stephan I., Scheideler L., Geis-Gerstorfer J., Rupp F. (2015). UV-A and UV-C Light Induced Hydrophilization of Dental Implants. Dent. Mater..

[205] Schär D., Ramseier C.A., Eick S., Arweiler N.B., Sculean A., Salvi G.E. (2013). Anti-Infective Therapy of Peri-Implantitis with Adjunctive Local Drug Delivery or Photodynamic Therapy: Six-Month Outcomes of a Prospective Randomized Clinical Trial. Clin. Oral Impl. Res..

[206] Huang J., Li X., Koller G.P., Di Silvio L., Vargas-Reus M.A., Allaker R.P. (2011). Electrohydrodynamic Deposition of Nanotitanium Doped Hydroxyapatite Coating for Medical and Dental Applications. J. Mater. Sci. Mater. Med..

[207] Marotti J., Tortamano P., Cai S., Ribeiro M.S., Franco J.E.M., de Campos T.T. (2013). Decontamination of Dental Implant Surfaces by Means of Photodynamic Therapy. Lasers Med. Sci..

[208] König K., Teschke M., Sigusch B., Glockmann E., Eick S., Pfister W. (2000). Red Light Kills Bacteria via Photodynamic Action. Cell Mol. Biol..

[209] Mortazavi V., Nahrkhalaji M.M., Fathi M.H., Mousavi S.B., Esfahani B.N. (2010). Antibacterial Effects of Sol-Gel-Derived Bioactive Glass Nanoparticle on Aerobic Bacteria. J. Biomed. Mater. Res..

[210] Dorkhan M., Hall J., Uvdal P., Sandell A., Svensäter G., Davies J.R. (2014). Crystalline Anatase-Rich Titanium Can Reduce Adherence of Oral Streptococci. Biofouling.

[211] Dorkhan M., Yücel-Lindberg T., Hall J., Svensäter G., Davies J.R. (2014). Adherence of Human Oral Keratinocytes and Gingival Fibroblasts to Nano-Structured Titanium Surfaces. BMC Oral Health.

[212] Massa M.A., Covarrubias C., Bittner M., Fuentevilla I.A., Capetillo P., Von Marttens A., Carvajal J.C. (2014). Synthesis of New Antibacterial Composite Coating for Titanium Based on Highly Ordered Nanoporous Silica and Silver Nanoparticles. Mater. Sci. Eng. C.

[213] Godoy-Gallardo M., Rodríguez-Hernández A.G., Delgado L.M., Manero J.M., Javier Gil F., Rodríguez D. (2015). Silver Deposition on Titanium Surface by Electrochemical Anodizing Process Reduces Bacterial Adhesion of *Streptococcus sanguinis* and *Lactobacillus salivarius*. Clin. Oral Impl. Res..

[214] Memarzadeh K., Sharili A.S., Huang J., Rawlinson S.C.F., Allaker R.P. (2015). Nanoparticulate Zinc Oxide as a Coating Material for Orthopedic and Dental Implants: Nanoparticulate ZnO as a Coating Material. J. Biomed. Mater. Res..

[215] Hayek R.R.A., Araújo N.S., Gioso M.A., Ferreira J., Baptista-Sobrinho C.A., Yamada A.M., Ribeiro M.S. (2005). Comparative Study Between the Effects of Photodynamic Therapy and Conventional Therapy on Microbial Reduction in Ligature-Induced Peri-Implantitis in Dogs. J. Periodontol..

[216] Li Y., Gao Y., Shao B., Xiao J., Hu K., Kong L. (2012). Effects of Hydrofluoric Acid and Anodised Micro and Micro/Nano Surface Implants on Early Osseointegration in Rats. Br. J. Oral Maxillofac. Surg..

[217] Wang X., Zhou Y., Xia L., Zhao C., Chen L., Yi D., Chang J., Huang L., Zheng X., Zhu H. (2015). Fabrication of Nano-Structured Calcium Silicate Coatings with Enhanced Stability, Bioactivity and Osteogenic and Angiogenic Activity. Colloids Surf. B Biointerfaces.

[218] Vaidya P., Mahale S., Kale S., Patil A. (2017). Osseointegration—A Review. IOSR J. Dent. Med. Sci..

[219] Carlsson L., Röstlund T., Albrektsson B., Albrektsson T., Brånemark P.-I. (1986). Osseointegration of Titanium Implants. Acta Orthop. Scand..

[220] Kim T.-I. (2014). A Tribute to Dr. Per-Ingvar Brånemark. J. Periodontal. Implant. Sci..

[221] Pye A.D., Lockhart D.E.A., Dawson M.P., Murray C.A., Smith A.J. (2009). A Review of Dental Implants and Infection. J. Hosp. Infect..

[222] Albrektsson T., Johansson C. (2001). Osteoinduction, Osteoconduction and Osseointegration. Eur. Spine J..

[223] Guglielmotti M.B., Olmedo D.G., Cabrini R.L. (2019). Research on Implants and Osseointegration. Periodontol. 2000.

[224] Alghamdi H.S. (2018). Methods to Improve Osseointegration of Dental Implants in Low Quality (Type-IV) Bone: An Overview. J. Funct. Biomater..

[225] Zhang W., Wang G., Liu Y., Zhao X., Zou D., Zhu C., Jin Y., Huang Q., Sun J., Liu X. (2013). The Synergistic Effect of Hierarchical Micro/Nano-Topography and Bioactive Ions for Enhanced Osseointegration. Biomaterials.

[226] Faria P.E.P., Felipucci D.N.B., Simioni A.R., Primo F.L., Tedesco A.C., Salata L.A. (2015). Effects of Photodynamic Process (PDP) in Implant Osseointegration: A Histologic and Histometric Study in Dogs: Photodynamic Process in Implant Osseointegration. Clin. Implant Dent. Relat. Res..

[227] Chung S., Milligan M., Gong S.-G. (2015). Photobiostimulation as a Modality to Accelerate Orthodontic Tooth Movement. Semin. Orthod..

[228] Nandagopal N., Usha M., Sreejith S., Rajan S. (2021). A Clinical Review of Nanotechnology in Maxillofacial Practice. J. Oral Res. Rev..

[229] Ver Halen J., Naylor T., Petersen D. (2014). Current and Future Applications of Nanotechnology in Plastic and Reconstructive Surgery. Plast. Aesthet. Res..

[230] Romero-Reyes M., Uyanik J.M. (2014). Orofacial Pain Management: Current Perspectives. J. Pain Res..

[231] Verma H., Tandon P. (2020). Application of Nanotechnology in Oral and Maxillofacial Surgery. IOSR J. Dent. Med. Sci..

[232] Verma S.K., Chauhan R. (2014). Nanorobotics in Dentistry—A Review. Indian J. Dent..

[233] Shakib K., Tan A., Soskic V., Seifalian A.M. (2014). Regenerative Nanotechnology in Oral and Maxillofacial Surgery. Br. J. Oral Maxillofac. Surg..

[234] Tavakol S., Nikpour M.R., Amani A., Soltani M., Rabiee S.M., Rezayat S.M., Chen P., Jahanshahi M. (2013). Bone Regeneration Based on Nano-Hydroxyapatite and Hydroxyapatite/Chitosan Nanocomposites: An in Vitro and in Vivo Comparative Study. J. Nanopart. Res..

[235] Tavakol S., Nikpour M.R., Hoveizi E., Tavakol B., Rezayat S.M., Adabi M., Shajari Abokheili S., Jahanshahi M. (2014). Investigating the Effects of Particle Size and Chemical Structure on Cytotoxicity and Bacteriostatic Potential of Nano Hydroxyapatite/Chitosan/Silica and Nano Hydroxyapatite/Chitosan/Silver; as Antibacterial Bone Substitutes. J. Nanopart. Res..

[236] Balhuc S., Campian R., Labunet A., Negucioiu M., Buduru S., Kui A. (2021). Dental Applications of Systems Based on Hydroxyapatite Nanoparticles—An Evidence-Based Update. Crystals.

[237] Wang L., Liu Q., Jing D., Zhou S., Shao L. (2014). Biomechanical Properties of Nano-TiO_2_ Addition to a Medical Silicone Elastomer: The Effect of Artificial Ageing. J. Dent..

[238] Kardach H., Olszewska A., Firlej E., Bogdanowicz A., Golusińska-Kardach E., Szponar-Żurowska A., Biedziak B. (2019). Force Decay of Intermaxillary Orthodontic Elastics: In Vitro Study. JMS.

[239] Kumarasinghe L.S., Ninan N., Dabare P.R.L., Cavallaro A., Doğramacı E.J., Rossi-Fedele G., Dreyer C., Vasilev K., Zilm P. (2021). Bioactive Plasma Coatings on Orthodontic Brackets: In Vitro Metal Ion Release and Cytotoxicity. Coatings.

[240] Zhao T., Li Y., Liu Y., Zhao X. (2012). Nano-Hardness, Wear Resistance and Pseudoelasticity of Hafnium Implanted NiTi Shape Memory Alloy. J. Mech. Behav. Biomed. Mater..

[241] Khan A.S., Alshaia A., AlDubayan A., Alarifi S., Alamri A., Aldossary H., Ahmed S.Z., Ateeq I.S., Hakeem A.S., Rehman S. (2022). Preparation of Nano-Apatite Grafted Glass-Fiber-Reinforced Composites for Orthodontic Application: Mechanical and In Vitro Biofilm Analysis. Materials.

[242] Gracco A., Dandrea M., Deflorian F., Zanella C., De Stefani A., Bruno G., Stellini E. (2019). Application of a Molybdenum and Tungsten Disulfide Coating to Improve Tribological Properties of Orthodontic Archwires. Nanomaterials.

[243] Lin C.-W., Chung C.-J., Chou C.-M., He J.-L. (2016). Morphological Effect Governed by Sandblasting and Anodic Surface Reforming on the Super-Hydrophobicity of AISI 304 Stainless Steel. Thin Solid Film..

[244] Lin C.-W., Chung C.-J., Chou C.-M., He J.-L. (2019). In Vitro Wear Tests of the Dual-Layer Grid Blasting-Plasma Polymerized Superhydrophobic Coatings on Stainless Steel Orthodontic Substrates. Thin Solid Film..

[245] Al-Fadhily Z.M., Abdul-Hadi M. (2023). A Novel Coating of Orthodontic Archwires with Chlorhexidine Hexametaphosphate Nanoparticles. Int. J. Biomater..

[246] Gil F.J., Espinar-Escalona E., Clusellas N., Fernandez-Bozal J., Artes-Ribas M., Puigdollers A. (2020). New Bactericide Orthodonthic Archwire: NiTi with Silver Nanoparticles. Metals.

[247] Farhadian N., Usefi Mashoof R., Khanizadeh S., Ghaderi E., Farhadian M., Miresmaeili A. (2016). *Streptococcus mutans* Counts in Patients Wearing Removable Retainers with Silver Nanoparticles vs Those Wearing Conventional Retainers: A Randomized Clinical Trial. Am. J. Orthod. Dentofac. Orthop..

[248] Alam M.K., Ganji K.K. (2021). Nano-Bio Fusion Gingival Gel in the Management of Fixed Orthodontic Treatment-Induced Gingivitis: An Empirical Study. Am. J. Orthod. Dentofac. Orthop..

[249] Hosseinzadeh-Nik T., Karimzadeh A., Ayatollahi M.R. (2013). Bond Strength of a Nano-Composite Used for Bonding Ceramic Orthodontic Brackets. Mater. Des..

[250] Fadaie P., Atai M., Imani M., Karkhaneh A., Ghasaban S. (2013). Cyanoacrylate–POSS Nanocomposites: Novel Adhesives with Improved Properties for Dental Applications. Dent. Mater..

[251] Jahanbin A., Farzanegan F., Atai M., Jamehdar S.A., Golfakhrabadi P., Shafaee H. (2017). A Comparative Assessment of Enamel Mineral Content and *Streptococcus mutans* Population between Conventional Composites and Composites Containing Nano Amorphous Calcium Phosphate in Fixed Orthodontic Patients: A Split-Mouth Randomized Clinical Trial. EORTHO.

[252] Liu Y., Zhang L., Niu L., Yu T., Xu H.H.K., Weir M.D., Oates T.W., Tay F.R., Chen J. (2018). Antibacterial and Remineralizing Orthodontic Adhesive Containing Quaternary Ammonium Resin Monomer and Amorphous Calcium Phosphate Nanoparticles. J. Dent..

[253] Behnaz M., Fahiminejad N., Amdjadi P., Yedegari Z., Dalaie K., Dastgir R. (2022). Evaluation and Comparison of Antibacterial and Physicochemical Properties of Synthesized Zinc Oxide-Nano Particle-Containing Adhesive with Commercial Adhesive: An Experimental Study. Int. Orthod..

[254] Eslamian L., Borzabadi-Farahani A., Karimi S., Saadat S., Badiee M.R. (2020). Evaluation of the Shear Bond Strength and Antibacterial Activity of Orthodontic Adhesive Containing Silver Nanoparticle, an In-Vitro Study. Nanomaterials.

[255] Yi J., Weir M.D., Melo M.A.S., Li T., Lynch C.D., Oates T.W., Dai Q., Zhao Z., Xu H.H.K. (2019). Novel Rechargeable Nano-CaF_2_ Orthodontic Cement with High Levels of Long-Term Fluoride Release. J. Dent..

[256] Karimzadeh A., Ayatollahi M.R., Hosseinzadeh-Nik T. (2015). Effects of a Nano-Composite Adhesive on Mechanical Properties of Tooth Enamel After Removing Orthodontics Bracket—An Experimental Study Using Nano-Indentation Test. Exp. Mech..

[257] Xu Y., Sun Y., Liu W., Shi Z., Jin X., Xu J., Pan X., Zhang Z., Fu B., Zhang L. (2023). Effects of an Orthodontic Primer Containing Amorphous Fluorinated Calcium Phosphate Nanoparticles on Enamel White Spot Lesions. J. Mech. Behav. Biomed. Mater..

[258] Soukos N.S., Mulholland S.E., Socransky S.S., Doukas A.G. (2003). Photodestruction of Human Dental Plaque Bacteria: Enhancement of the Photodynamic Effect by Photomechanical Waves in an Oral Biofilm Model. Lasers Surg. Med..

[259] Müller P., Guggenheim B., Schmidlin P.R. (2007). Efficacy of Gasiform Ozone and Photodynamic Therapy on a Multispecies Oral Biofilm in Vitro. Eur. J. Oral Sci..

[260] Fontana C.R., Abernethy A.D., Som S., Ruggiero K., Doucette S., Marcantonio R.C., Boussios C.I., Kent R., Goodson J.M., Tanner A.C.R. (2009). The Antibacterial Effect of Photodynamic Therapy in Dental Plaque-Derived Biofilms. J. Periodontal Res..

[261] Wysocki M., Czarczynska-Goslinska B., Ziental D., Michalak M., Güzel E., Sobotta L. (2022). Excited State and Reactive Oxygen Species against Cancer and Pathogens: A Review on Sonodynamic and Sono-Photodynamic Therapy. ChemMedChem.

[262] Liu P.-F., Zhu W.-H., Huang C.-M. (2009). Vaccines and Photodynamic Therapies for Oral Microbial-Related Diseases. CDM.

[263] Nagata J.Y., Hioka N., Kimura E., Batistela V.R., Terada R.S.S., Graciano A.X., Baesso M.L., Hayacibara M.F. (2012). Antibacterial Photodynamic Therapy for Dental Caries: Evaluation of the Photosensitizers Used and Light Source Properties. Photodiagn. Photodyn. Ther..

[264] Santin G.C., Oliveira D.S.B., Galo R., Borsatto M.C., Corona S.A.M. (2014). Antimicrobial Photodynamic Therapy and Dental Plaque: A Systematic Review of the Literature. Sci. World J..

[265] Qiao J., Wang S., Wen Y., Jia H. (2014). Photodynamic Effects on Human Periodontal-Related Cells in Vitro. Photodiagn. Photodyn. Ther..

[266] Ghinzelli G.C., Souza M.A., Cecchin D., Farina A.P., de Figueiredo J.A.P. (2014). Influence of Ultrasonic Activation on Photodynamic Therapy over Root Canal System Infected with *Enterococcus faecalis*—An in Vitro Study. Photodiagn. Photodyn. Ther..

[267] Diniz I.M.A., Horta I.D., Azevedo C.S., Elmadjian T.R., Matos A.B., Simionato M.R.L., Marques M.M. (2015). Antimicrobial Photodynamic Therapy: A Promise Candidate for Caries Lesions Treatment. Photodiagn. Photodyn. Ther..

[268] Lima J.P.M., Sampaio de Melo M.A., Borges F.M.C., Teixeira A.H., Steiner-Oliveira C., Nobre dos Santos M., Rodrigues L.K.A., Zanin I.C.J. (2009). Evaluation of the Antimicrobial Effect of Photodynamic Antimicrobial Therapy in an *In Situ* Model of Dentine Caries. Eur. J. Oral Sci..

[269] Lin J., Bi L.J., Zhang Z.G., Fu Y.M., Dong T.T. (2010). Toluidine Blue-Mediated Photodynamic Therapy of Oral Wound Infections in Rats. Lasers Med. Sci..

[270] Ichinose-Tsuno A., Aoki A., Takeuchi Y., Kirikae T., Shimbo T., Lee M.-C., Yoshino F., Maruoka Y., Itoh T., Ishikawa I. (2014). Antimicrobial Photodynamic Therapy Suppresses Dental Plaque Formation in Healthy Adults: A Randomized Controlled Clinical Trial. BMC Oral Health.

[271] Asnaashari M., Mojahedi S.M., Asadi Z., Azari-Marhabi S., Maleki A. (2016). A Comparison of the Antibacterial Activity of the Two Methods of Photodynamic Therapy (Using Diode Laser 810 Nm and LED Lamp 630 Nm) against *Enterococcus faecalis* in Extracted Human Anterior Teeth. Photodiagn. Photodyn. Ther..

[272] Chen C.-P., Chen C.-T., Tsai T. (2012). Chitosan Nanoparticles for Antimicrobial Photodynamic Inactivation: Characterization and In Vitro Investigation. Photochem. Photobiol..

[273] Pereira C.A., Costa A.C.B.P., Carreira C.M., Junqueira J.C., Jorge A.O.C. (2013). Photodynamic Inactivation of *Streptococcus mutans* and *Streptococcus sanguinis* Biofilms in Vitro. Lasers Med. Sci..

[274] Besinis A., De Peralta T., Handy R.D. (2014). Inhibition of Biofilm Formation and Antibacterial Properties of a Silver Nano-Coating on Human Dentine. Nanotoxicology.

[275] Marsich E., Bellomo F., Turco G., Travan A., Donati I., Paoletti S. (2013). Nano-Composite Scaffolds for Bone Tissue Engineering Containing Silver Nanoparticles: Preparation, Characterization and Biological Properties. J. Mater. Sci. Mater. Med..

[276] Ramazanzadeh B., Jahanbin A., Yaghoubi M., Shahtahmassbi N., Ghazvini K., Shakeri M., Shafaee H. (2015). Comparison of Antibacterial Effects of ZnO and CuO Nanoparticles Coated Brackets against *Streptococcus mutans*. J. Dent..

[277] Markopoulos A.K. (2012). Current Aspects on Oral Squamous Cell Carcinoma. Open Dent. J..

[278] Saini R., Lee N., Liu K., Poh C. (2016). Prospects in the Application of Photodynamic Therapy in Oral Cancer and Premalignant Lesions. Cancers.

[279] Ram H., Sarkar J., Kumar H., Konwar R., Bhatt M.L.B., Mohammad S. (2011). Oral Cancer: Risk Factors and Molecular Pathogenesis. J. Maxillofac. Oral Surg..

[280] Bredell M.G., Besic E., Maake C., Walt H. (2010). The Application and Challenges of Clinical PD–PDT in the Head and Neck Region: A Short Review. J. Photochem. Photobiol. B Biol..

[281] De Visscher S.A.H.J., Melchers L.J., Dijkstra P.U., Karakullukcu B., Tan I.B., Hopper C., Roodenburg J.L.N., Witjes M.J.H. (2013). MTHPC-Mediated Photodynamic Therapy of Early Stage Oral Squamous Cell Carcinoma: A Comparison to Surgical Treatment. Ann. Surg. Oncol..

[282] Karakullukcu B., Stoker S.D., Wildeman A.P.E., Copper M.P., Wildeman M.A., Tan I.B. (2013). A Matched Cohort Comparison of MTHPC-Mediated Photodynamic Therapy and Trans-Oral Surgery of Early Stage Oral Cavity Squamous Cell Cancer. Eur. Arch. Oto-Rhino-Laryngol..

[283] Anand S., Rollakanti K.R., Horst R.L., Hasan T., Maytin E.V. (2014). Combination of Oral Vitamin D_3_ with Photodynamic Therapy Enhances Tumor Cell Death in a Murine Model of Cutaneous Squamous Cell Carcinoma. Photochem. Photobiol..

[284] Bhuvaneswari R., Ng Q.F., Thong P.S.P., Soo K.-C. (2015). Nimotuzumab Increases the Anti-Tumor Effect of Photodynamic Therapy in an Oral Tumor Model. Oncotarget.

[285] He X., Hu N., Yang S., Yang Z., Hu L., Wang X., Wen N. (2022). Nimotuzumab Shows an Additive Effect to Inhibit Cell Growth of ALA-PDT Treated Oral Cancer Cells. Photodiagn. Photodyn. Ther..

[286] Chen W.-H., Lecaros R.L.G., Tseng Y.-C., Huang L., Hsu Y.-C. (2015). Nanoparticle Delivery of HIF1α SiRNA Combined with Photodynamic Therapy as a Potential Treatment Strategy for Head-and-Neck Cancer. Cancer Lett..

[287] He C., Liu D., Lin W. (2015). Self-Assembled Core–Shell Nanoparticles for Combined Chemotherapy and Photodynamic Therapy of Resistant Head and Neck Cancers. ACS Nano.

[288] Zhao H., Feng H., Liu D., Liu J., Ji N., Chen F., Luo X., Zhou Y., Dan H., Zeng X. (2015). Self-Assembling Monomeric Nucleoside Molecular Nanoparticles Loaded with 5-FU Enhancing Therapeutic Efficacy against Oral Cancer. ACS Nano.

[289] Wang B., Wang J.-H., Liu Q., Huang H., Chen M., Li K., Li C., Yu X.-F., Chu P.K. (2014). Rose-Bengal-Conjugated Gold Nanorods for in Vivo Photodynamic and Photothermal Oral Cancer Therapies. Biomaterials.

[290] Wang J., Wang K., Liang J., Jin J., Wang X., Yan S. (2021). Chitosan-Tripolyphosphate Nanoparticles-Mediated Co-Delivery of MTHFD1L ShRNA and 5-Aminolevulinic Acid for Combination Photodynamic-Gene Therapy in Oral Cancer. Photodiagn. Photodyn. Ther..

[291] Yang S.-J., Lin C.-F., Kuo M.-L., Tan C.-T. (2013). Photodynamic Detection of Oral Cancers with High-Performance Chitosan-Based Nanoparticles. Biomacromolecules.

[292] Romeo U. (2014). Oral Proliferative Verrucous Leukoplakia Treated with the Photodynamic Therapy: A Case Report. Ann. Stomatol..

[293] He L., Deng D., Zhou X., Cheng L., ten Cate J.M., Li J., Li X., Crielaard W. (2015). Novel Tea Polyphenol-Modified Calcium Phosphate Nanoparticle and Its Remineralization Potential: Remineralization of tea polyphenol-modified nanoparticles. J. Biomed. Mater. Res..

[294] Targino A.G.R., Flores M.A.P., dos Santos Junior V.E., de Godoy Bené Bezerra F., de Luna Freire H., Galembeck A., Rosenblatt A. (2014). An Innovative Approach to Treating Dental Decay in Children. A New Anti-Caries Agent. J. Mater. Sci. Mater. Med..

[295] dos Santos V.E., Filho A.V., Ribeiro Targino A.G., Pelagio Flores M.A., Galembeck A., Caldas A.F., Rosenblatt A. (2014). A New “Silver-Bullet” to Treat Caries in Children—Nano Silver Fluoride: A Randomised Clinical Trial. J. Dent..

[296] West N., Seong J., Davies M., Lussi A., Ganss C. (2014). Dentine Hypersensitivity. Monographs in Oral Science.

[297] Clark M.B., Slayton R.L., Segura A., Boulter S., Clark M.B., Gereige R., Krol D., Mouradian W., Quinonez R., Section on Oral Health (2014). Fluoride Use in Caries Prevention in the Primary Care Setting. Pediatrics.

[298] Hill R.G., Gillam D.G., Chen X. (2015). The Ability of a Nano Hydroxyapatite Toothpaste and Oral Rinse Containing Fluoride to Protect Enamel during an Acid Challenge Using 19F Solid State NMR Spectroscopy. Mater. Lett..

[299] Bruzell E.M., Pallesen U., Thoresen N.R., Wallman C., Dahl J.E. (2013). Side Effects of External Tooth Bleaching: A Multi-Centre Practice-Based Prospective Study. Br. Dent. J..

[300] Sreenivasalu P.K.P., Dora C.P., Swami R., Jasthi V.C., Shiroorkar P.N., Nagaraja S., Asdaq S.M.B., Anwer M.d.K. (2022). Nanomaterials in Dentistry: Current Applications and Future Scope. Nanomaterials.

